# Butterfly-Shaped Dibenz[*a*,*j*]anthracenes: Synthesis and Photophysical Properties

**DOI:** 10.1021/acs.orglett.3c02306

**Published:** 2023-08-25

**Authors:** Yan-Ying Wu, Yi-Lin Wu, Cheng-Lan Lin, Hung-Cheng Chen, Yao-Yuan Chuang, Chih-Hsien Chen, Chih-Ming Chou

**Affiliations:** † Department of Applied Chemistry, National University of Kaohsiung, Kaohsiung 81148, Taiwan; ‡ School of Chemistry, Cardiff University, Main Building, Park Place, Cardiff CF10 3AT, United Kingdom; § Department of Chemical and Materials Engineering, Tamkang University, New Taipei City 251301, Taiwan; ∥ Department of Chemical Engineering, Feng Chia University, Taichung 407, Taiwan

## Abstract

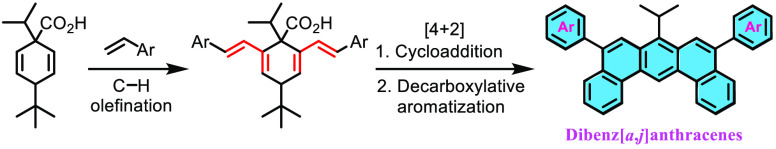

A strategy for the synthesis of dibenz[*a*,*j*]anthracenes (DBAs) from cyclohexa-2,5-diene-1-carboxylic acids is presented. Our approach involves sequential C–H olefination, cycloaddition, and decarboxylative aromatization. In the key step for DBA skeleton construction, the bis-C–H olefination products, 1,3-dienes, are utilized as substrates for [4 + 2] cycloaddition with benzyne. This concise synthetic route allows for regioselective ring formation and functional group introduction. The structural features and photophysical properties of the resulting DBA molecules are discussed.

The quest for new synthetic strategies for polycyclic aromatic hydrocarbons (PAHs) has been motivated by the rich chemistry that they have exhibited over the past 50 years. PAHs, known for their π-conjugated electronic structure, have long been a testing ground for theoretical developments concerning aromaticity, open-shell radicals, and quantum chemical calculations.^1^ These π-extended aromatic molecules possess a low energy gap between the frontier orbitals, particularly in the ultraviolet–visible–near-infrared (UV–vis–NIR) region, and feature accessible redox chemistry and non-covalent interactions. Consequently, PAHs have emerged as highly promising materials for diverse applications in optoelectronics, sensing, and energy conversion.^2^

While linearly ring-fused PAHs (acenes) exhibit intriguing potential for applications such as field-effect transistors and singlet exciton fission,^3^ their practical utilization has been impeded by their inherent instability. Substantial efforts have thus been directed toward exploring two-dimensional or angularly fused PAHs.^4^ A notable example is the class of angularly fused bis(tetracene) derivatives. These compounds were found to exhibit enhanced stability under ambient conditions and successfully employed as air-stable organic semiconductors in field-effect transistors and organic photovoltaics.^5^ Additionally, helicenes, featuring *ortho*-fused aromatic rings, display interesting electronic and optical properties, including circularly polarized luminescence and electron spin filter behavior, as a result of their structural chirality.^6^ These advancements have been achieved in conjunction with the pursuit of structurally and topologically precise subunits of graphene,^7^ enabling precise control over their electronic and optical properties, and have fueled enthusiasm for the development of novel PAH-based materials.

In this context, dibenz[*a*,*j*]anthracene (DBA; Scheme 1) can be considered a particularly interesting PAH as a result of the combination of linearly and angularly fused ring architecture. This underdeveloped class of PAHs has been utilized to build *J*-aggregating emissive macrocycles^8^ and rigid molecular receptors,^9^ has served as a precursor to construct the zigzag edge of topologically specific graphitic nanoribbons,^10^ and can be regarded as a building block for coronoid hydrocarbons.^11^ The early approach to construct such aromatic systems involved photocyclization^12^ or Diels–Alder reaction^13^ (Scheme 1a); however, these methods often result in a mixture of regioisomers. More recently, ring-closing metathesis^14^ and electrophilic cyclization^10a,15^ from olefinic and acetylenic precursors, respectively, have been explored to achieve better control over product geometry. The elaborated precursors used in these methods limit the introduction of functionalities to DBA, and in some cases, specific sites of the starting materials need to be blocked to ensure regiospecificity.

**Scheme 1:**
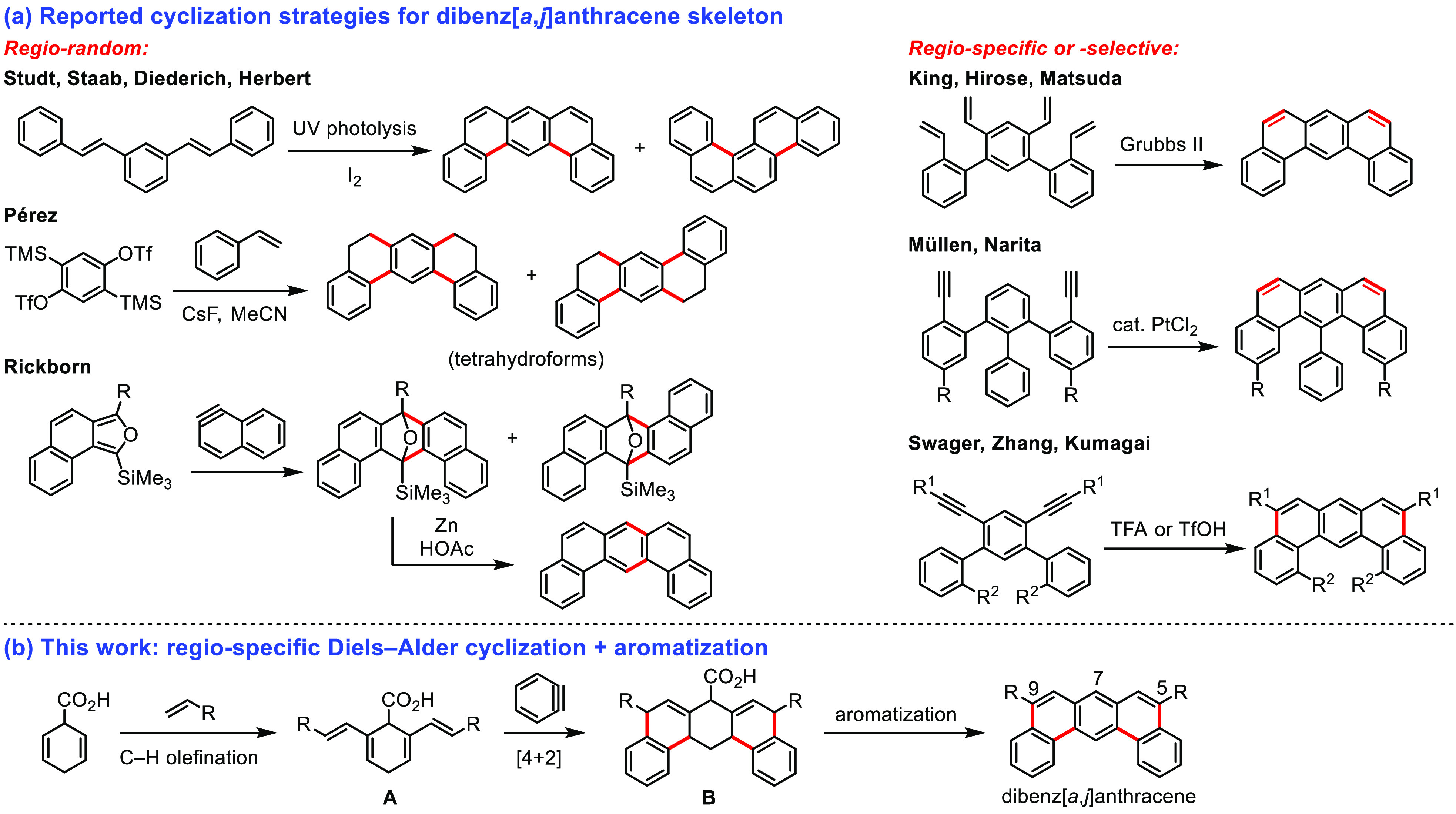
Synthetic Strategies for DBA

Building upon our recently developed protocols for C(alkenyl)–H functionalizations of cyclohexa-2,5-diene-1-carboxylic acids (**A**; Scheme 1b),^16^ we envisioned that constructing the angularly fused ring skeleton from this bilateral 1,3-diene would prescribe the Diels–Alder reaction with a high level of regiochemical control. The Pd-coordinative carboxylic acid functionality (a “transient directing group”) could aid in introducing desired functionalities and enable decarboxylative aromatization of proaromatic compound **B**, ultimately yielding DBA. In light of this possibility, we present here a regiospecific DBA synthesis through tandem Pd-catalyzed directed C–H olefination, Diels–Alder cyclization, and oxidative aromatization. This method enables the introduction of functional groups at positions 5 and 9, and the size of angularly fused PAH can be varied by selecting the appropriate aryne precursor for the Diels–Alder reaction. Furthermore, we discuss the mechanism of formation, structural properties, UV–vis absorption and emission, and electrochemical properties of these DBA derivatives.

The synthesis of 5,9-disubstituted DBA commenced with a sequential carboxylate-directed Pd-catalyzed C(alkenyl)–H olefination of 4-(*t*-butyl)-1-*i*-propylcyclohexa-2,5-diene-1-carboxylic acid (**1**; Scheme 2). The *t*-butyl group of the starting material reduces the tendency of competing decarboxylative aromatization in the stage of olefination to give compounds **2** and **3**. Thus, the sequential olefination can occur under a similar condition to afford symmetric and asymmetric bilateral 1,3-diene **3** using our Pd-catalyzed C–H activation protocols in good yields over two steps.^16b^ After esterification with benzyl bromide, bis(butadiene) **4** was then treated with *in situ* generated benzyne or 2-naphthyne to give proaromatic intermediate **5** in 37–80% yields. The electronic characteristics of the butadiene moiety show little bearing on the outcome of the Diels–Alder reaction of compound **4**. On the other hand, the low thermal stability of naphthyne may contribute to the low yield for compound **5g**. After deprotection of the benzyl group, in the final stage of the synthesis, 2,3-dichloro-5,6-dicyano-1,4-benzoquinone (DDQ) was exploited to achieve decarboxylative (the central ring) and oxidative (the “angled” rings) aromatization of compound **5** to give the DBA ring system (**6**) in 50–95%. The structural determination of compound **6** was supported by ^1^H and ^13^C nuclear magnetic resonance (NMR) spectroscopy, high-resolution mass spectrometry, and single-crystal X-ray diffraction (see the later discussion).

**Scheme 2:**
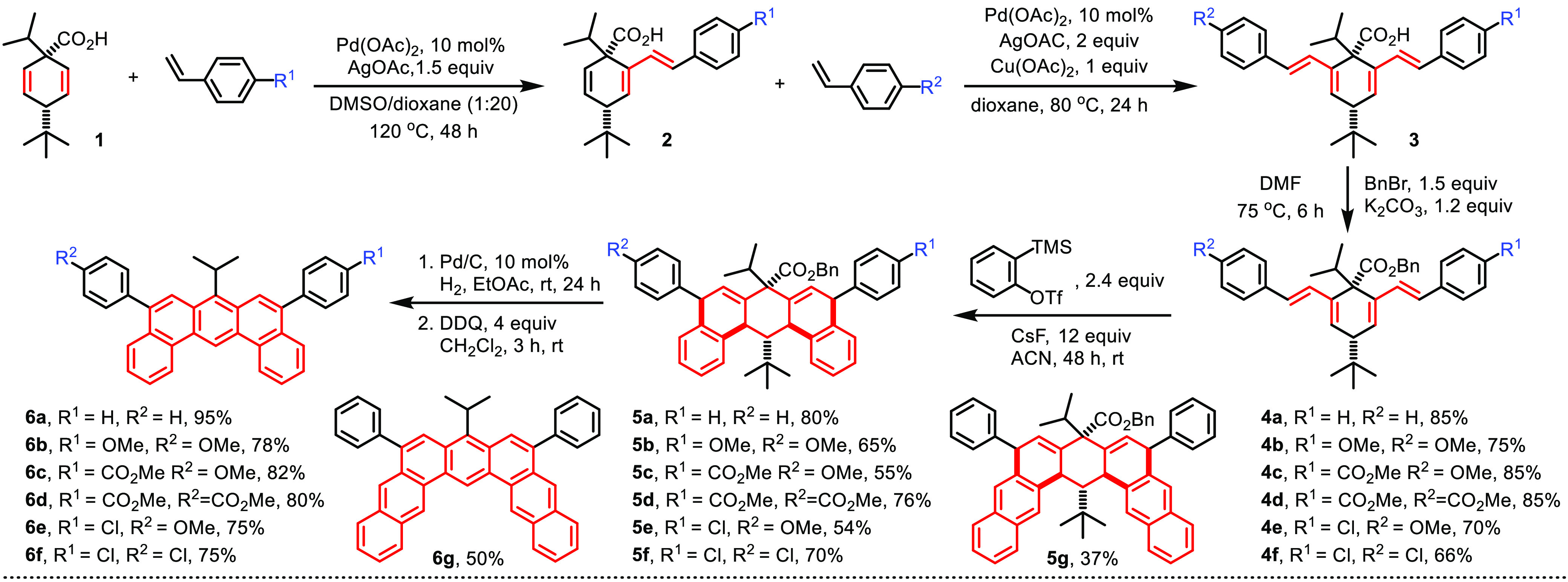
Synthetic Route for Diaryl Dibenzanthracenes and Diaryl Dinaphthoanthracene

Although bilateral 1,3-diene **3** can be, in principle, a suitable substrate for Diels–Alder reaction with aryne, we have reported that its carboxylic acid functionality would also react with aryne to give aryl ester (along with other side reactions).^16b,17^ To avoid the undesirable consumption of valuable aryne precursors, it was decided to convert compound **3** into benzyl ester **4**. The desired carboxylic acid functionality could later be liberated by hydrogenation prior to oxidative aromatization. Methyl esters had been considered an alternative to benzyl esters **4**; however, hydrolysis of the methyl ester analogue of compound **5a** was unsuccessful (compound **5aa**; see the Supporting Information).

It is unanticipated that we observed the elimination of the *t*-butyl group instead of a simple hydride in the final oxidative aromatization of compound **5**. Such a process is likely to occur through the mechanism shown in Scheme 3. In the first two steps i and ii, dehydrogenative oxidation in the “angled” rings proceeds through the sequential removal of hydride and protons by DDQ.^18^ Finally, hydride transfer from the *t*-butyl group to (protonated) quinone is then driven by the elimination of CO_2_ and isobutene and the relief of steric congestion around the *t*-butyl group to give aromatized dibenz[*a*,*j*]anthracenes.

**Scheme 3:**
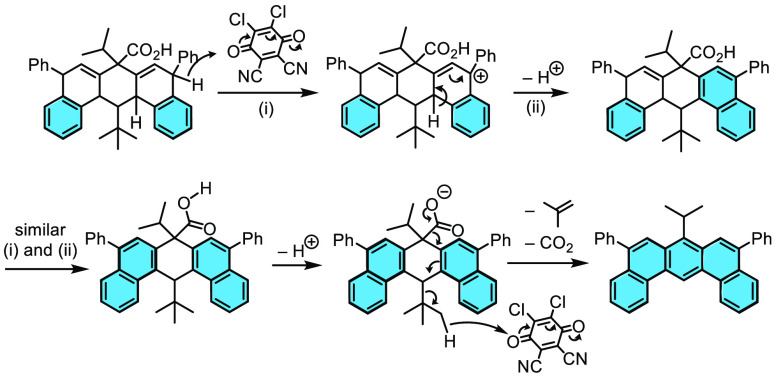
Proposed Mechanism for Oxidative and Decarboxylative Aromatization by DDQ

The structure of DBA **6a** is confirmed by single-crystal X-ray diffraction analysis, which shows five consecutive fused rings in a nearly planar geometry (Figure 1). To the best of our knowledge, this is the first DBA crystal structure without substituents in the “gulf” region (14 position).^15b,19^ Edge-to-face C–H···π (C···C = 3.55 Å between the Ph substituent and the terminal benzo ring) and π···π (3.72 Å between the mean planes of neighboring molecules) contacts are likely responsible for the observed dimer pairs in the solid. A slight non-planarity of 17° between the two planes defined by two terminal benzene units of DBA was observed. We believe such a torsion results from the asymmetric intermolecular packing features described above but not intrinsic to the DBA skeleton, supported by the planar geometry found in the density functional theory (DFT)-optimized structure at the level of ωB97X-D/6-31G(d,p) (Gaussian 09; see the Supporting Information for the full citation). No extended π stack was found, which is consistent with the excellent solubility of compound **6** in common organic solvents. As anticipated, the “angled” rings in compound **6a** display noticeable C–C/C=C bond length alternation and are less aromatic; the C_5_–C_6_ and C_8_–C_9_ bonds are about 1.36 Å, typical of a formally C=C double bond (“d” bonds in Figure 1a), whereas three other bonds in these rings are about 1.45 Å (“s” bonds), in accordance with Clar’s aromatic sextet rule.^20^

**Figure 1:**
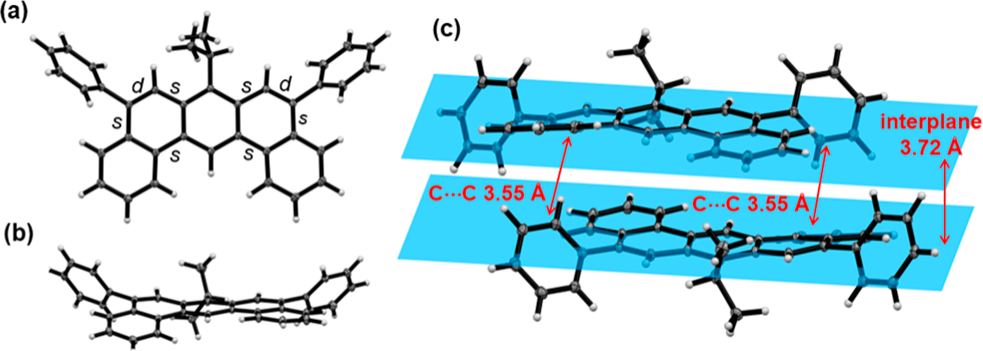
X-ray crystal structure of compound **6a** (thermal ellipsoids are shown at 50% probability): (a) top view of compound **6a**, (b) side view of compound **6a** highlighting the non-planarity, and (c) close edge-to-face C–H···π and π···π contacts between neighboring molecules. C–C bond distances are in the range of 1.44–1.46 Å for the s type and 1.36 Å for the d type.

These nonlinear DBAs are colorless in solutions, unlike purple-colored pentacene (absorption λ_max_ ∼ 600 nm in solutions), an isomeric PAH with five linearly fused benzene rings. Variations of the electronic nature of the substituents on the 5- and 9-phenyl substituents of DBA **6a**–**6f** caused a negligible effect on their photophysical properties (Table 1). The onsets of UV absorption are around 380 nm with the absorption maxima λ_max_ ∼ 320 nm measured in ethyl acetate, and blue emissions were found in λ_em_ of 420–426 nm for compounds **6a**–**6f** (Figure 2). Naphtho-fused compound **6g**, on the other hand, shows a noticeable red shift in absorption and emission with its large π-conjugated aromatic core. It should be noted that the apparent large Stoke shift between λ_max_ and λ_em_ is due to the very weak S_0_ → S_1_ transition (around the absorption onset at 380 nm) compared to the higher energy transitions. While S_0_ → S_2_ has a dominating highest occupied molecular orbital (HOMO) → least unoccupied molecular orbital (LUMO) character (DFT-computed oscillator strength *f* of 0.02–0.03; Table S1 of the Supporting Information), S_0_ → S_1_ and S_0_ → S_3_ feature anti- and in-phase configuration interactions of HOMO – 1 → LUMO and HOMO → LUMO + 1, respectively, resulting in nearly forbidden S_1_ (*f* ∼ 0.000–0.005) but strong S_3_ (*f* ∼ 1.37–2.35) transitions, commonly observed for alternant aromatic hydrocarbons of small-to-medium size.^21^ Although electron donor–acceptor substitution (e.g., compounds **6c** and **6e**) allows for some mixing of the three transition configurations mentioned above (see section 6 of the Supporting Information), it was not found to be sufficient to alter the nature of the S_1_ state and increase its oscillator strength. Despite the “dark” nature of S_1_, these molecules show highly structured emission profiles with moderate photoluminescence quantum yields (Φ_PL_ of 5–12%), corroborating a rigid molecular structure to avoid non-radiative vibrational relaxation.

**Table 1:** Optical and Electrochemical Properties of Compounds **6a**–**6g**

	λ_max_^a^ (nm) [ε (M^–1^ cm^–1^)]	Φ_PL_^b^ (%)	λ_em_^a^ (nm)	*E*_ox,onest_^c^ (V)
**6a**	315 [22000]	7.80	420, 440	0.96
**6b**	318 [33000]	9.10	421, 442	0.82
**6c**	322 [30000]	11.50	426, 446	0.78
**6d**	319 [32000]	9.50	424, 444	0.89
**6e**	317 [28000]	9.30	423, 442	0.75
**6f**	316 [33000]	5.30	421, 439	0.85
**6g**	350 [35000]	6.50	451, 479	0.83

a Measured in EtOAc.

b Absolute photoluminescence quantum efficiency, measured using an integrating sphere.

c Measured in a CH_2_Cl_2_ solution containing 0.1 M *n*Bu_4_NClO_4_ (V versus Ag/Ag^+^).

**Figure 2:**
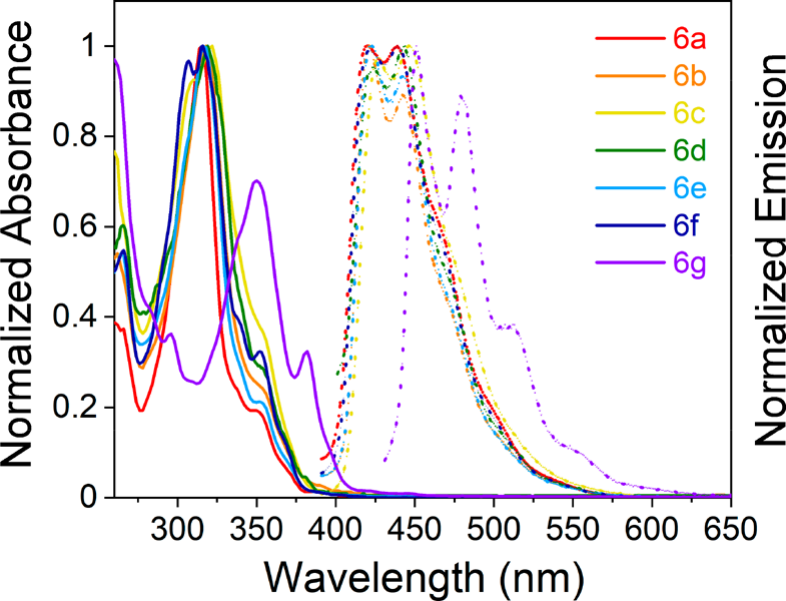
Normalized absorption (solid lines) and emission (dashed lines) of compounds **6a**–**6g** were recorded in ethyl acetate.

The insensitivity of the optical transition energy to the nature of 5,9 substituents of compounds **6a**–**6f** is usually considered evidence of the parallel shifting of the HOMO and LUMO energy. The cyclic voltammograms of the compounds are depicted in Figure S1 of the Supporting Information. The onset potential of the electrochemical oxidation, *E*_ox,onest_, was estimated and is listed in Table 1. The oxidation reactions were found to be irreversible. The absence of cathodic current responses with a magnitude comparable to that of the anodic current responses raises uncertainty regarding the reliable estimation of the LUMO of the compounds. Furthermore, the estimation of the LUMO level by optical absorption is complicated by the non-HOMO-to-LUMO character of the S_1_ state. However, we note that the invariance in the excitation energy for compounds **6a**–**6f** is well-reproduced by time-dependent density functional theory (TD-DFT) calculations (Table S1 of the Supporting Information). Additionally, a good correlation was found between the computed HOMO and LUMO energy with the average Hammett substituent constants σ_p_, instead of the Hammett constant of the more electron-rich (for HOMO) or electron-poor (for LUMO) substituent (Figures S2 and S3 of the Supporting Information). Such a correlation indicates that the substituents on the 5,9-diphenyl groups in compounds **6a**–**6f** only influence the electron structure of DBA in a perturbative way but do not dominate the overall properties of the molecule. This observation is in line with the leading contribution of the DBA core motif to the molecular frontier orbitals in comparison to that of the 5,9-diphenyl groups (Figure 3).

**Figure 3:**
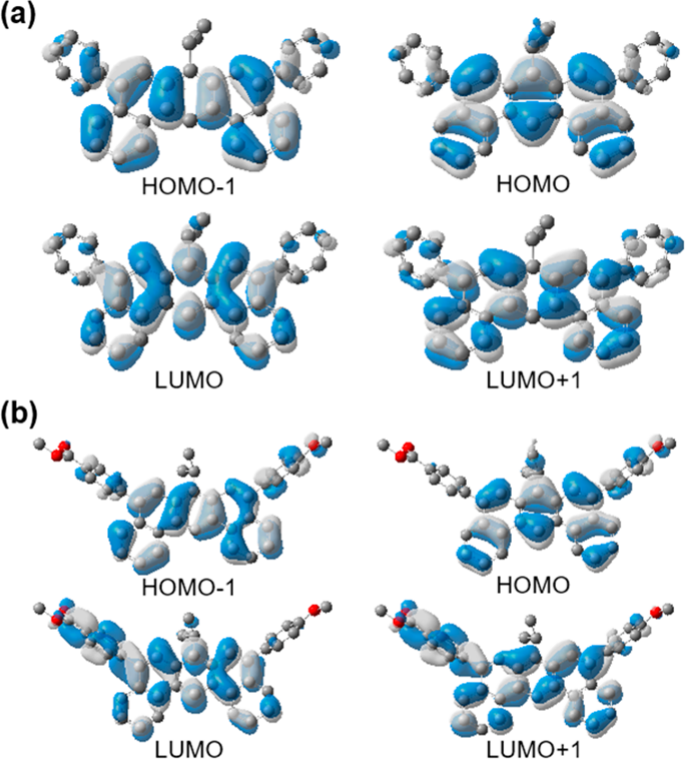
Visualization of frontier orbitals of compounds (a) **6a** and (b) **6c**, representative of symmetrically and asymmetrically substituted DBAs, respectively.

In conclusion, the developed synthetic approach enables the regiospecific synthesis of DBA derivatives with controlled functionalization. The key step of the synthesis involves the Diels–Alder reaction with proaromatic butadiene precursors, while the subsequent oxidation exhibits an unusual elimination of the *t*-butyl group during decarboxylative aromatization. Although the 5,9-diphenyl substituents do not have a significant impact on the optical properties of the synthesized compounds, our analysis provided valuable insights into this observation. Building upon these findings, our ongoing research aims to explore the full potential of the DBA scaffold by introducing heterocycles and five-membered ring motifs. This exploration seeks to enhance the optical absorption properties of DBA derivatives in terms of visible-light absorptivity and emission intensity.

## Data Availability

The data underlying this study are available in the published article and its Supporting Information.

## Supporting Information Available

Experimental procedures, characterization data, NMR spectra, crystallographic data, and electrochemical and DFT analyses (PDF)
