# Supplementary material for: Butterfly-Shaped Dibenz[a,j]anthracenes: Synthesis and Photophysical Properties
Source: Org Lett. 2023 Aug 25;25(43):7763–8. doi: 10.1021/acs.orglett.3c02306 (PMC10630963; doi:10.1021/acs.orglett.3c02306)
Supplement: Supplementary file 1 — ol3c02306_si_001.pdf [file ol3c02306_si_001.pdf]

Supporting Information

Butterfly-Shaped Dibenz[*a,j*]anthracenes: Synthesis, and Photophysical Properties

Yan-Ying Wu,[a] Yi-Lin Wu,*[,b] Cheng-Lan Lin,*[,c] Hung-Cheng Chen,[a] Yao-Yuan Chuang,[a] Chih-Hsien Chen,*[,d] and Chih-Ming Chou*[,a]

[a] Department of Applied Chemistry, National University of Kaohsiung, Kaohsiung 81148, Taiwan

Email: cmchou@nuk.edu.tw

[b] School of Chemistry, Cardiff University, Main Building, Park Place, Cardiff CF10 3AT, United Kingdom

Email: WuYL@cardiff.ac.uk

[c] Department of Chemical and Materials Engineering, Tamkang University, New Taipei City, Taiwan

[d] Department of Chemical Engineering, Feng Chia University, Taichung 407, Taiwan

Table of Contents

1. General information....................S2
2. General synthetic procedure....................S3
3. Analytical data....................S6
4. Single crystal data of **6a**....................S18
5. Electrochemical analysis....................S19
6. TD-DFT excitation analysis....................S20
7. Cartesian coordinates for optimized geometries....................S27
8. ^{1}H and ^{13}C NMR Spectra....................S34
9. References....................S55

1. General information

All solvents and reagents were purified according to standard procedures or were used as received from Aldrich, Fluka, Acros or Lancaster. (^{1}H and ^{13}C NMR) spectra were recorded on a Varian-Mercury-300 (300 MHz) spectrometer. Chemical shifts for protons are reported in parts per million (ppm) downfield from TMS and are referenced to the residual proton in the NMR solvent ($CDCl_3$ δ = 7.26 ppm). ^{13}C chemical shifts are reported in ppm downfield from TMS and were referenced to the carbon resonances of the solvent ($CDCl_3$ δ = 77.0 ppm). NMR data are indicated as follows: chemical shift, multiplicity (br = broad, s = singlet, d = doublet, t = triplet, q = quartet, m = multiplet), coupling constants in Hertz (Hz), and integration. TLC was performed using Merck silica gel 60 F-254 plates, detection of compounds with UV light or dipping into a solution of $KMnO_4$ followed by heating. Flash column chromatography was performed using Merck silica gel 60 (40-63 μm), applying a pressure of about 0.4 bar. Melting point was measured using a Fargo MP-2D apparatus. The electron impact (EI) mass spectral data were obtained using a SHIMADZU QP2020 and JEOL AccuTOF GCx-plus. The single crystal X-ray diffraction data were obtained using a Bruker D8 VENTURE with IμS 3.0 Dual Wavelength system. Cyclic voltammetry (CV) of the compounds was conducted using a conventional three-electrode electrochemical system. A CHI potentiostat/galvanostat electrochemical analyzer CHI6273D was employed for the CV experiments. The working electrode was a platinum disk electrode with a diameter of 2 mm. An Ag/Ag^{+} electrode and a platinum coil were used as the reference and counter electrodes, respectively. The electrolyte was a tetrahydrofuran (THF) solution containing 0.1 M tetrabutylammonium perchlorate. The scan rate was 50 mV/s. Steady-state absorption spectra were measured by Agilent Cary 60 UV-Vis spectrophotometer. Steady-state fluroescence spectra and absolute quantum yield were measured by FS5 Spectrofluorometer and integrating sphere (Edinburgh Instruments Ltd.)

2. General synthetic procedure

General procedure Ⅰ: Palladium-catalyezd C-H olefination[1]

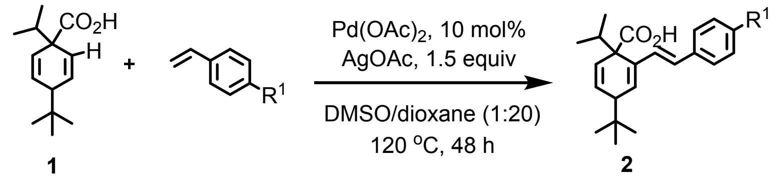

1 **2**

A screw-cap Schlenk tube was charged with proaromatic acid **1**[2] (1.2 equiv.), AgOAc (1.5 equiv), $Pd(OAc)_2$ (10 mol%), dry DMSO/dioxane (1:20) followed by appropriate styrene (1 equiv). The mixture was kept for stirring in a preheated oil bath at 120 °C for 48 h. The reaction mixture was then extracted with ethyl acetate, washed with 10% $NaHCO_{3(aq)}$ solution and brine. The organic extract was dried over $MgSO_4$, filtered and concentrated under vacuum. The crude mixture was purified by flash column chromatography (SiO_2) to afford the desired product **2**.

General procedure Ⅱ: Palladium-catalyezd C-H olefination

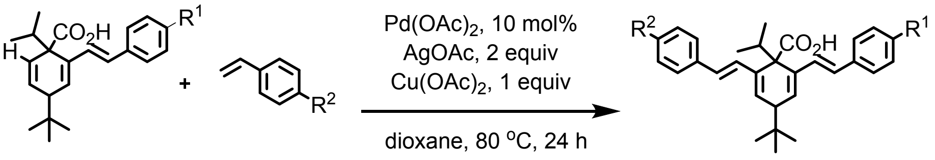

2 **3**

To a screw-cap Schlenk tube, mono-olefinated proaromatic acid **2** (1 equiv), AgOAc (2 equiv), $Cu(OAc)_2$ (1 equiv), $Pd(OAc)_2$ (10 mol%), dry dioxane followed by appropriate styrene (2 equiv) were added. The mixture was stirred in a preheated oil bath at 80 °C for 24 h. The mixture was then extracted with ethyl acetate, washed with 10% HCl and brine. The organic extract was dried over $MgSO_4$, filtered and concentrated under vacuum. The crude residue was purified by flash column chromatography (SiO_2) to afford the desired product **3**.

➢ Compounds **1**, **2**, and **3** were prepared according to references 1 and 2. The resulting ^1H NMR are identical with the literature's report.

General procedure Ⅲ: Benzylation

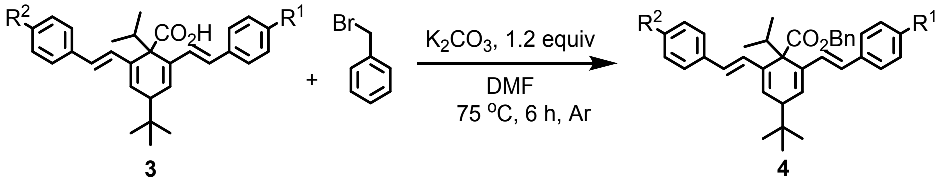

3 **4**

Benzyl bromide (1.5 equiv) was added into a solution containing K_2CO_3 (1.2 equiv) and compound **3** in *N*,*N*-dimethylformamide (DMF, 0.04 M) under Ar at room temperature. The solution was heated at 75 °C for 6 h and then cooled to room temperature by an ice-water bath. Water was added to the reaction solution; the mixture was extracted with ethyl acetate. The organic phase was washed with 10% $NaHCO_{3(aq)}$, saturated $NaCl_{(aq)}$, dried over $MgSO_4$, and concentrated under vacuum. The residue was purified by flash column chromatography (SiO_2) to afford the product **4**.

General procedure Ⅳ: Diels–Alder reaction

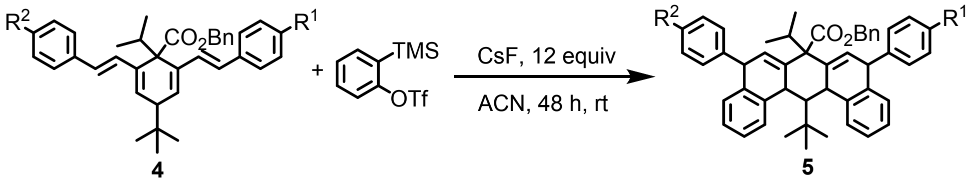

4 **5**

A screw-cap schlenk tube with the compound **4** (1 equiv), Caesium fluoride (12 equiv), Acetonitrile (0.1 M) and 2-(trimethylsilyl)phenyl trifluoromethanesulfonate (2.4 equiv) were added. The resulting mixture was stirred at room temperature under Ar gas for 48 h. The reaction mixture was extracted with ethyl acetate and filtered through a short pad of silica gel and eluted with ethyl acetate. The solvent was removed under reduced pressure and the crude mixture was purified by flash column chromatography (SiO_2) to afford the desired product **5**.

General procedure V: for the decarboxylative aromatization reaction

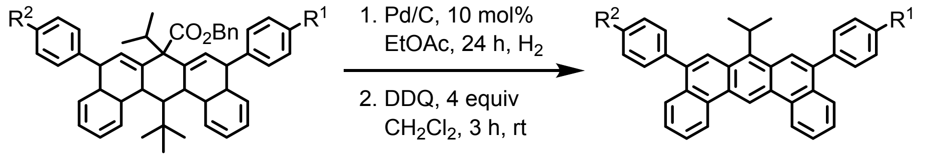

5 **6**

Step 1: The compound **5** (1 equiv) was added into screw-cap schlenk tube with Pd/C (10 mol%) in ethyl acetate under hydrogen gas at room temperature. The resulting mixture was stirred at room temperature for 24 h. hen extracted with ethyl acetate (0.05 M). Dried over $MgSO_4$ and filtered. The solvent was removed under vacuo to get the hydrogenative compounds. Step 2: To a hydrogenative compound in dichloromethane (0.05 M), DDQ (4 equiv) was added and stirred at room temperature under Ar gas for 3 h. The mixture was then extracted with ethyl acetate and washed twice with water, dried over $MgSO_4$ and filtered. The solvent was removed under vacuo and the crude residue was purified by flash column chromatography (SiO_2) to afford the desired product compound **6**.

3. Analytical data

Benzyl 4-(*tert*-butyl)-1-isopropyl-2,6-di((*E*)-styryl)cyclohexa-2,5-diene-1-carboxylate (4a)

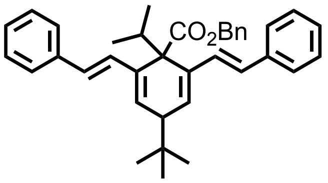

4a

According to general procedure Ⅲ: **3a** (0.2 mmol, 96.5 mg), benzyl bromide (0.3 mmol, 51.3 mg), K_2CO_3 (0.24 mmol, 33.2 mg) and dimethylformamide (5 mL) at 75 °C for 6 h. The crude product was purification by flash column chromatography (Hexane/Ether) Provide **4a** as a yellow liquid (87.8 mg, 85%): **^{1}H NMR** (300 MHz, $CDCl_3$) δ 7.40-7.16 (m, 15H), 6.67 (d, *J* = 15 Hz, 2H), 6.54 (d, *J* = 15 Hz, 2H), 6.37 (s, 2H), 5.15 (s, 2H), 2.63 (br, 1H), 2.58-2.49 (m, 1H), 1.02 (s, 9H), 0.91 (d, *J* = 6 Hz, 6H). **^{13}C{^{1}H} NMR** (75 MHz, $CDCl_3$) δ 173.4, 159.0, 135.6, 130.4, 128.4, 127.9, 127.6, 127.3, 125.4, 113.9, 66.5, 57.7, 55.3, 27.9, 18.9. **HRMS** (EI) m/z: $[M]^+$ calcd for $C_{37}H_{40}O_2$: 516.3028. Found: 516.3024.

Benzyl 4-(*tert*-butyl)-1-isopropyl-2,6-bis((*E*)-4-methoxystyryl)cyclohexa-2,5-diene-1-carboxylate (4b)

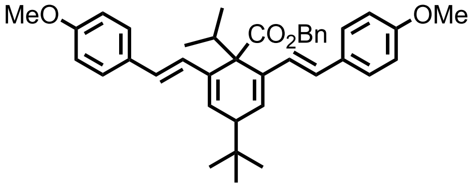

4b

According to general procedure Ⅲ: **3b** (0.2 mmol, 97.3 mg), benzyl bromide (0.3 mmol, 51.3 mg), K_2CO_3 (0.24 mmol, 33.2 mg) and dimethylformamide (5 mL) at 75 °C for 6 h. The crude product was purification by flash column chromatography (Hexane/Ether) Provide **4b** as a yellow liquid (86.5 mg, 75%): **^{1}H NMR** (300 MHz, $CDCl_3$) δ 7.47 -7.25 (m, 9H), 6.90 (d, *J* = 6 Hz, 4H), 6.73 (d, *J* = 15 Hz, 2H), 6.56-6.33 (m, 4H), 5.23 (s, 2H), 3.84 (s, 6H), 2.70 (br, 1H), 2.63 (m, 1H), 1.10 (s, 9H), 1.02 (d, *J* = 6 Hz, 6H). **^{13}C{^{1}H} NMR** (75 MHz, $CDCl_3$) δ 172.9,

166.9, 141.9, 135.7, 135.3, 132.0, 128.6, 128.2, 127.3, 126.9, 126.2 , 66.8, 57.5, 52.0, 47.3, 35.4, 34.1, 27.9, 18.8. **HRMS** (EI) m/z: $[M]^+$ calcd for $C_{39}H_{44}O_4$: 576.3240. Found: 576.3241.

4-(tert-butyl)-1-isopropyl-2-((E)-4-(methoxycarbonyl)styryl)-6-((E)-4-methoxystyryl)cyclohexa-2,5-diene-1-carboxylic acid (4c)

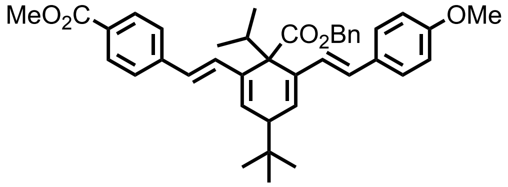

4c

According to general procedure Ⅲ: **3c** (0.2 mmol, 151.2 mg), benzyl bromide (0.3 mmol, 51.3 mg), K_2CO_3 (0.24 mmol, 33.2 mg) and dimethylformamide (5 mL) at 75 °C for 6 h. The crude product was purification by flash column chromatography (Hexane/Ether) Provide **4c** as a yellow liquid (87.8 mg, 85%): **^{1}H NMR** (300 MHz, $CDCl_3$) δ 7.99 (d, *J* = 9 Hz, 2H), 7.42-7.19 (m, 9H), 6.86 (t, *J* = 9 Hz, 2H), 6.68 (d, *J* = 9 Hz, 3H), 6.50-6.41 (m, 2H), 6.37 (s, 1H), 5.19 (q, *J* = 12 Hz, 2H), 3.93 (s, 3H), 3.82 (s, 3H), 2.68 (br, 1H), 2.57-2.53 (m, 1H), 1.07 (s, 9H), 0.96 (d, *J* = 6 Hz, 6H). **^{13}C{^{1}H} NMR** (75 MHz, $CDCl_3$) δ 173.2, 166.9, 159.1, 142.1, 135.9, 135.3, 132.4, 130.3, 129.9, 128.6, 128.5, 128.4, 128.1, 127.2, 126.7, 126.2, 125.1, 114.0, 66.7, 57.6, 55.3, 52.0, 47.3, 35.4, 34.1, 27.9, 18.8. **HRMS** (EI) m/z: $[M]^+$ calcd for $C_{40}H_{44}O_5$: 604.3189. Found: 604.3184.

Dimethyl-l,4'-((1E,1'E)-(2-((benzyloxy)carbonyl)-5-(tert-butyl)-2-isopropylcyclohexa-3,6-diene-1,3-diyl)bis(ethene-2,1-diyl))dibenzoate (4d)

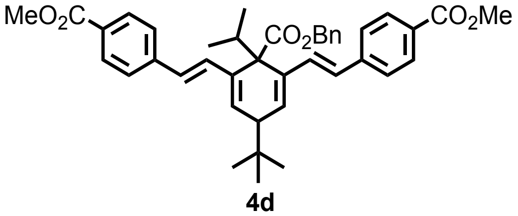

4d

According to general procedure Ⅲ: **3d** (0.2 mmol, 108.5 mg), benzyl bromide (0.3 mmol, 51.3 mg), K_2CO_3 (0.24 mmol, 33.2 mg) and dimethylformamide (5 mL) at 75 °C for 6 h. The crude product was purification by flash column chromatography (Hexane/Ether) Provide **4d** as

yellow solid; m.p.; 146-147 ℃ (107.6 mg, 85%): ^{1}**H NMR** (300 MHz, $CDCl_3$) δ 7.96 (d, *J* = 6 Hz, 4H), 7.38-7.28 (m, 6H), 7.25 (d, *J* = 6 Hz, 3H), 6.74-6.60 (m, 4H), 6.42 (s, 2H), 5.17 (s, 2H), 3.91 (s, 6H), 2.67 (br, 1H), 2.58-2.47 (m, 1H), 1.04 (s, 9H), 0.92 (d, *J* = 6 Hz, 6H). ^{13}**C{^{1}H} NMR** (75 MHz, $CDCl_3$) δ 173.0, 166.9, 141.9, 135.7, 135.3, 132.0, 128.6, 128.2, 127.3, 126.9, 126.2 , 66.8, 57.5, 52.0, 47.3, 35.4, 34.1, 27.9, 18.8. **HRMS** (EI) m/z: $[M]^+$ calcd for $C_{41}H_{44}O_6$: 632.3138. Found: 632.3134.

Benzyl-4-(tert-butyl)-2-((E)-4-chlorostyryl)-1-isopropyl-6-((E)-4-methoxystyryl)cyclohexa-2,5- diene-1-carboxylate (4e)

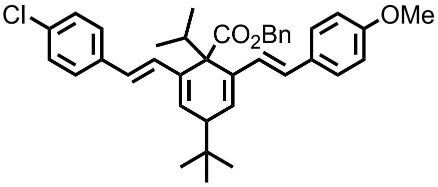

4e

According to general procedure Ⅲ: **3e** (0.2 mmol, 98.2 mg), benzyl bromide (0.3 mmol, 51.3 mg), K_2CO_3 (0.24 mmol, 33.2 mg) and dimethylformamide (5 mL) at 75 ^{o}C for 6 h. The crude product was purification by flash column chromatography (Hexane/Ether) Provide **4e** as a yellow liquid (81.5 mg, 70%): ^{1}**H NMR** (300 MHz, $CDCl_3$) δ 7.48-7.26 (m, 11H), 6.87 (d, *J* = 9 Hz, 2H), 6.72-6.62 (m, 2H), 6.60-6.35 (m, 4H), 5.25-5.14 (m, 2H), 3.85 (s, 3H), 2.66 (br, 1H), 2.58-2.52 (m, 1H), 1.07 (s, 9H), 0.95 (d, *J* = 6 Hz, 6H). ^{13}**C{^{1}H} NMR** (75 MHz, $CDCl_3$) δ 173.3, 159.1, 136.1, 136.0, 135.7, 135.4, 132.8, 130.4, 128.7, 128.5, 128.5, 128.1, 127.7, 127.6, 127.6, 127.4, 126.5, 114.0, 66.6, 57.7, 55.3, 47.3, 35.4, 34.1, 27.9, 18.9. **HRMS** (EI) m/z: $[M]^+$ calcd for $C_{38}H_{41}ClO_3$: 580.2744. Found: 580.2740.

Benzyl-4-(tert-butyl)-2,6-bis((E)-4-chlorostyryl)-1-isopropylcyclohexa-2,5-diene-1-carboxylate (4f)

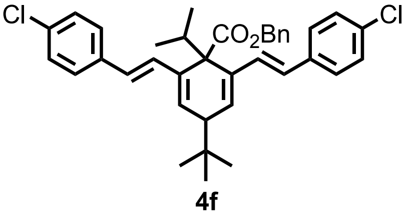

4f

According to general procedure Ⅲ: **3f** (0.2 mmol, 98.84 mg), benzyl bromide (0.3 mmol, 51.3 mg), K_2CO_3 (0.24 mmol, 33.2 mg) and dimethylformamide (5 mL) at 75 °C for 6 h. The crude product was purification by flash column chromatography (Hexane/Ether) Provide **4f** : as a yellow liquid (77.1 mg, 66%): **^{1}H NMR** (300 MHz, $CDCl_3$) δ 7.36 – 7.11 (m, 13H), 6.61 (d, J = 15 Hz, 2H), 6.47 (d, J = 15 Hz, 2H), 6.35 (s, 2H), 5.15 (s, 2H), 2.63 (br, 1H), 2.49 (m, 1H), 1.02 (s, 9H), 0.89 (d, J = 6 Hz, 6H). **^{13}C{^{1}H} NMR** (75 MHz, $CDCl_3$) δ 173.1, 136.7, 136.0, 135.9, 135.3, 132.8, 130.1, 128.7, 128.6, 128.5, 128.1, 127.6, 126.7, 126.5, 66.7, 57.6, 47.3, 35.4, 34.1, 27.9, 18.8. **HRMS** (EI) m/z: $[M+H]^+$ calcd for $C_{37}H_{38}Cl_2O_2$: 585.2327. Found: 585.2321.

Benzyl-14-(tert-butyl)-7-isopropyl-5,9-diphenyl-4a,5,7,9,9a,13a,13b,14,14a,14b-decahydrobenzo-[m]tetraphene-7-carboxylate (5a)

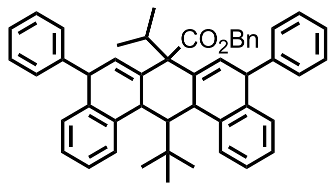

5a

According to general procedure Ⅳ: **4a** (0.25 mmol, 168.2 mg), caesium fluoride (3 mmol, 455.7 mg), 2-(Trimethylsilyl)phenyl trifluoromethanesulfonat (0.6 mmol, 178.8 mg) and acetonitrile (2.5 mL) at rt for 48 h. The crude product purification by flash column chromatography (Hexane/EA) Provide **5a** as a white solid; m.p.: 185-186 ℃ (125.6 mg, 80%): **^{1}H NMR** (300 MHz, $CDCl_3$) δ 7.62-6.92 (m, 23H), 6.53 (d, J = 3 Hz, 2H), 5.09 (s, 2H), 4.61 (s, 2H), 3.28 (d, J = 9 Hz, 2H), 2.61 (m, 1H), 2.24 (t, J = 12 Hz, 1H), 0.79 (s, 6H), 0.59 (s, 9H). **^{13}C{^{1}H} NMR** (75 MHz, $CDCl_3$) δ 174.2, 143.8, 143.2, 142.6, 135.9, 129.1, 128.5, 128.4, 128.2, 127.9, 126.4, 125.0, 124.9, 124.8, 66.6, 62.9, 48.9, 48.2, 45.9, 35.9, 28.9, 19.3. **HRMS** (EI) m/z: $[M]^+$ calcd for $C_{49}H_{48}O_2$: 668.3654. Found 668.3648.

Methyl-14-(tert-butyl)-7-isopropyl-5,9-diphenyl-5,7,9,13b,14,14a-hexahydrobenzo[m]tetraphene-7-carboxylate (5aa)

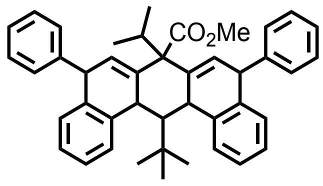

5aa

According to general procedure **Ⅳ**: methyl 4-(tert-butyl)-1-isopropyl-2,6-di-((E)-styryl)cyclohexa-2,5-diene-1-carboxylate (0.25 mmol, 160.2 mg), caesium fluoride (3 mmol, 455.7 mg), 2-(Trimethylsilyl)phenyl trifluoromethanesulfonat (0.6 mmol, 178.8 mg) and acetonitrile (2.5 mL) at rt for 48 h. The crude product purification by flash column chromatography (Hexane/EA) Provide **5aa** as a white solid; m.p.: 188-189 ℃ (120.6 mg, 80%): **^{1}H NMR** (300 MHz, $CDCl_3$) δ 7.54 – 7.27 (m, 10H), 7.18-6.80 (m, 8H), 6.51 (d, J = 6 Hz, 2H), 4.59 (s, 2H), 3.62 (s, 3H), 3.30 (d, J = 9 Hz, 2H), 2.57 (m, 1H), 2.24 (t, J = 9 Hz, 1H), 0.80 (s, 6H), 0.61 (s, 9H). **^{13}C{^{1}H} NMR** (75 MHz, $CDCl_3$) δ 174.9, 144.1, 143.2, 142.7, 129.1, 128.5, 127.5, 126.4, 124.8, 62.9, 52.3, 48.9, 47.9, 46.1, 35.9, 28.9, 19.2.**HRMS** (EI) m/z: $[M]^+$ calcd for $C_{43}H_{44}O_2$: 592.3341. Found 592.3348.

Benzyl-14-(tert-butyl)-7-isopropyl-5,9-bis(4-methoxyphenyl)-4a,5,7,9,9a,13a,13b,14,14a,14b-decahydrobenzo[m]tetraphene-7-carboxylate (5b)

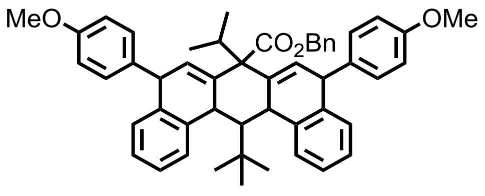

5b

According to general procedure **Ⅳ**: **4b** (0.25 mmol, 183.2 mg), caesium fluoride (3 mmol, 455.7 mg), 2-(Trimethylsilyl)phenyl trifluoromethanesulfonat (0.6 mmol, 178.8 mg) and acetonitrile (2.5 mL) at rt for 48 h. The crude product purification by flash column chromatography (Hexane/EA) Provide **5b** as a white solid; m.p.; 173-174 ℃ (107.2 mg, 65%): **^{1}H NMR** (300 MHz, $CDCl_3$) δ 7.26-7.01 (m, 17H), 6.93 (d, J = 9 Hz, 4H), 6.46 (s, 2H), 5.07 (s, 2H), 4.52 (s, 2H), 3.87 (s, 6H), 3.24 (s, 2H), 2.60 (m, 1H), 2.26 (m, 1H), 0.77 (s, 6H), 0.57 (s, 9H). **^{13}C{^{1}H} NMR** (75 MHz, $CDCl_3$) δ 158.2, 143.5, 142.6, 135.9, 135.3, 128.2, 125.2, 113.8, 66.6, 62.9, 55.4, 48.9, 45.9, 35.9, 28.9. **HRMS** (EI) m/z: $[M]^+$ calcd for $C_{51}H_{52}O_4$: 728.3866. Found: 728.3858.

Benzyl-14-(tert-butyl)-7-isopropyl-5-(4-(methoxycarbonyl)phenyl)-9-(4-methoxyphenyl)-4a,5,7,9,9a,13a,13b,14,14a,14b-decahydrobenzo[m]tetraphene-7-carboxylate (5c)

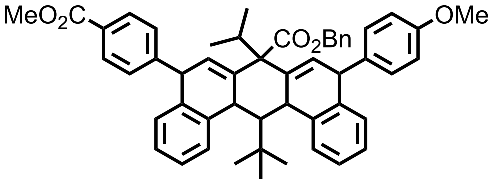

5c

According to general procedure **Ⅳ**: **4c** (0.25 mmol, 151.2 mg), caesium fluoride (3 mmol, 455.7 mg), 2-(Trimethylsilyl)phenyl trifluoromethanesulfonat (0.6 mmol, 178.8 mg) and acetonitrile (2.5 mL) at rt for 48 h. The crude product purification by flash column chromatography (Hexane/EA) Provide **5c** as a white solid; m.p.; 169-170 ℃ (104.6 mg, 55%): **^{1}H NMR** (300 MHz, $CDCl_3$) δ 8.10 (d, *J* = 6 Hz, 2H), 7.42 (d, *J* = 9 Hz, 2H), 7.3-6.75 (m, 17H), 6.52 (t, *J* = 6 Hz, 2H), 5.11 (s, 2H), 4.60 (br, 2H), 3.97 (s, 3H),3.89 (s, 3H), 3.28 (t, *J* = 9 Hz, 2H), 2.62 (m, 1H), 2.21 (t, *J* = 9 Hz, 1H), 0.81 (s, 6H), 0.60 (s, 9H). **^{13}C{^{1}H} NMR** (75 MHz, $CDCl_3$) δ 174.1, 167.1, 158.3, 148.8, 144.5, 142.5, 135.9, 135.2, 129.8, 129.0, 128.4, 128.2, 128.0, 124.6, 113.9, 66.7, 63.0, 55.4, 52.2, 49.1, 47.7, 46.0, 45.8, 36.0, 28.9, 19.4. **HRMS** (EI) m/z: $[M]^+$ calcd for $C_{52}H_{52}O_5$: 756.3815. Found: 756.3809.

Dimethyl-4,4'-(7-((benzyloxy)carbonyl)-14-(tert-butyl)-7-isopropyl-4a,5,7,9,9a,13a,13b,14,14a,14b-decahydrobenzo[m]tetraphene-5,9-diyl)dibenzoate (5d)

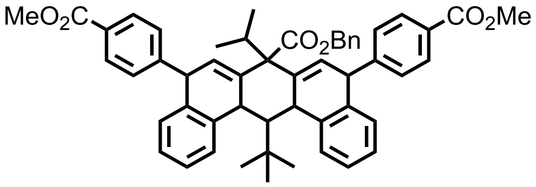

5d

According to general procedure **Ⅳ**: **4d** (0.25 mmol, 183.2 mg), caesium fluoride (3 mmol, 455.7 mg), 2-(Trimethylsilyl)phenyl trifluoromethanesulfonat (0.6 mmol, 178.8 mg) and acetonitrile (2.5 mL) at rt for 48 h. The crude product purification by flash column chromatography (Hexane/EA) Provide **5d** as a white solid; m.p.: 170-171 ℃ (149.9 mg, 76%):

^{1}H NMR (300 MHz, $CDCl_3$) δ 8.09 (d, J = 9 Hz, 4H), 7.94 (d, J = 9 Hz, 4H), 7.28-7.05 (m, 11H), 6.95 (br, 2H), 6.51 (d, J = 6 Hz, 2H), 5.09 (s, 2H), 4.63 (br, 2H), 3.97 (s, 6H), 3.27 (d, J = 9 Hz, 2H), 2.61 (m, 1H), 2.13 (m, 1H), 0.80 (s, 6H), 0.58 (s, 9H). **^{13}C{^{1}H} NMR** (75 MHz, $CDCl_3$) δ 174.0, 167.1, 148.7, 144.4, 142.4, 135.8, 129.9, 129.1, 128.2, 128.0, 125.1, 66.7, 62.9, 52.2, 49.2, 48.1, 45.9, 35.9, 28.9, 19.4. **HRMS** (EI) m/z: $[M]^+$ calcd for $C_{53}H_{52}O_6$: 784.3764. Found: 784.3758.

Benzyl-14-(tert-butyl)-5-(4-chlorophenyl)-7-isopropyl-9-(4-methoxyphenyl)-5,7,9,13b,14,14a-hexahydrobenzo[m]tetraphene-7-carboxylate(5e)

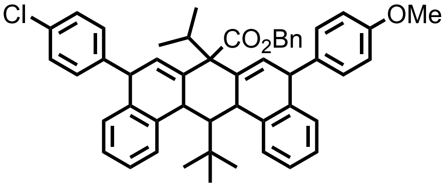

5e

According to general procedure **Ⅳ**: **4e** (0.25 mmol, 151.2 mg), caesium fluoride (3 mmol, 455.7 mg), 2-(Trimethylsilyl)phenyl trifluoromethanesulfonat (0.6 mmol, 178.8 mg) and acetonitrile (2.5 mL) at rt for 48 h. The crude product purification by flash column chromatography (Hexane/EA) Provide **5e**: as a white solid; m.p.: 170-171 ℃ (98.8 mg, 54%): **^{1}H NMR** (300 MHz, $CDCl_3$) δ 7.38 (d, J = 9 Hz, 2H), 7.28-6.79 (m, 19H), 6.54-6.48 (m, 2H), 5.10 (s, 2H), 4.53 (br, 2H), 3.88 (s, 3H), 3.26 (br, 2H), 2.67-2.51 (m, 1H), 2.24 (m, 1H), 0.80 (s, 6H), 0.60 (s, 9H). **^{13}C{^{1}H} NMR** (75 MHz, $CDCl_3$) δ 174.1, 158.2, 144.3, 143.3, 142.6, 141.9, 135.9, 135.2, 132.2, 130.3, 129.9, 128.6, 128.4, 128.2, 127.9, 124.9, 113.9, 66.7, 62.9, 55.4, 48.9, 47.1, 45.9, 35.9, 28.9, 19.3 **HRMS** (EI) m/z: $[M]^+$ calcd for $C_{50}H_{49}ClO_3$: 732.3363. Found: 732.3365.

Benzyl-14-(tert-butyl)-5,9-bis(4-chlorophenyl)-7-isopropyl-5,7,9,13b,14,14a-hexahydrobenzo[m]tetraphene-7-carboxylate (5f)

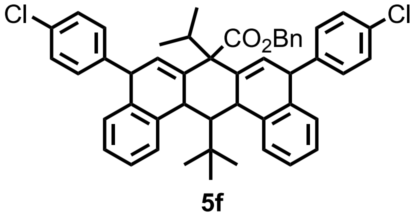

5f

According to general procedure **Ⅳ**: **4f** (0.25 mmol, 183.2 mg), caesium fluoride (3 mmol, 455.7 mg), 2-(Trimethylsilyl)phenyl trifluoromethanesulfonat (0.6 mmol, 178.8 mg) and acetonitrile (2.5 mL) at rt for 48 h. The crude product purification by flash column chromatography (Hexane/EA) Provide **5f** as a white solid ; m.p.:218-219 ℃ (128.9 mg, 70%): **^{1}H NMR** (300 MHz, $CDCl_3$) δ 7.38 (d, *J* = 9 Hz, 4H), 7.34-6.86 (m, 17H), 6.47 (d, *J* = 3 Hz, 2H), 5.10 (s, 2H), 4.55 (br, 2H), 3.25 (d, *J* = 9 Hz, 2H), 2.52-2.66 (m, 1H), 2.19 (t, *J* = 9 Hz, 1H), 0.78 (s, 6H), 0.60 (s, 9H). **^{13}C{^{1}H} NMR** (75 MHz, $CDCl_3$) δ 174.0, 144.0, 142.4, 141.8, 135.8, 132.3, 130.4, 128.6, 128.4, 128.3, 128.0, 125.2, 125.0, 66.7, 62.9, 49.0, 47.2, 45.8, 36.0, 28.9, 19.6. **HRMS** (EI) m/z: $[M]^+$ calcd for $C_{49}H_{46}Cl_2O_2$: 736.2875. Found: 736.2869.

Benzyl-17-(tert-butyl)-8-isopropyl-6,10-diphenyl-6,8,10,16b,17,17a-hexahydronaphtho[2,3-a]pentaphene-8-carboxylate (5g)

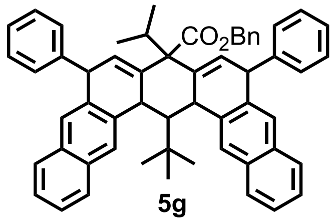

5g

According to general procedure **Ⅳ**: **4a** (0.25 mmol, 168.2 mg), caesium fluoride (3 mmol, 455.7 mg), 3-(trimethylsilyl)naphthalen-2-yl trifluoromethanesulfonate (0.6 mmol, 209.1 mg) and acetonitrile (2.5 mL) at rt for 48 h. The crude product purification by flash column chromatography (Hexane/EA) Provide **5g** as a orange solid; m.p.:190-191 ℃(60.7 mg, 37%): **^{1}H NMR** (300 MHz, $CDCl_3$) δ 7.68 (br, 6H), 7.58 -7.29 (m, 15H), 7.17-7.14 (m, 2H), 7.12-6.95 (m, 4H), 6.65 (s, 2H), 5.10 (br, 2H), 4.72 (br, 2H), 3.57 (d, *J* = 12 Hz, 2H), 2.68-2.61 (m, 1H), 2.52-2.43 (m, 1H), 0.82 (br, 6H), 0.63 (s, 9H). **^{13}C{^{1}H} NMR** (75 MHz, $CDCl_3$) δ 174.2, 143.4, 140.7, 135.8, 131.7, 131.4, 128.7, 128.2, 128.0, 127.9, 127.6, 126.7, 125.2, 66.7, 63.2, 48.9, 46.5, 36.2, 29.1, 19.4. **HRMS** (EI) m/z: $[M]^+$ calcd for $C_{57}H_{52}O_2$: 768.3967. Found: 768.3961.

7-Isopropyl-5,9-diphenylbenzo[m]tetraphene (6a)

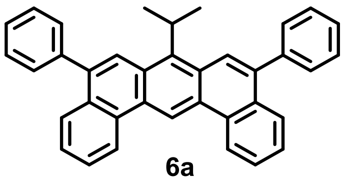

6a

According to general procedure **V**: Step 1: **5a** (0.1 mmol, 68 mg), Pd/C (0.01 mmol, 1 mg), Ethyl acetate (2 mL) at H_2 for 24 h. Step 2: DDQ (0.4 mmol, 90 mg), CH_2Cl_2 (2 mL) at rt for 3 h. The crude product was purified by flash column chromatography (Hexane/Ether) provided **6a** as a brown solid; m.p.:149-150 ℃ (44.7 mg, 95%). Compound **6a** dissolves in 6 mL of EA and 1 mL of hexane. A block shaped, colorless crystals suitable for X-ray diffraction were obtained after few days at room temperature: **^{1}H NMR** (300 MHz, $CDCl_3$) δ 10.13 (s, 1H), 9.14 (d, J = 9 Hz, 2H), 8.30 (s, 2H), 7.93 (d, J = 9 Hz, 3H), 7.80-7.65 (m, 3H), 7.64-7.48 (m, 10H), 4.58 (sept, J = 6 Hz, 1H), 1.77 (d, J = 6 Hz, 6H). **^{13}C{^{1}H} NMR** (75 MHz, $CDCl_3$) δ 141.8, 141.6, 141.5, 138.2, 131.8, 130.7, 130.2, 130.1, 128.8, 128.4, 127.5, 126.9, 126.8, 124.5, 123.4, 115.3, 115.2, 28.7, 28.6, 23.2, 23.1. **HRMS** (EI) m/z: $[M]^+$ calcd for $C_{37}H_{28}$: 472.2191. Found: 472.2191.

7-Isopropyl-5,9-bis(4-methoxyphenyl)-4a,7a,13b,14b-tetrahydrobenzo[m]tetraphene (6b)

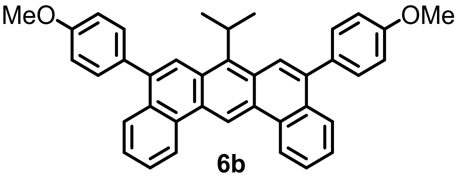

6b

According to general procedure **V**: Step 1: **5b** (0.1 mmol, 70 mg), Pd/C (0.01 mmol, 1 mg), Ethyl acetate (2 mL) at H_2 for 24 h. Step 2: DDQ (0.4 mmol, 90 mg), CH_2Cl_2 (2 mL) at rt for 3 h. The crude product was purified by flash column chromatography (Hexane/Ether) provided **6b** as a brown solid; m.p.:147-148 ℃ (40.8 mg, 78%): **^{1}H NMR** (300 MHz, $CDCl_3$) δ 10.12 (s, 1H), 9.13 (d, J = 9 Hz, 2H), 8.28 (s, 2H), 7.96 (d, J = 9 Hz, 2H), 7.80-7.69 (m, 2H), 7.62-7.52 (m, 6H), 7.11 (d, J = 9 Hz, 4H), 4.56 (sept, J = 6 Hz, 1H), 3.94 (s, 6H), 1.77 (d, J = 6 Hz, 6H). **^{13}C{^{1}H} NMR** (75 MHz, $CDCl_3$) δ 159.1, 141.2, 137.8, 133.9, 131.8, 131.2, 130.9, 128.5, 126.9, 126.7, 124.5, 123.4, 115.2, 113.9, 55.4, 28.6, 23.2. **HRMS** (EI) m/z: $[M]^+$ calcd for $C_{39}H_{32}O_2$: 532.2402. Found: 532.2398.

Methyl-4-(7-isopropyl-9-(4-methoxyphenyl)benzo[m]tetraphen-5-yl)benzoate (6c)

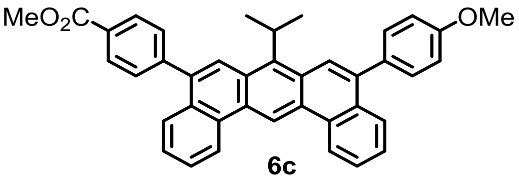

6c

According to general procedure **V**: Step 1: **5c** (0.1 mmol, 72 mg), Pd/C (0.01 mmol, 1 mg) Ethyl acetate (2 mL) at H_2 for 24 h. Step 2: DDQ (0.4 mmol, 90 mg), CH_2Cl_2 (2 mL) at rt for 3 h. The crude product was purified by flash column chromatography (Hexane/Ether) provided **6c** as a brown solid; m.p.:145-146 ℃ (42.5 mg, 82%): **^{1}H NMR** (300 MHz, $CDCl_3$) δ 10.12 (s, 1H), 9.13 (d, J = 9 Hz, 2H), 8.37-8.18 (m, 4H), 7.96 (d, J = 9 Hz, 1H), 7.86 (d, J = 9 Hz, 1H), 7.81-7.68 (m, 4H), 7.64-7.52 (m, 4H), 7.15-7.04 (m, 2H), 4.58 (sept, J = 6 Hz, 1H), 4.03 (s, 3H), 3.97 (s, 3H), 1.77 (d, J = 6 Hz, 6H). **^{13}C{^{1}H} NMR** (75 MHz, $CDCl_3$) δ 167.7, 167.1, 159.1, 146.4, 141.6, 137.9, 137.1, 133.8, 131.8, 131.7, 131.3, 131.15, 130.9, 130.3, 130.2, 130.1, 129.8, 129.2, 128.9, 128.7, 128.6, 128.1, 126.9, 126.8, 124.9, 124.4, 123.5, 115.3, 113.8, 55.4, 52.3, 28.8, 23.3, 23.2. **HRMS** (EI) m/z: $[M]^+$ calcd for $C_{40}H_{32}O_3$: 560.2351. Found: 560.2347.

Dimethyl-4,4'-(7-isopropylbenzo[m]tetraphene-5,9-diyl)dibenzoate (6d)

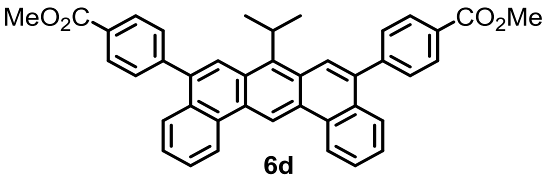

6d

According to general procedure **V**: Step 1: **5d** (0.1 mmol, 78 mg), Pd/C (0.01 mmol, 1 mg), Ethyl acetate (2 mL) at H_2 for 24 h. Step 2: DDQ (0.4 mmol, 90 mg), CH_2Cl_2 (2 mL) at rt for 3 h. The crude product was purified by flash column chromatography (Hexane/Ether) provided **6d** as a brown solid; m.p.:155-156 ℃ (47.2 mg, 80%): **^{1}H NMR** (300 MHz, $CDCl_3$) δ 10.12 (s, 1H), 9.14 (d, J = 9 Hz, 2H), 8.36-8.20 (m, 6H), 7.87 (d, J = 9 Hz, 2H), 7.82-7.69 (m, 6H), 7.64-7.56 (m, 2H), 4.57 (sept, J = 6 Hz, 1H), 4.01 (s, 6H), 1.77 (d, J = 6 Hz, 6H). **^{13}C{^{1}H} NMR** (75 MHz, $CDCl_3$) δ 167.1, 146.2, 142.04, 137.3, 131.7, 130.2, 130.1, 129.8,

129.3, 129.1, 128.2, 127.07, 126.9, 126.6, 124.7, 123.5, 115.4, 115.3, 52.26, 28.6, 23.3, 23.2. **HRMS** (EI) m/z: $[M]^+$ calcd for $C_{41}H_{32}O_4$: 588.2301. Found: 588.2298.

5-(4-chlorophenyl)-7-isopropyl-9-(4-methoxyphenyl)benzo[m]tetraphene (6e)

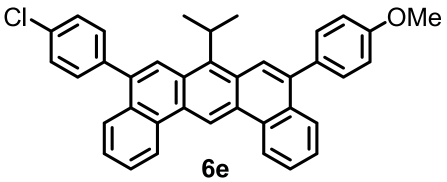

6e

According to general procedure **V**: Step 1: **5e** (0.1 mmol, 74 mg), Pd/C (0.01 mmol, 1 mg) Ethyl acetate (2 mL) at H_2 for 24 h. Step 2: DDQ (0.4 mmol, 90 mg), CH_2Cl_2 (2 mL) at rt for 3 h. The crude product was purified by flash column chromatography (Hexane/Ether) provided **6e** : as a brown solid; m.p.:178-179 °C (43.8 mg, 75%): **^{1}H NMR** (300 MHz, $CDCl_3$) ^{1}H NMR (300 MHz, $CDCl_3$) δ 10.12 (s, 1H), 9.13 (d, J = 9 Hz, 2H), 8.27 (s, 2H), 7.95 (d, J = 9 Hz, 2H), 7.72-7.68 (m, 2H), 7.62-7.58 (m, 6H), 7.12 (d, J = 9 Hz, 4H), 4.58 (m, 1H), 3.95 (s, 3H), 1.77 (d, J = 9 Hz, 6H). **^{13}C{^{1}H} NMR** (75 MHz, $CDCl_3$) δ 141.7, 139.9, 137.0, 133.6, 131.7, 131.4, 130.4, 128.9, 128.7, 128.3, 126.9, 126.9, 126.6, 124.6, 123.5, 115.3, 28.7, 23.2. **HRMS** (EI) m/z: $[M]^+$ calcd for $C_{38}H_{29}ClO$: 536.1907. Found: 536.1899.

5,9-bis(4-chlorophenyl)-7-isopropylbenzo[m]tetraphene (6f)

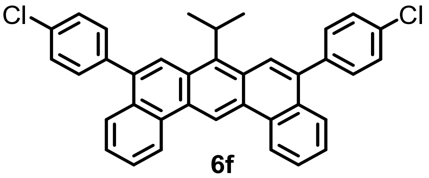

6f

According to general procedure **V**: Step 1: **5f** (0.1 mmol, 74 mg), Pd/C (0.01 mmol, 1 mg) Ethyl acetate (2 mL) at H_2 for 24 h. Step 2: DDQ (0.4 mmol, 90 mg), CH_2Cl_2 (2 mL) at rt for 3 h. The crude product was purified by flash column chromatography (Hexane/Ether) provided **6f** : as a brown solid; m.p.:200-201 °C (45.5 mg, 75%): **^{1}H NMR** (300 MHz, $CDCl_3$) δ 10.11 (s, 1H), 9.13 (d, J = 9 Hz, 2H), 8.27 (s, 2H), 7.89 (d, J = 9 Hz, 2H), 7.82-7.75 (m, 2H), 7.69-7.45 (m, 10H), 4.57 (sept, J = 6 Hz, 1H), 1.78 (d, J = 6 Hz, 6H). **^{13}C{^{1}H} NMR** (75 MHz, $CDCl_3$) δ 141.7, 139.9, 137.0, 133.6, 131.7, 131.4, 130.4, 128.9, 128.7, 128.3, 126.9, 126.9, 126.6, 124.6, 123.5, 115.3, 28.7, 23.2. **HRMS** (EI) m/z: $[M]^+$ calcd for $C_{37}H_{26}Cl_2$: 540.1412. Found: 540.1409.

8-isopropyl-6,10-diphenylnaphtho[2,3-a]pentaphene (6g)

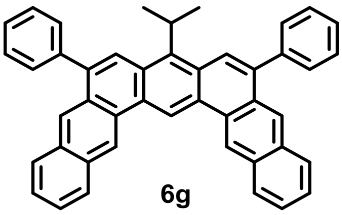

6g

According to general procedure **V**: Step 1: **5h** (0.1 mmol, 73 mg), [1]Pd/C (0.01 mmol, 1 mg), $Pd(OAc)_2$, (0.002 mmol, 2 mg), Ethyl acetate (2 mL) at H_2 for 24 h. Step 2: DDQ (0.4 mmol, 90 mg), CH_2Cl_2 (2 mL) at rt for 3 h. The crude product was purified by flash column chromatography (Ether/Hexane) provided **6g** : as a brown solid; m.p.: m.p.: 185-186 ℃ (25.3 mg, 50 %): **^{1}H NMR** (300 MHz, $CDCl_3$) δ 10.33 (s, 1H), 9.65 (s, 2H), 8.39-8.35 (m, 4H), 8.19 (s, 2H), 7.96 (d, *J* = 9 Hz, 2H), 7.76-7.50 (m, 14H), 4.53 (sept, *J* = 6 Hz, 1H), 1.78 (d, *J* = 9 Hz, 6H). **^{113}C{^{1}H} NMR** (75 MHz, $CDCl_3$) δ 141.9, 141.6, 138.4, 131.9, 131.9, 130.2, 130.1, 129.6, 129.2, 128.8, 128.6, 128.3, 127.6, 126.2, 125.9, 125.8, 124.7, 122.3, 28.6, 23.2 **HRMS** (EI) m/z: $[M]^+$ calcd for $C_{45}H_{32}$: 572.2504. Found: 572.2498.

4. Single crystal data of 6a

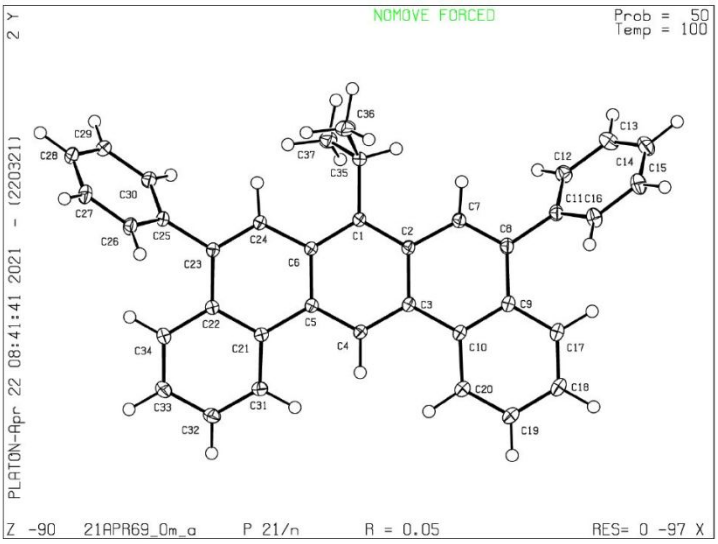

Table S1. Crystal Data and Structure Refinement for **6a**.

Empirical formula	$C_{37}H_{28}$	
Formula weight	472.59	
Temperature/K	100(2)	
Crystal system	monoclinic	
Space group	$P2_1/n$	
Unit cell dimensions	a = 9.5050(2) Å	α = 90°
	b = 16.5578(3) Å	β = 101.2470(10)°
	c = 15.8629(3) Å	γ = 90°
Volume	2448.59(8) $Å^3$	
Z	4	
Density (calculated)	1.282 g/cm^3	
Absorption coefficient	0.072 mm^{-1}	
F(000)	1000	
Crystal size	0.500 × 0.337 × 0.194 mm^3	
Radiation	MoKα (λ = 0.71073)	
2Θ range for data collection	3.592° to 72.808°	
Index ranges	$-15 \le h \le 15$, $-27 \le k \le 27$, $-26 \le l \le 26$	
Reflections collected	103794	
Independent reflections	11909 [R_{int} = 0.0513, R_{sigma} = 0.0288]	
Data/restraints/parameters	11909/2/446	
Goodness-of-fit on F^2	1.050	
Final R indexes [I>=2σ (I)]	R_1 = 0.0488, wR_2 = 0.1277	
Final R indexes [all data]	R_1 = 0.0626, wR_2 = 0.1362	
Largest diff. peak/hole	0.57/-0.21 e $Å^{-3}$	
CCDC number	2181649	

5. Electrochemical analysis

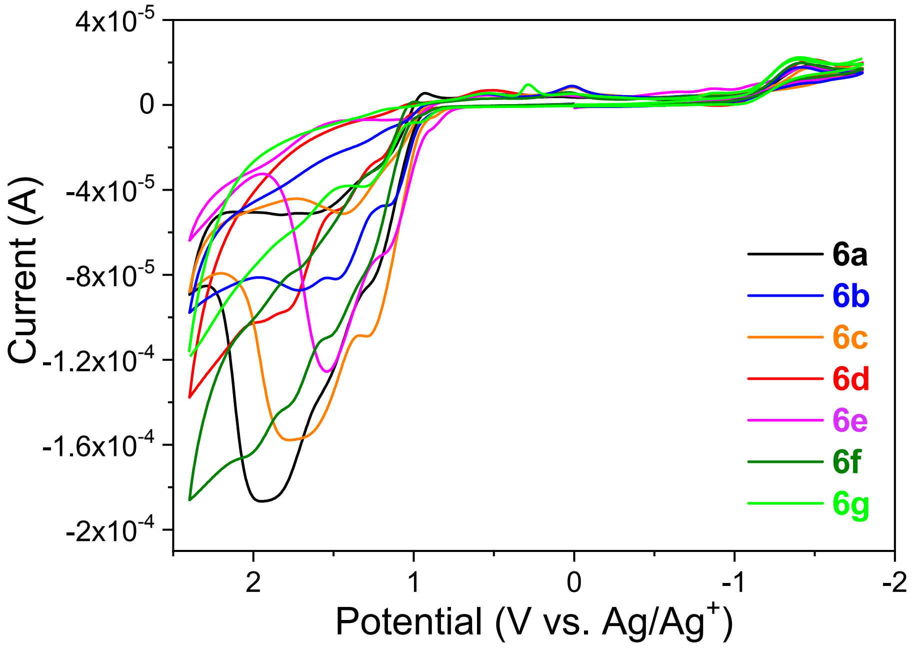

Figure S1. Cyclic voltammogram of **6**; measurements were carried out in CH_2Cl_2 containing 0.1 M tetrabutylammonium perchlorate.

6. TD-DFT excitation analysis[3]

Table S1. Summary of TDDFT Analysis of the First Three Excited Singlet States.[a]

Compound	Excited state	Excitation energy	Oscillator strength	Major contributing configurations[b]			
**6a**	1	327.26 nm (3.7886 eV)	0.0013	H-1	→	L	45.7%
				H	→	L+1	41.6%
	2	320.22 nm (3.8718 eV)	0.0291	H	→	L	82.4%
				H-1	→	L+1	13.0%
	3	282.93 nm (4.3821 eV)	2.0536	H	→	L+1	47.8%
				H-1	→	L	43.7%
**6b**	1	328.03 nm (3.7797 eV)	0.0043	H-1	→	L	44.1%
				H	→	L+1	38.6%
	2	321.16 nm (3.8605 eV)	0.0223	H	→	L	80.7%
				H-1	→	L+1	12.6%
	3	285.97 nm (4.3356 eV)	2.2047	H	→	L+1	49.0%
				H-1	→	L	41.6%
**6c**	1	328.92 nm (3.7694 eV)	0.0038	H	→	L	23.8%
				H-1	→	L	22.1%
				H	→	L+1	16.5%
				H-1	→	L+1	14.0%
				H	→	L+2	8.9%
	2	321.38 nm (3.8579 eV)	0.0195	H	→	L	50.3%
				H-1	→	L	19.1%
				H		L+1	19.0%
	3	288.29 nm (4.3007 eV)	2.2724	H	→	L+1	45.8%
				H-1	→	L	42.3%
**6d**	1	328.03 nm (3.7797 eV)	0.0001	H-1	→	L	40.2%
				H	→	L+1	35.3%
				H	→	L+3	8.5%
	2	321.81 nm (3.8527 eV)	0.0168	H	→	L	77.9%
				H-1	→	L+1	12.3%
	3	289.3 nm (4.2857 eV)	2.3481	H-1	→	L	44.8%
				H	→	L+1	44.1%
**6e**	1	328.31 nm (3.7764 eV)	0.0052	H-1	→	L	32.1%
				H	→	L+1	30.6%
				H	→	L	14.7%
				H-1	→	L+1	6.3%
	2	320.75 nm (3.8654 eV)	0.0219	H	→	L	64.9%

				H-1	→	L	12.8%
				H	→	L+1	8.2%
				H-1	→	L+1	7.8%
	3	285.55 nm (4.342 eV)	2.2066	H	→	L+1	47.5%
				H-1	→	L	42.3%
**6f**	1	327.4 nm (3.7869 eV)	0.0011	H-1	→	L	44.7%
				H	→	L+1	41.3%
	2	320.53 nm (3.8681 eV)	0.0246	H	→	L	81.8%
				H-1	→	L+1	13.1%
	3	284.47 nm (4.3584 eV)	2.2132	H	→	L+1	47.4%
				H-1	→	L	43.8%
**6g**	1	347.27 nm (3.5703 eV)	0.0035	H-1	→	L	41.8%
				H	→	L+1	35.4%
	2	340.64 nm (3.6397 eV)	0.0237	H	→	L	71.3%
				H-1	→	L+1	17.4%
				H-2	→	L+2	6.6%
	3	314.01 nm (3.9484 eV)	1.3732	H	→	L+1	37.5%
				H-1	→	L	29.9%
				H	→	L+2	14.2%
				H-2	→	L	10.6%

[a] At the ωB97X-D/6-31G(d,p) level of theory, [b] H = HOMO and L = LUMO.

Table S2. Natural Transition Orbitals (NTOs) of the S1–S3 States of **6a**, Representative of Symmetrically Substituted DBA.

State	Hole NTO	Electron NTO	Contribution
S1	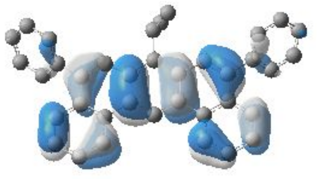	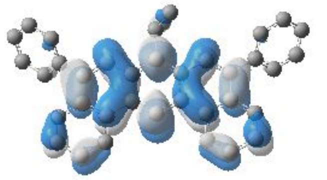	0.50
	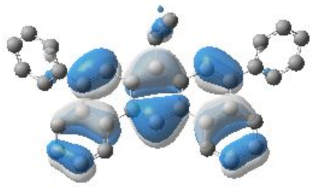	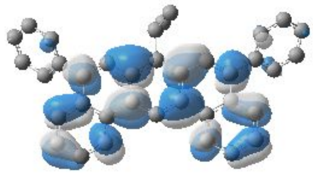	0.45
S2	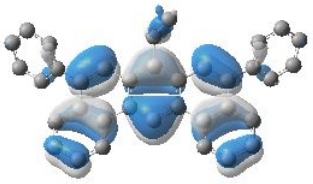	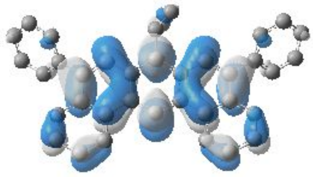	0.84
S3	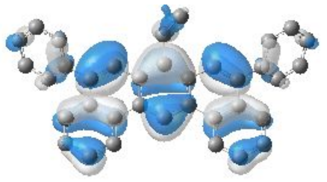	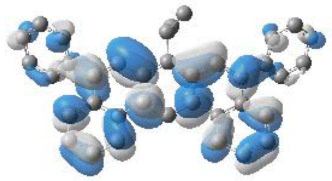	0.51
	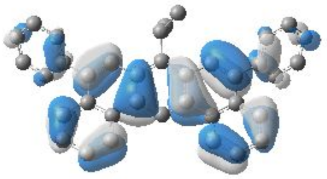	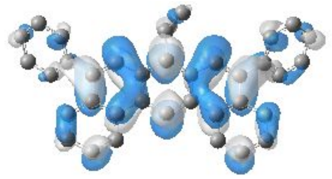	0.47

Table S3. Natural Transition Orbitals (NTOs) of the S1–S3 States of **6c**, Representative of Asymmetrically Substituted DBA.

State	Hole NTO	Electron NTO	Contribution
S1	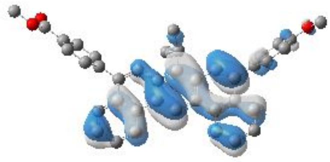	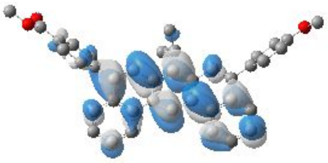	0.61
	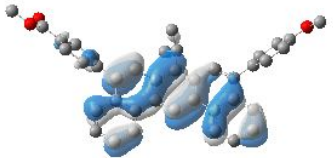	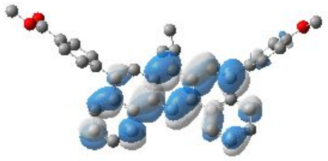	0.35
S2	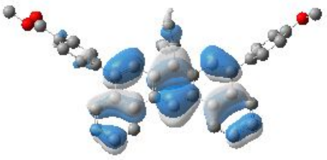	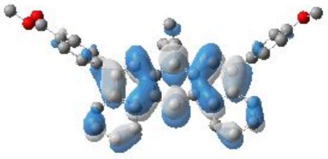	0.81
S3	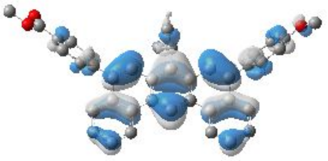	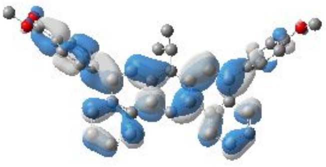	0.51
	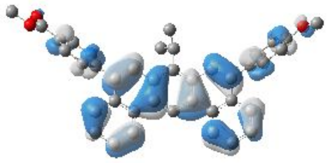	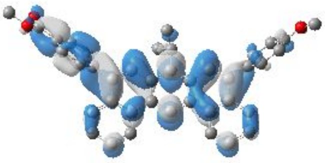	0.46

Table S4. Natural Transition Orbitals (NTOs) of the S1–S3 States of **6g**.

State	Hole NTO	Electron NTO	Contribution
S1	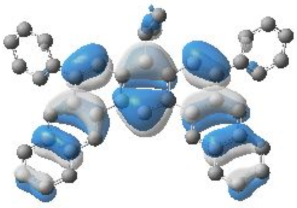	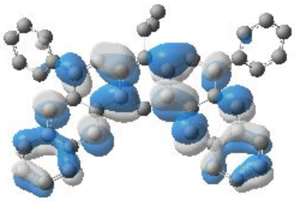	0.48
	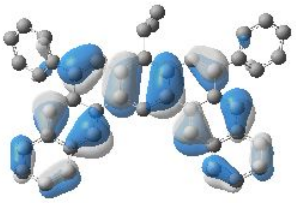	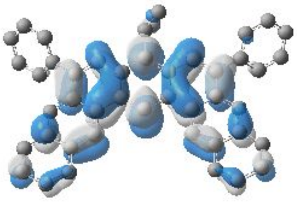	0.43
S2	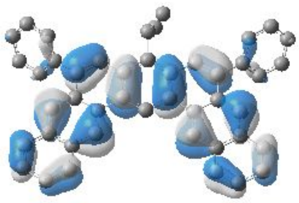	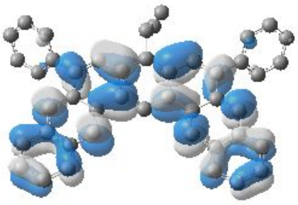	0.72
S3	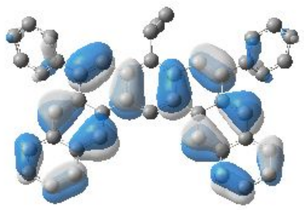	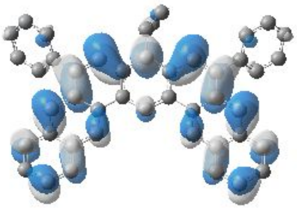	0.53
	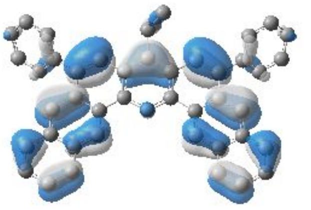	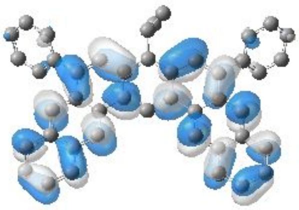	0.44

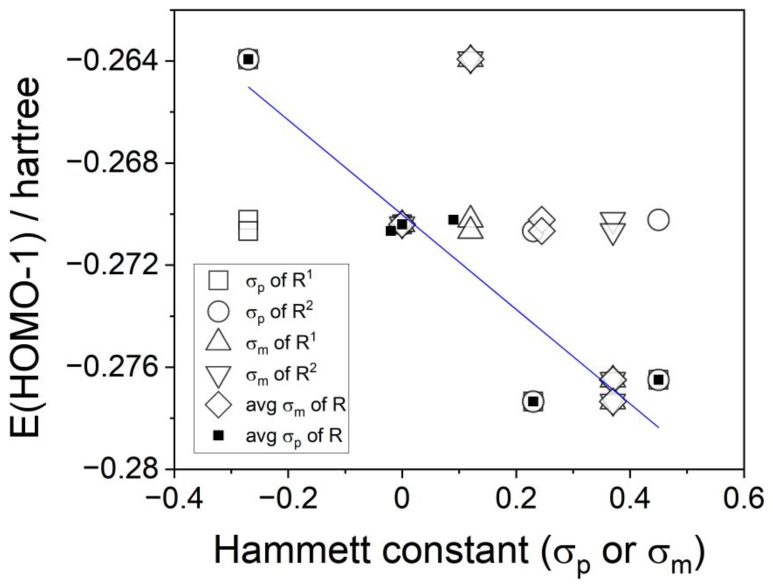

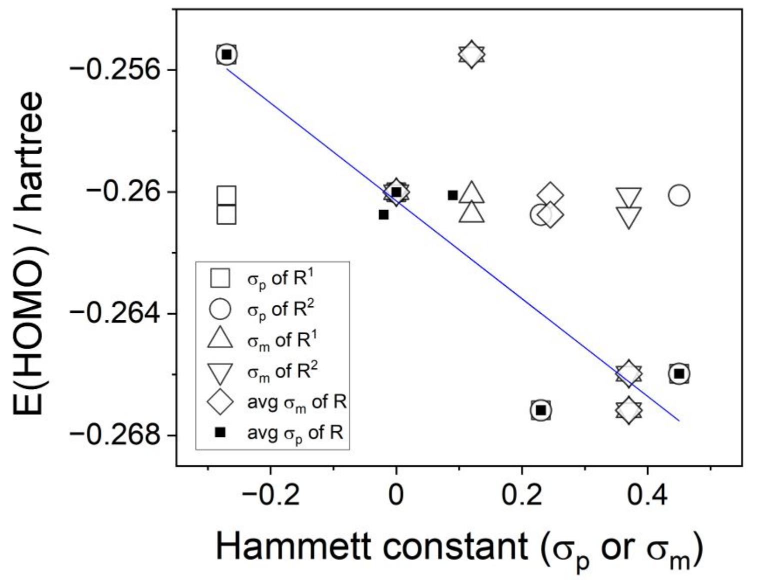

Figure S2. Correlation between the DFT computed HOMO (top) and HOMO-1 (bottom) energy with Hammett substituent constants for **6a**–**6f**. Blue line highlights the best correlation of orbital energy to the average σ_p of R^1 and R^2 substituents.

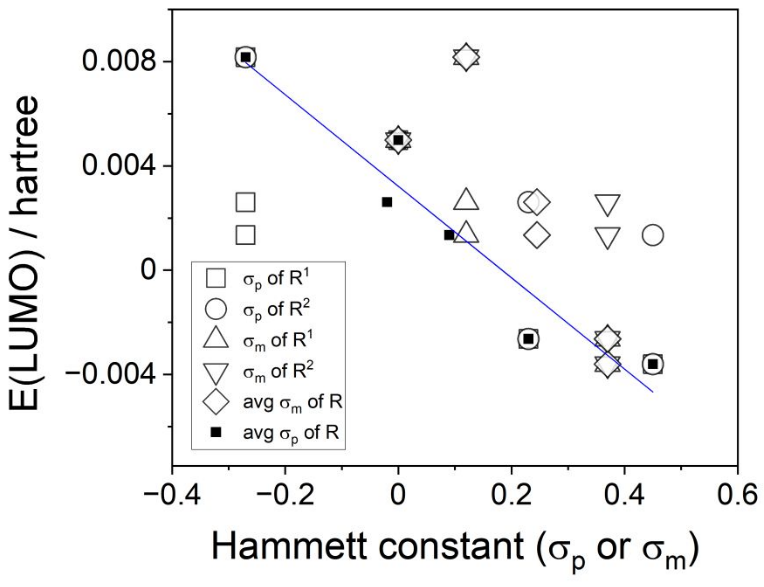

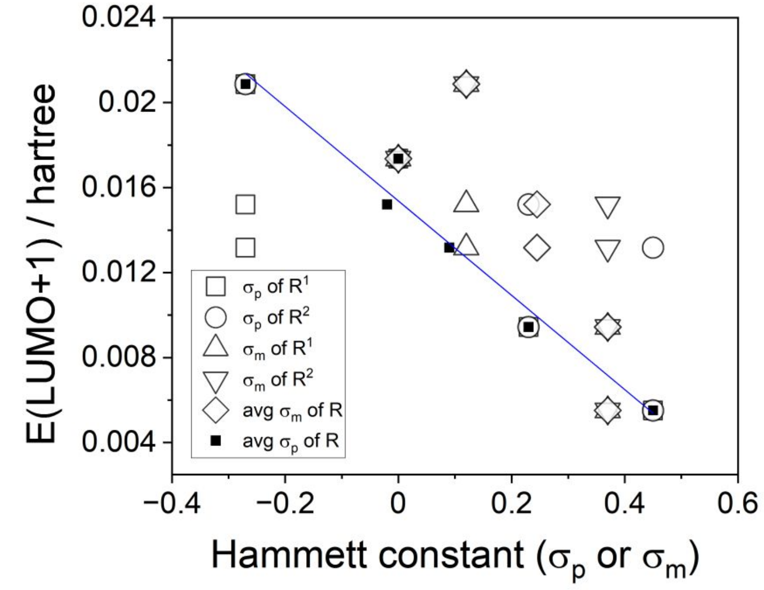

Figure S3. Correlation between the DFT computed LUMO (top) and LUMO+1 (bottom) energy with Hammett substituent constants for **6a**–**6f**. Blue line highlights the best correlation of orbital energy to the average σ_p of R^1 and R^2 substituents.

7. Cartesian coordinates for optimized geometries[3]

Structures were optimized in vacuo at the ωB97X-D/6-31G(d,p) level of theory. Symmetry was not constrained. No imaginary frequency was found for the located stationary state.

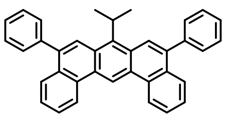

6a

C	-0.023188	-0.892989	-0.332700
C	-1.248554	-0.195690	-0.223649
C	-1.262861	1.226016	-0.137147
C	-0.050153	1.905540	-0.104509
H	-0.059919	2.982433	-0.027507
C	1.174607	1.246723	-0.123341
C	1.186273	-0.174738	-0.210860
C	-2.515410	-0.879591	-0.125698
H	-2.531953	-1.960072	-0.058171
C	-3.705341	-0.239367	-0.012997
C	-3.754845	1.211096	-0.052123
C	-2.542185	1.938757	-0.102090
C	-4.939999	-1.051589	0.164696
C	-5.270362	-2.043515	-0.762908
H	-4.641751	-2.177461	-1.638198
C	-6.394995	-2.842427	-0.579743
H	-6.638893	-3.605115	-1.312625
C	-7.206997	-2.660181	0.534948
H	-8.085142	-3.281703	0.678112
C	-6.887228	-1.675463	1.466534
H	-7.511606	-1.532070	2.342818
C	-5.763810	-0.877848	1.283010
H	-5.511112	-0.117636	2.016089
C	-4.981862	1.906068	-0.070341
H	-5.906980	1.341218	-0.065278
C	-5.022007	3.282554	-0.109191
H	-5.976213	3.799001	-0.126250
C	-3.825416	4.008237	-0.139289
H	-3.845628	5.092746	-0.174746
C	-2.616032	3.346184	-0.141606
H	-1.710400	3.938255	-0.193855
C	2.441150	1.981099	-0.071104
C	3.666247	1.274272	-0.018314
C	3.641554	-0.176644	0.012160
C	2.462874	-0.835414	-0.106760
H	2.491270	-1.913197	-0.048392
C	4.887545	-0.972001	0.184713
C	5.710375	-0.795249	1.303162
H	5.448957	-0.043256	2.041636
C	6.843534	-1.580511	1.480430
H	7.467144	-1.435137	2.356941
C	7.173986	-2.555607	0.542501
H	8.059564	-3.167553	0.681058
C	6.363062	-2.740712	-0.572559
H	6.615248	-3.495893	-1.310375
C	5.228988	-1.953970	-0.749609
H	4.602154	-2.088784	-1.626297
C	2.490875	3.389947	-0.096708
H	1.575352	3.966758	-0.147635
C	3.688505	4.072840	-0.080612
H	3.690012	5.157828	-0.105599
C	4.897392	3.367779	-0.049745
H	5.842504	3.900939	-0.056161
C	4.880996	1.990561	-0.022729
H	5.815549	1.441416	-0.015986
C	-0.048012	-2.399002	-0.602885
H	-1.069614	-2.633787	-0.903372
C	0.222658	-3.245066	0.650865
H	-0.483084	-2.985698	1.445675
H	0.103387	-4.309181	0.422600
H	1.230265	-3.099606	1.048538
C	0.781357	-2.840702	-1.822552
H	1.859189	-2.838183	-1.654445
H	0.577400	-2.191006	-2.678244
H	0.497974	-3.861655	-2.097830

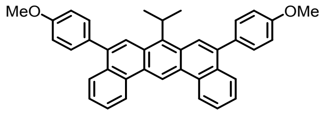

6b

C	-0.004030	-0.456436	0.434636
C	1.229534	0.225512	0.319753
C	1.257765	1.641429	0.167723
C	0.051707	2.328019	0.079911

H	0.071800	3.400422	-0.044479
C	-1.178771	1.680166	0.103586
C	-1.204651	0.264113	0.253989
C	2.491954	-0.472000	0.279978
H	2.499641	-1.554546	0.260071
C	3.691055	0.151586	0.166626
C	3.750983	1.602480	0.140600
C	2.543892	2.341269	0.126418
C	4.921849	-0.676982	0.061739
C	5.215101	-1.641725	1.034316
H	4.559736	-1.740786	1.894467
C	6.327962	-2.458123	0.924667
H	6.558728	-3.200733	1.680475
C	7.185943	-2.331120	-0.171575
C	6.911552	-1.377034	-1.152660
H	7.552802	-1.260350	-2.017962
C	5.787938	-0.564141	-1.026113
H	5.576851	0.167407	-1.800466
C	4.982902	2.288824	0.156882
H	5.902212	1.716573	0.204117
C	5.034461	3.665242	0.130264
H	5.992491	4.174676	0.147532
C	3.843975	4.400603	0.094011
H	3.872676	5.485397	0.076678
C	2.629271	3.748387	0.098502
H	1.727788	4.349064	0.098530
C	-2.437924	2.420855	-0.005757
C	-3.667538	1.721268	-0.049991
C	-3.655819	0.269677	-0.019864
C	-2.484083	-0.391149	0.149150
H	-2.519233	-1.470206	0.131642
C	-4.906161	-0.519115	-0.179254
C	-5.720819	-0.380737	-1.313175
H	-5.449800	0.340050	-2.078752
C	-6.854110	-1.155846	-1.479518
H	-7.480721	-1.055943	-2.358945
C	-7.209808	-2.099530	-0.509199
C	-6.413225	-2.253854	0.624415
H	-6.665434	-2.972321	1.395012
C	-5.274233	-1.464184	0.775783
H	-4.664034	-1.579604	1.666938
C	-2.476554	3.829791	-0.042681
H	-1.557283	4.401385	-0.000427
C	-3.668090	4.520297	-0.109928
H	-3.661074	5.605353	-0.132512
C	-4.881977	3.823542	-0.130764
H	-5.822757	4.363400	-0.163714
C	-4.876094	2.446318	-0.097318
H	-5.814564	1.903825	-0.095119
C	0.002847	-1.948409	0.774766
H	1.014731	-2.174793	1.112508
C	-0.241454	-2.849995	-0.445216
H	0.486669	-2.632089	-1.232255
H	-0.136258	-3.903267	-0.165301
H	-1.237259	-2.716185	-0.875405
C	-0.861001	-2.328867	1.991043
H	-1.933903	-2.328735	1.793924
H	-0.675896	-1.640876	2.820710
H	-0.591035	-3.337258	2.320979
O	8.250243	-3.173417	-0.190058
O	-8.339337	-2.808286	-0.762502
C	-8.738395	-3.774528	0.183013
H	-9.651440	-4.223053	-0.208511
H	-7.978333	-4.556043	0.308003
H	-8.950300	-3.321380	1.159676
C	9.145386	-3.084719	-1.275346
H	8.647875	-3.301291	-2.229075
H	9.614848	-2.094564	-1.332777
H	9.915172	-3.835205	-1.095560

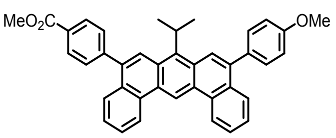

6c

C	0.464245	-0.305399	0.543988
C	1.734520	0.284354	0.347527
C	1.849889	1.685695	0.119410
C	0.689701	2.448525	0.041844
H	0.776488	3.509352	-0.139997
C	-0.578926	1.888586	0.147212
C	-0.691494	0.486634	0.371182
C	2.945273	-0.498839	0.300143
H	2.880790	-1.578882	0.340012
C	4.177445	0.035260	0.110671
C	4.331570	1.475282	0.002730
C	3.176679	2.293179	-0.008155
C	5.346783	-0.878547	0.011836

C	5.611483	-1.805277	1.028639
H	4.982776	-1.812217	1.913968
C	6.664156	-2.699107	0.930473
H	6.873445	-3.412326	1.720058
C	7.488358	-2.690713	-0.198554
C	7.241190	-1.776548	-1.223943
H	7.857258	-1.751910	-2.114704
C	6.178133	-0.884449	-1.108482
H	5.987045	-0.184837	-1.916801
C	5.605922	2.075484	-0.063418
H	6.485820	1.444180	-0.018045
C	5.747480	3.441799	-0.167748
H	6.737050	3.884779	-0.213326
C	4.607902	4.254029	-0.201620
H	4.707874	5.331853	-0.280035
C	3.353686	3.687707	-0.117117
H	2.494562	4.347583	-0.118104
C	-1.789687	2.707636	0.049534
C	-3.064151	2.092714	0.080687
C	-3.143637	0.647256	0.183292
C	-2.014313	-0.084135	0.348485
H	-2.121363	-1.157764	0.390251
C	-4.443576	-0.069644	0.093616
C	-5.276945	0.079187	-1.022079
H	-4.981946	0.753367	-1.820028
C	-6.461681	-0.635370	-1.124102
H	-7.097009	-0.520453	-1.994440
C	-6.836240	-1.515484	-0.106203
C	-6.013164	-1.671382	1.008804
H	-6.320174	-2.355831	1.792039
C	-4.829376	-0.953906	1.106629
H	-4.195976	-1.065049	1.981345
C	-1.736140	4.112974	-0.049738
H	-0.779826	4.621657	-0.063472
C	-2.880795	4.879381	-0.107413
H	-2.802603	5.959449	-0.178720
C	-4.138292	4.266819	-0.055300
H	-5.041629	4.867453	-0.080342
C	-4.223024	2.895215	0.041230
H	-5.195926	2.420839	0.101254
C	0.385639	-1.775841	0.960421
H	1.392667	-2.052409	1.274010
C	0.036705	-2.717131	-0.202805
H	0.746423	-2.587078	-1.025246
H	0.083465	-3.760073	0.126733
H	-0.964383	-2.538408	-0.603761
C	-0.454563	-2.038545	2.223618
H	-1.532327	-1.988689	2.061977
H	-0.202448	-1.318901	3.007445
H	-0.230282	-3.041506	2.600437
C	-8.095147	-2.308562	-0.159675
O	-8.452022	-3.085116	0.698075
O	-8.805441	-2.070768	-1.274918
C	-10.023521	-2.803902	-1.385696
H	-9.827479	-3.878493	-1.384952
H	-10.691603	-2.568237	-0.554248
H	-10.468026	-2.497511	-2.331525
O	8.494429	-3.601452	-0.202995
C	9.354191	-3.632113	-1.319812
H	8.809997	-3.867708	-2.243008
H	9.883775	-2.679778	-1.448821
H	10.080163	-4.420690	-1.121926

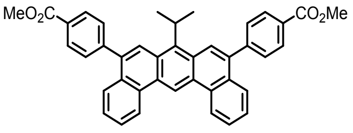

6d

C	0.045451	-0.078330	0.505376
C	1.257893	0.626197	0.323420
C	1.249809	2.037664	0.131685
C	0.026900	2.695967	0.066349
H	0.019936	3.763724	-0.092899
C	-1.187380	2.023625	0.155168
C	-1.176118	0.613049	0.352143
C	2.532988	-0.045448	0.254368
H	2.564583	-1.127610	0.265565
C	3.710066	0.602613	0.072628
C	3.740023	2.052323	0.006200
C	2.517416	2.764215	0.025154
C	4.951147	-0.204087	-0.074496
C	5.306992	-1.133578	0.907781
H	4.694174	-1.220362	1.799545
C	6.435927	-1.929172	0.760420
H	6.706817	-2.643640	1.528865
C	7.231236	-1.804369	-0.378872
C	6.884212	-0.880167	-1.366244
H	7.509095	-0.801893	-2.249151

C	5.756454	-0.088514	-1.214888
H	5.484217	0.621222	-1.989701
C	4.956659	2.763434	-0.044952
H	5.890560	2.213441	-0.025549
C	4.976357	4.139624	-0.103760
H	5.922687	4.669227	-0.139366
C	3.769477	4.848671	-0.104105
H	3.773992	5.933097	-0.144754
C	2.570351	4.171741	-0.034799
H	1.656686	4.753044	-0.007497
C	-2.465210	2.734956	0.067151
C	-3.680838	2.010658	0.091809
C	-3.633814	0.562964	0.177941
C	-2.444449	-0.070123	0.326613
H	-2.459188	-1.149246	0.356340
C	-4.868058	-0.262732	0.093593
C	-5.718487	-0.185188	-1.016338
H	-5.488025	0.512222	-1.815584
C	-6.838144	-0.999062	-1.110793
H	-7.487365	-0.939245	-1.976437
C	-7.128558	-1.908202	-0.091024
C	-6.287773	-1.993405	1.018307
H	-6.529829	-2.702023	1.802816
C	-5.169233	-1.177133	1.108453
H	-4.522419	-1.233765	1.978544
C	-2.535137	4.140545	-0.017993
H	-1.627705	4.731964	-0.029038
C	-3.742709	4.804027	-0.067307
H	-3.759610	5.887374	-0.128395
C	-4.941667	4.083119	-0.021028
H	-5.894097	4.602601	-0.040177
C	-4.905800	2.708497	0.061066
H	-5.832749	2.149086	0.115169
C	0.097498	-1.559926	0.884906
H	1.128249	-1.758916	1.179747
C	-0.186684	-2.499123	-0.297568
H	0.498309	-2.289942	-1.124648
H	-0.047342	-3.541777	0.005476
H	-1.204365	-2.396795	-0.682846
C	-0.699913	-1.921672	2.151441
H	-1.780594	-1.951938	2.005692
H	-0.492565	-1.205500	2.951414
H	-0.392081	-2.913552	2.497208
C	8.454301	-2.626496	-0.592348
O	9.172979	-2.544436	-1.563283
O	8.681360	-3.481516	0.418416
C	-8.315438	-2.806165	-0.136916
O	-8.599200	-3.609832	0.723068
O	-9.049756	-2.630234	-1.247818
C	9.841538	-4.298055	0.273013
H	9.767733	-4.915252	-0.625180
H	10.740015	-3.680872	0.202365
H	9.876659	-4.923078	1.164143
C	-10.201600	-3.464711	-1.351744
H	-9.914468	-4.518614	-1.351889
H	-10.882634	-3.286258	-0.516585
H	-10.675839	-3.197973	-2.295118

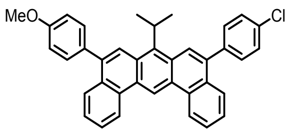

6e

C	0.005792	-0.463871	0.482918
C	1.232322	0.224748	0.336506
C	1.247130	1.637148	0.152582
C	0.034836	2.312852	0.065592
H	0.045238	3.382123	-0.083597
C	-1.190070	1.656571	0.120140
C	-1.202066	0.244087	0.302080
C	2.499366	-0.464144	0.295891
H	2.515511	-1.546712	0.300723
C	3.691774	0.165984	0.152324
C	3.739707	1.616355	0.092541
C	2.526996	2.345719	0.077986
C	4.927817	-0.655009	0.050559
C	5.241884	-1.593568	1.042075
H	4.599018	-1.677123	1.913236
C	6.360054	-2.403168	0.937085
H	6.607242	-3.125324	1.707351
C	7.202256	-2.295764	-0.173508
C	6.906718	-1.367954	-1.173558
H	7.535330	-1.267183	-2.050038
C	5.778204	-0.561285	-1.051383
H	5.550681	0.149502	-1.840288
C	4.966361	2.311987	0.076498
H	5.890571	1.747801	0.124107
C	5.006660	3.687779	0.017963

H	5.960780	4.204668	0.010617
C	3.810178	4.413315	-0.018767
H	3.830324	5.497579	-0.061053
C	2.600729	3.752402	0.017039
H	1.694782	4.346335	0.015752
C	-2.456094	2.385543	0.010775
C	-3.680880	1.676567	-0.007196
C	-3.654269	0.226897	0.054778
C	-2.477413	-0.422979	0.227984
H	-2.504241	-1.502480	0.236581
C	-4.897388	-0.578227	-0.083228
C	-5.709882	-0.470316	-1.217322
H	-5.445481	0.236029	-1.998004
C	-6.841268	-1.261867	-1.367794
H	-7.462040	-1.178376	-2.252448
C	-7.167082	-2.175031	-0.370688
C	-6.379420	-2.305198	0.765895
H	-6.648939	-3.018238	1.536492
C	-5.250319	-1.504647	0.901318
H	-4.636404	-1.590751	1.792615
C	-2.506294	3.793083	-0.052852
H	-1.591158	4.372252	-0.029659
C	-3.703810	4.472936	-0.122217
H	-3.705559	5.557299	-0.165494
C	-4.912501	3.766968	-0.118468
H	-5.857614	4.298906	-0.153088
C	-4.895698	2.390787	-0.057895
H	-5.830785	1.842745	-0.035535
C	0.028847	-1.947894	0.855564
H	1.046392	-2.157788	1.186652
C	-0.221161	-2.877735	-0.341825
H	0.495559	-2.670610	-1.142076
H	-0.103294	-3.923611	-0.040512
H	-1.223212	-2.762538	-0.763098
C	-0.818106	-2.309501	2.089401
H	-1.892818	-2.330636	1.902866
H	-0.635612	-1.598685	2.900142
H	-0.530964	-3.305389	2.441588
O	8.272991	-3.129382	-0.186065
C	9.153035	-3.060212	-1.285261
H	8.644633	-3.304116	-2.226528
H	9.613444	-2.068070	-1.372974
H	9.931218	-3.799945	-1.097469
Cl	-8.591659	-3.173691	-0.551149

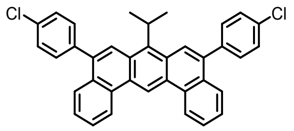

6f

C	0.023651	-0.460863	0.453000
C	1.246182	0.234238	0.305739
C	1.255892	1.649226	0.142482
C	0.040946	2.322030	0.074680
H	0.046842	3.393147	-0.060216
C	-1.181382	1.660866	0.129951
C	-1.187949	0.246034	0.293120
C	2.514982	-0.449974	0.243031
H	2.534420	-1.532496	0.232197
C	3.701878	0.188404	0.094444
C	3.747771	1.638729	0.055448
C	2.532598	2.363603	0.067098
C	4.938429	-0.627313	-0.043613
C	5.276387	-1.569412	0.931030
H	4.654094	-1.663858	1.815412
C	6.400840	-2.376196	0.793564
H	6.659236	-3.101288	1.556679
C	7.198216	-2.237050	-0.335171
C	6.886747	-1.308414	-1.322607
H	7.514883	-1.217847	-2.201329
C	5.760294	-0.510371	-1.170083
H	5.507667	0.208872	-1.942855
C	4.972503	2.337473	0.034934
H	5.900171	1.777304	0.061550
C	5.007945	3.714141	-0.001539
H	5.960283	4.233980	-0.013202
C	3.808826	4.436132	-0.010224
H	3.825453	5.520926	-0.034216
C	2.601647	3.771224	0.029602
H	1.694123	4.362269	0.050323
C	-2.450278	2.387509	0.039495
C	-3.673006	1.675073	0.023506
C	-3.642017	0.224871	0.068451
C	-2.461984	-0.423875	0.222952
H	-2.485945	-1.503407	0.219564
C	-4.884416	-0.581881	-0.066859
C	-5.706152	-0.465093	-1.193337
H	-5.449700	0.249073	-1.969549

C	-6.836946	-1.257802	-1.341946
H	-7.465039	-1.167557	-2.220737
C	-7.152781	-2.180829	-0.350669
C	-6.355639	-2.319682	0.778312
H	-6.617453	-3.040333	1.544456
C	-5.227100	-1.518078	0.911966
H	-4.605854	-1.611085	1.797479
C	-2.504757	3.795489	-0.008134
H	-1.591309	4.377345	0.013452
C	-3.704744	4.472463	-0.060161
H	-3.710030	5.557182	-0.091640
C	-4.911288	3.762863	-0.054337
H	-5.858105	4.292414	-0.075463
C	-4.890254	2.386172	-0.009301
H	-5.823588	1.835294	0.014325
C	0.054155	-1.950088	0.803707
H	1.075936	-2.163479	1.119396
C	-0.209224	-2.863277	-0.403662
H	0.496824	-2.643692	-1.210074
H	-0.085669	-3.912945	-0.118505
H	-1.216558	-2.744377	-0.810801
C	-0.776759	-2.329963	2.042986
H	-1.853763	-2.347089	1.870271
H	-0.582826	-1.631935	2.862131
H	-0.486103	-3.331417	2.375817
Cl	-8.576358	-3.180751	-0.528765
Cl	8.616518	-3.243560	-0.517800

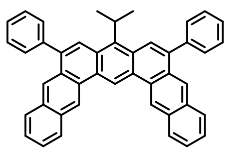

6g

C	-2.500800	-1.802234	-0.133841
C	-3.692373	-1.175842	0.002227
C	-3.757830	0.279101	-0.015738
C	-2.532749	1.023366	-0.063385
C	-1.249047	0.307324	-0.111426
C	-1.233434	-1.108022	-0.230138
C	-0.007618	-1.800488	-0.358434
C	1.201123	-1.084272	-0.221862
C	1.187354	0.330952	-0.101936
C	-0.037705	0.986764	-0.060569
C	2.480746	-1.751544	-0.124254
C	3.659942	-1.103497	0.013826
C	3.696501	0.352159	0.004828
C	2.456474	1.071961	-0.040628
C	4.895405	1.049213	0.010198
C	4.940055	2.452287	-0.000152
C	3.712807	3.172770	-0.028289
C	2.503572	2.457398	-0.055536
C	3.750680	4.596800	-0.042362
C	4.944085	5.262523	-0.028125
C	6.168706	4.542759	-0.002058
C	6.166691	3.176244	0.010295
C	-4.970572	0.951910	-0.019905
C	-5.043418	2.353684	-0.043204
C	-3.830862	3.098429	-0.075791
C	-2.607417	2.407293	-0.092915
C	-6.284448	3.052790	-0.042570
C	-6.313958	4.418795	-0.068539
C	-5.104023	5.162767	-0.099318
C	-3.897394	4.521190	-0.104376
H	-2.507380	-2.883719	-0.081175
C	-4.919895	-1.997809	0.181223
H	-0.048990	2.060723	0.054149
H	2.502621	-2.830263	-0.081319
C	4.901777	-1.905343	0.182987
H	5.831659	0.500984	0.012771
H	1.590406	3.039632	-0.110723
H	2.812715	5.144817	-0.064061
H	4.962724	6.347620	-0.037705
H	7.107674	5.086906	0.007419
H	7.100010	2.620627	0.029324
H	-5.895726	0.385195	-0.015471
H	-1.705895	3.006911	-0.153190
H	-7.206423	2.478674	-0.020040
H	-7.263713	4.943961	-0.066461
H	-5.144516	6.247115	-0.119883
H	-2.970627	5.087768	-0.129624
C	5.245857	-2.875585	-0.762431
C	6.376541	-3.668104	-0.589103
C	7.181304	-3.500291	0.533074
C	6.848276	-2.536584	1.481902
C	5.718768	-1.745406	1.308387
H	4.624066	-2.996420	-1.644709
H	6.630958	-4.414043	-1.335488

H	8.064224	-4.116720	0.668694
H	7.467065	-2.404830	2.363970
H	5.455670	-1.001844	2.054669
C	-5.253466	-2.979430	-0.756037
C	-6.371736	-3.787196	-0.572429
C	-7.173975	-3.623971	0.552180
C	-6.851000	-2.649298	1.493275
C	-5.734203	-1.842682	1.309350
H	-4.632703	-3.098094	-1.639079
H	-6.618385	-4.541701	-1.312800
H	-8.047149	-4.252379	0.695672
H	-7.467706	-2.520877	2.377284
H	-5.479305	-1.089866	2.049171
C	-0.032429	-3.299367	-0.666977
H	-1.052640	-3.523901	-0.980497
C	0.804435	-3.710896	-1.891942
H	1.881070	-3.715223	-1.716320
H	0.520917	-4.723744	-2.195517
H	0.607786	-3.038827	-2.731907
C	0.228981	-4.177584	0.566377
H	-0.481290	-3.937947	1.363383
H	0.110307	-5.235386	0.310283
H	1.234212	-4.043339	0.974028

8. ^1H and ^{13}C NMR Spectra

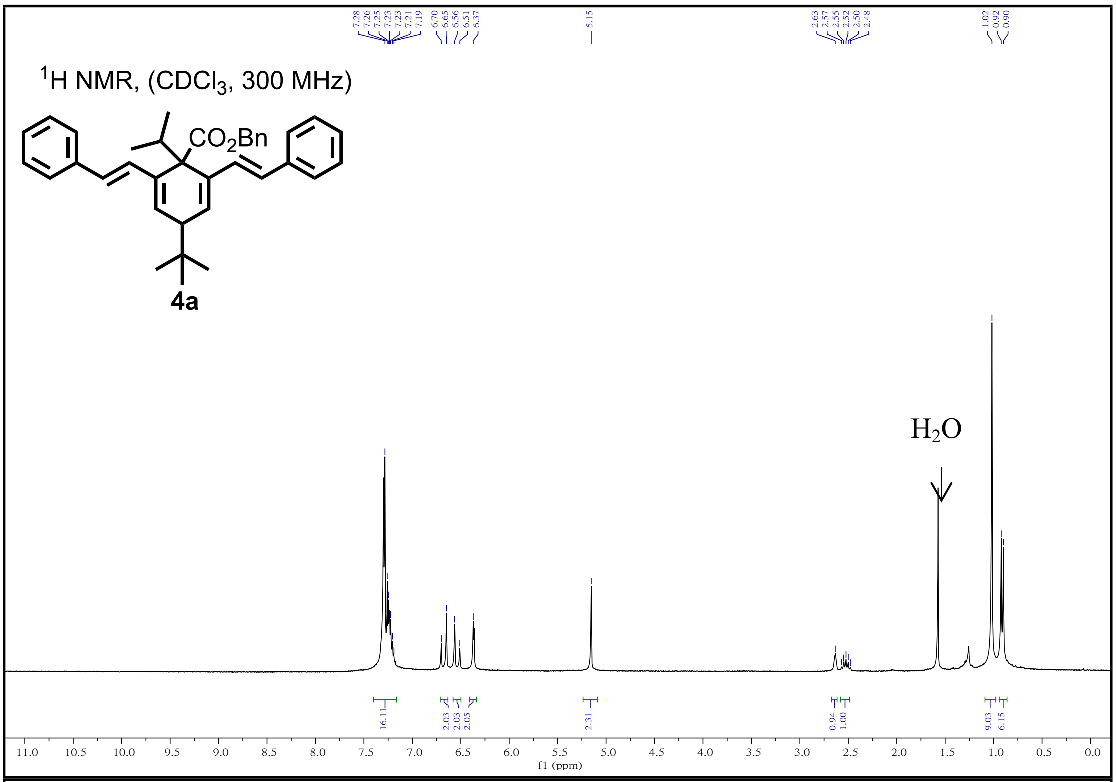

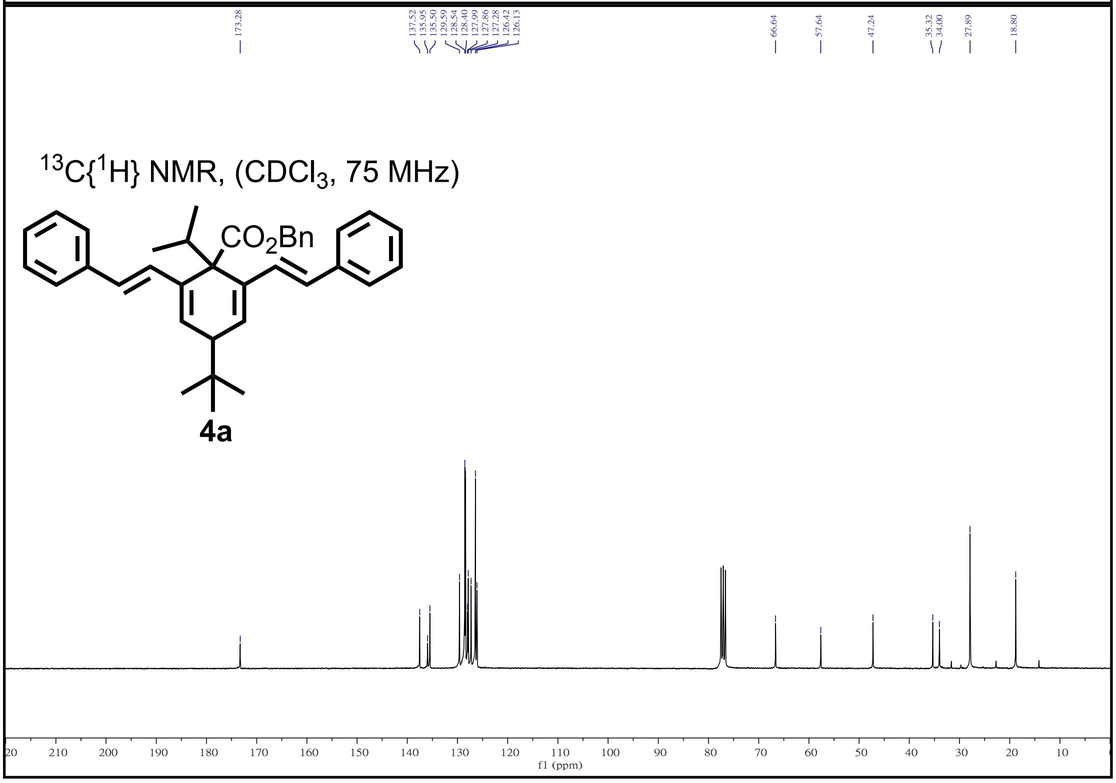

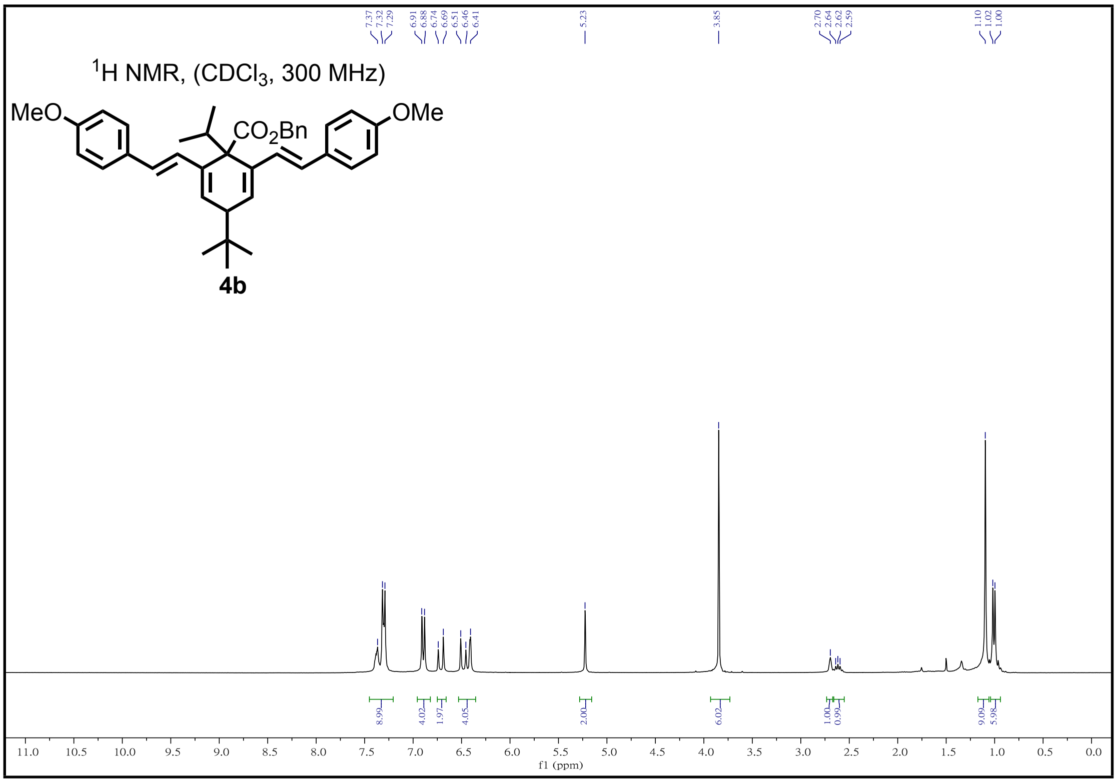
1H NMR, (CDCl3, 300 MHz)
MeO
CO2Bn
OMe
4b
f1 (ppm)

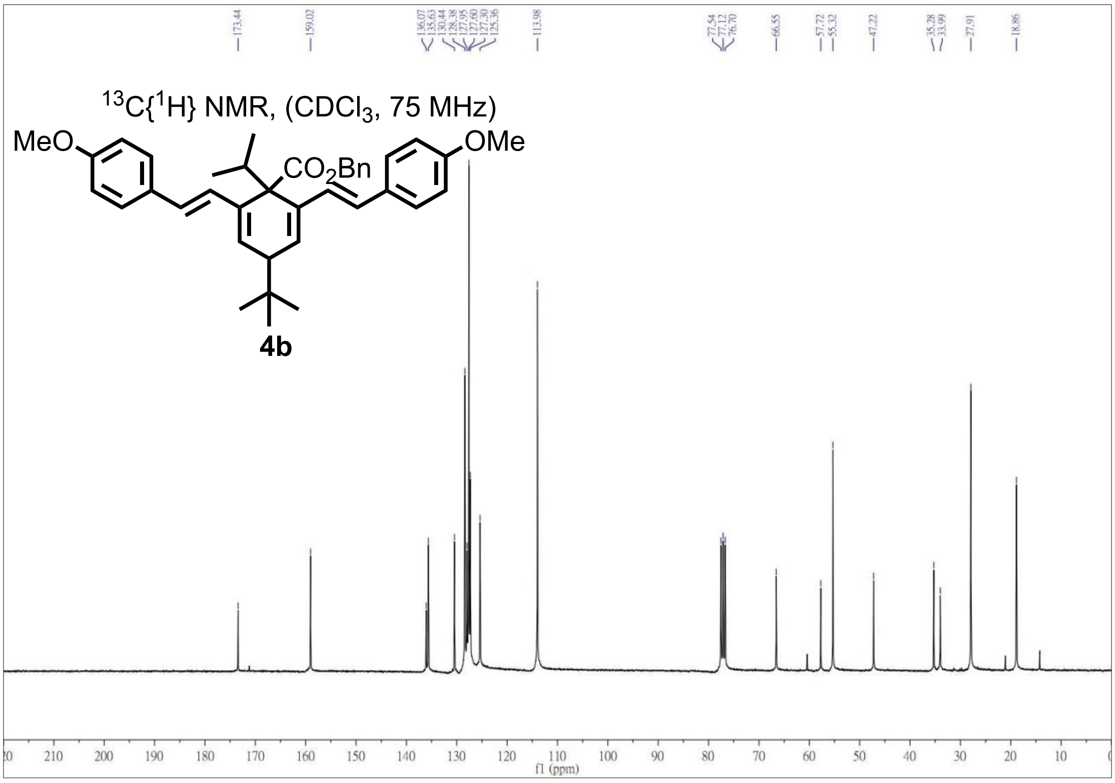
13C{1H} NMR, (CDCl3, 75 MHz)
MeO
CO2Bn
OMe
4b
f1 (ppm)

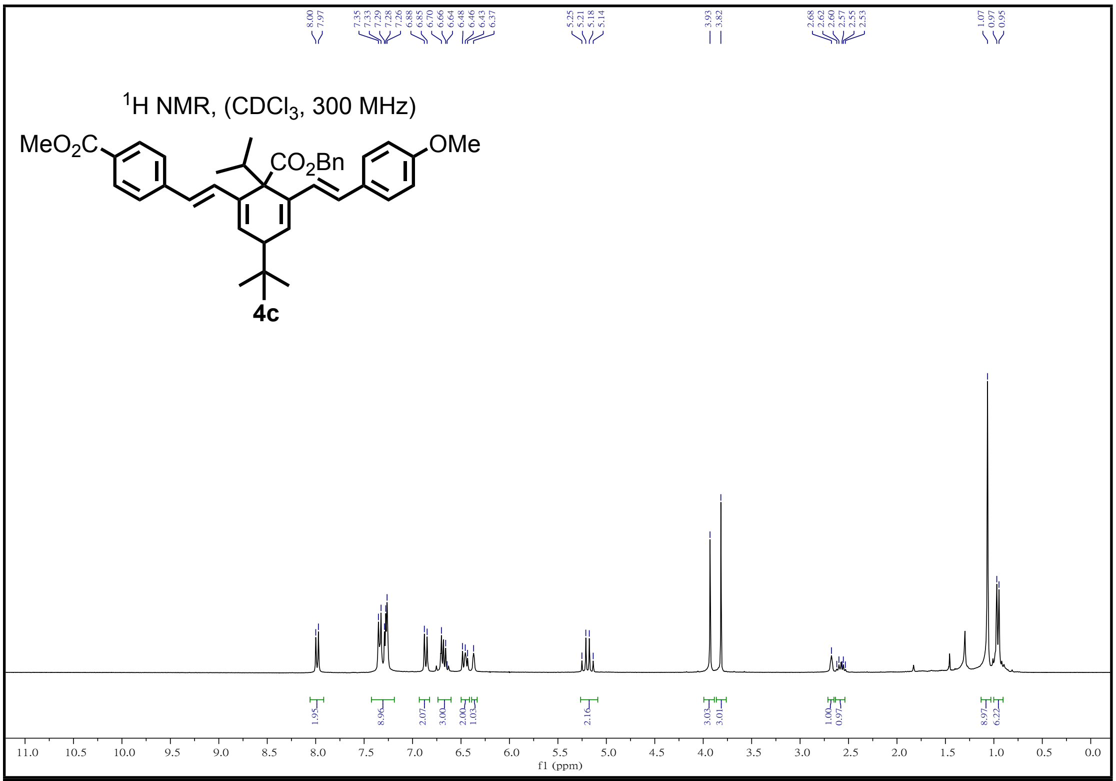
1H NMR, (CDCl3, 300 MHz)
MeO2C
CO2Bn
OMe
4c
f1 (ppm)

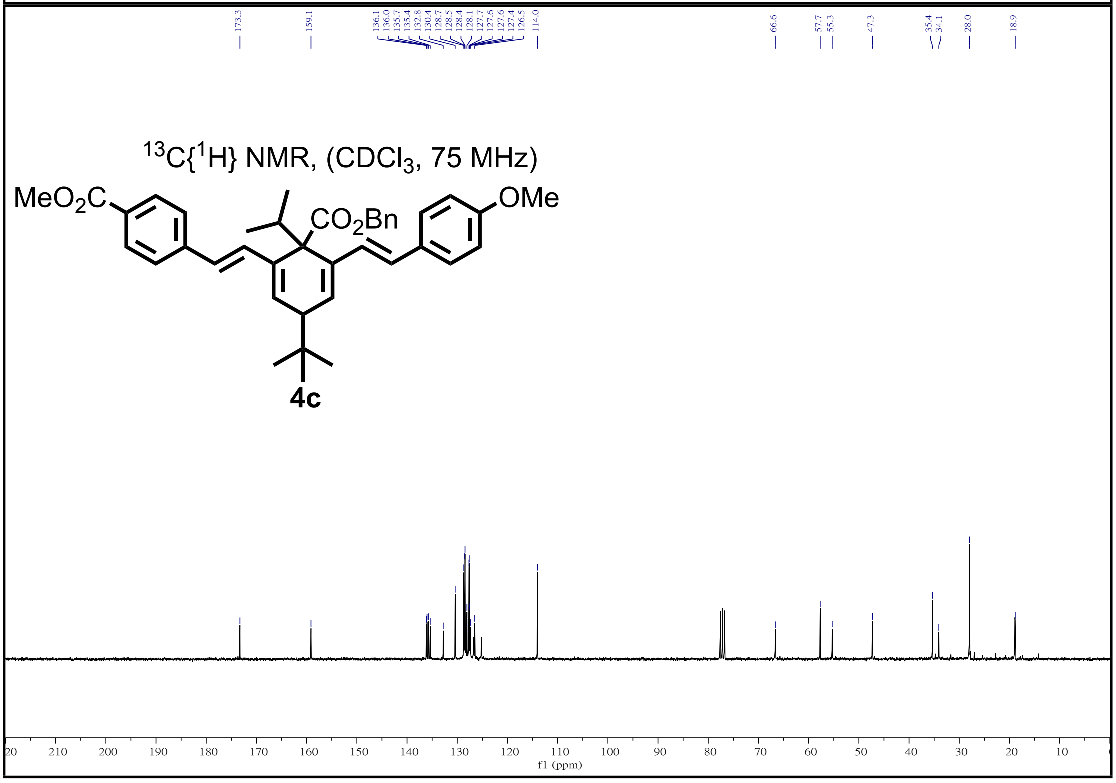
13C{1H} NMR, (CDCl3, 75 MHz)
MeO2C
CO2Bn
OMe
4c
f1 (ppm)

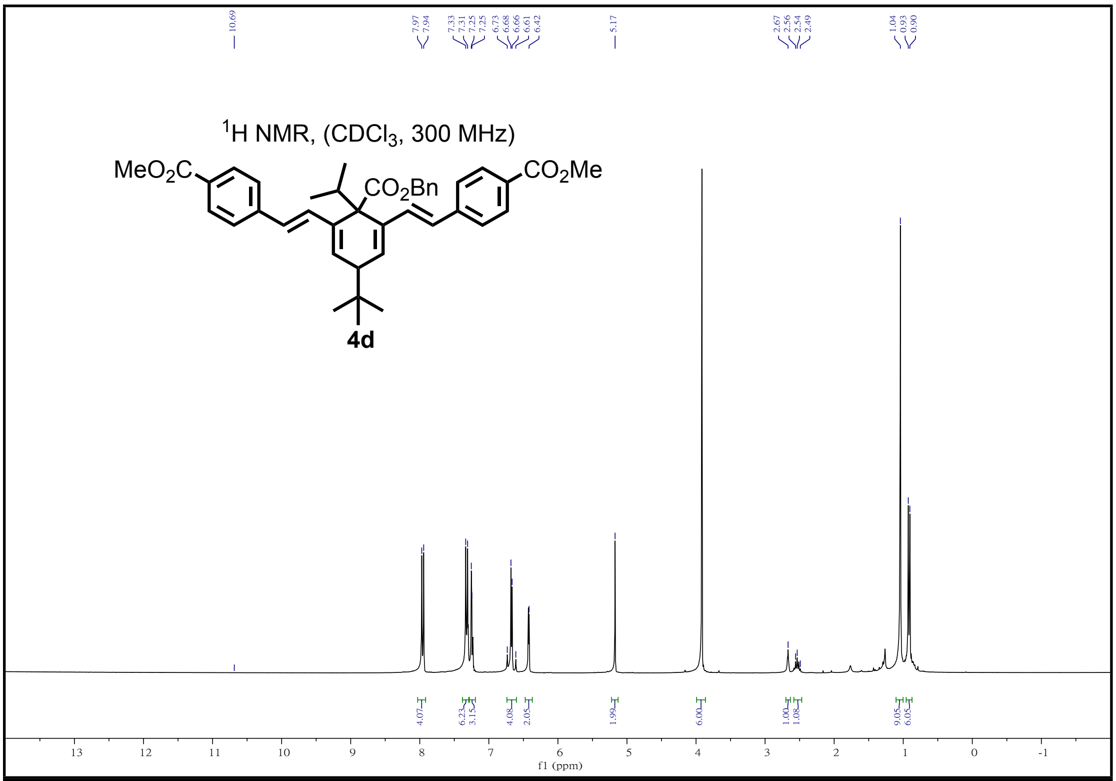
1H NMR, (CDCl3, 300 MHz)
MeO2C
CO2Bn
CO2Me
4d
f1 (ppm)

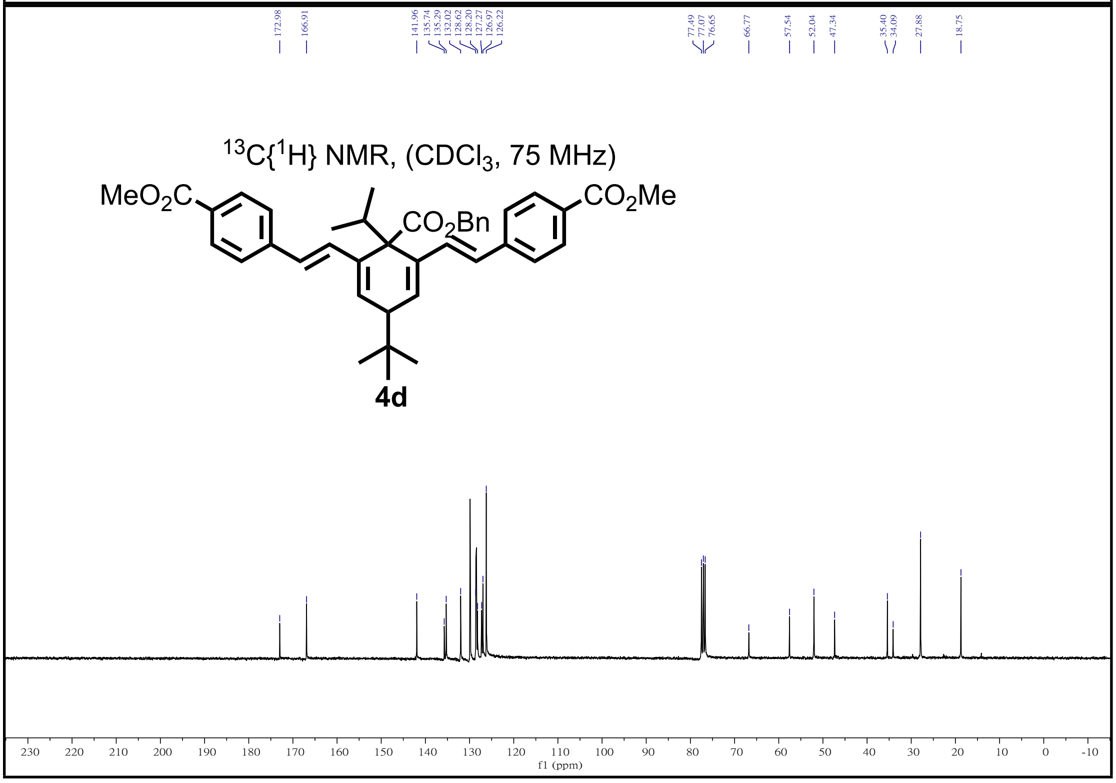
13C{1H} NMR, (CDCl3, 75 MHz)
MeO2C
CO2Bn
CO2Me
4d
f1 (ppm)

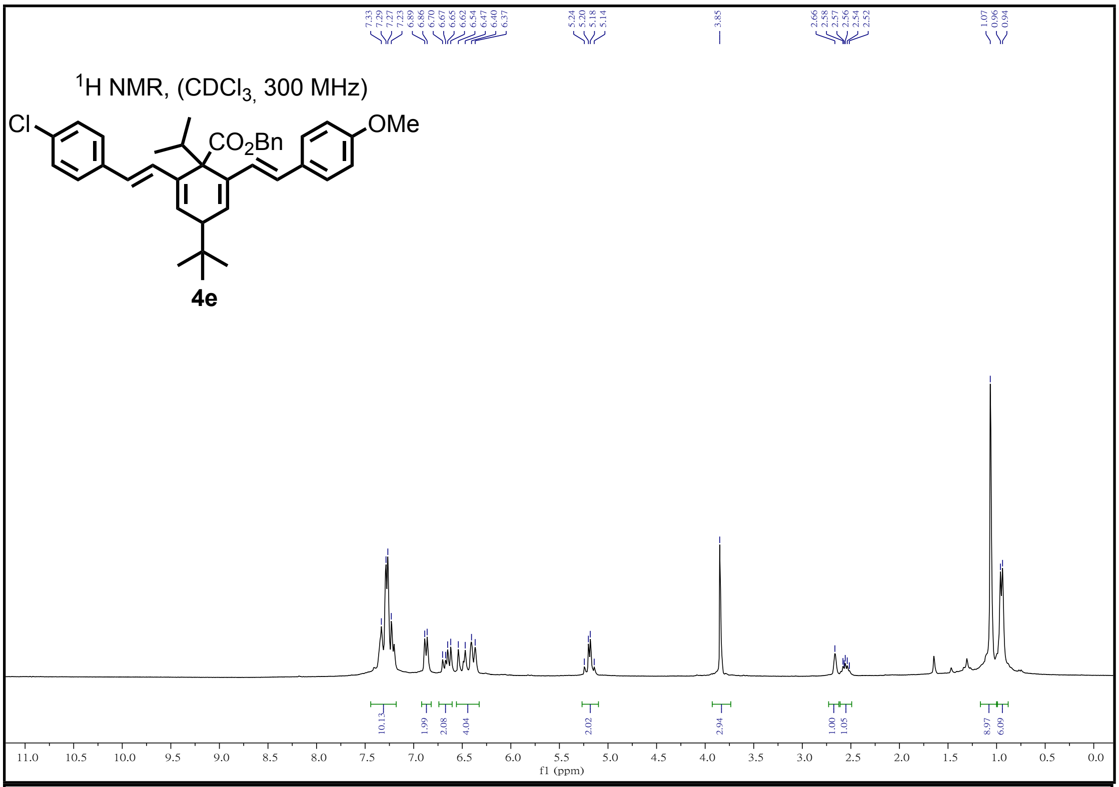

^{1}H NMR, ($CDCl_3$, 300 MHz)
Cl
CO_2Bn
OMe
4e
f1 (ppm)

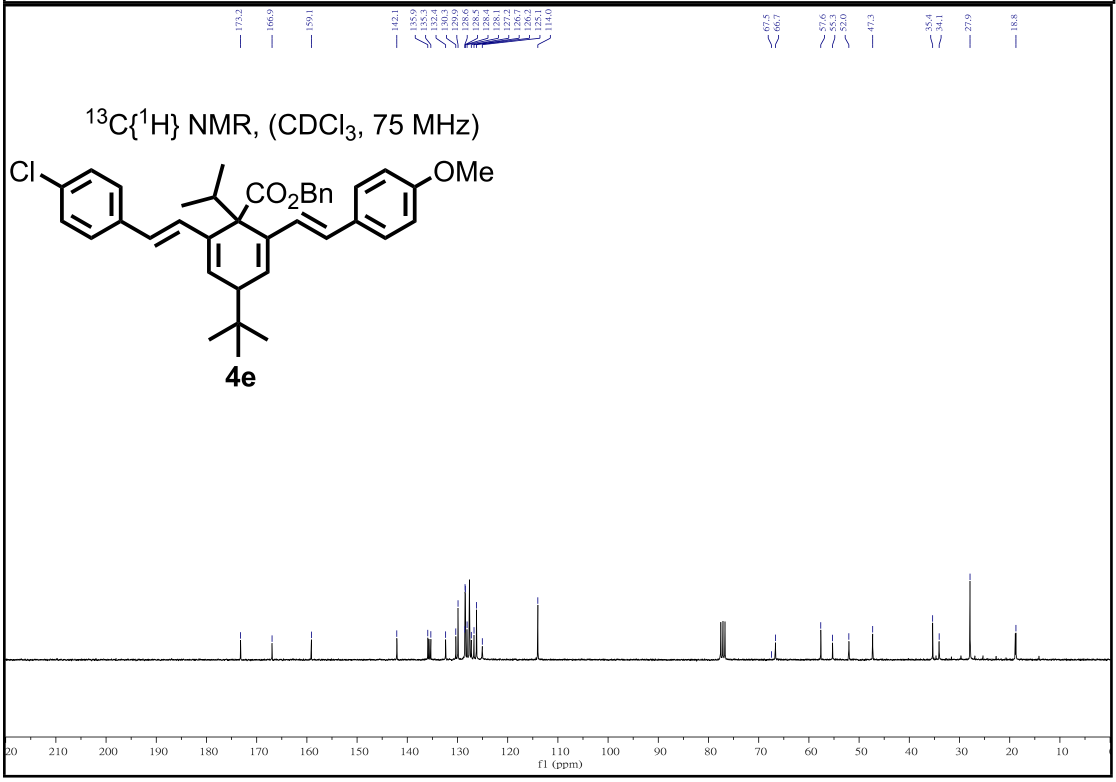

$^{13}C\{^1H\}$ NMR, ($CDCl_3$, 75 MHz)
Cl
CO_2Bn
OMe
4e
f1 (ppm)

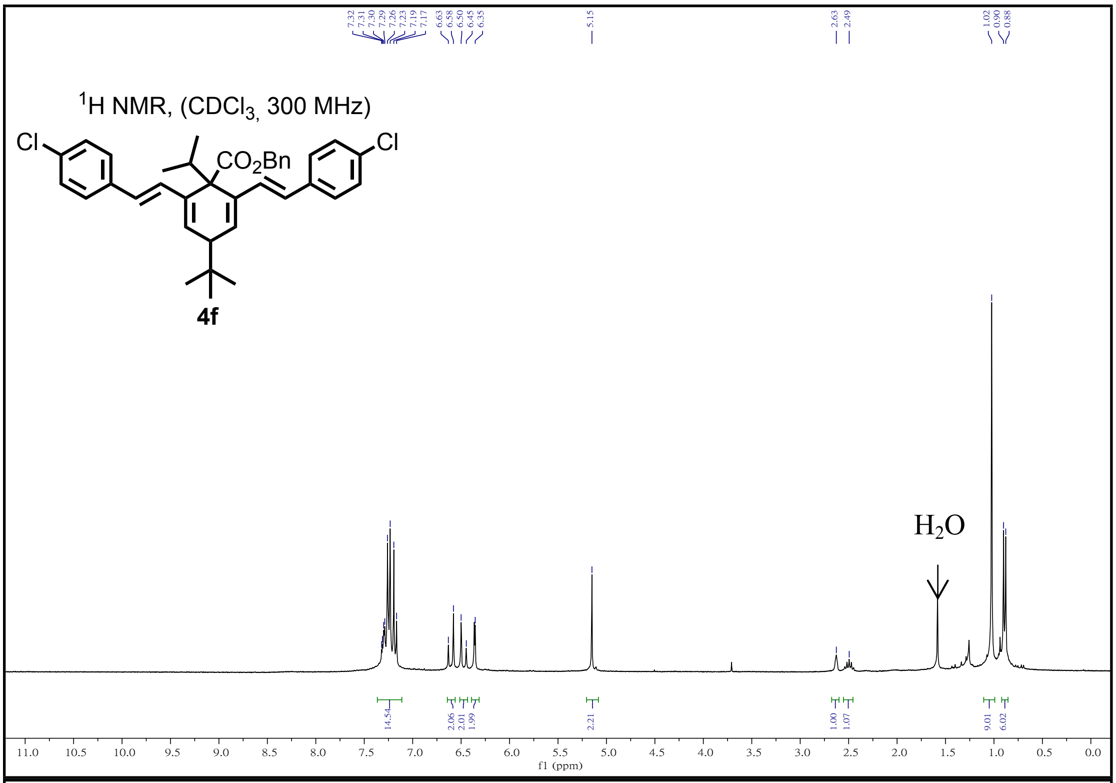
1H NMR, (CDCl3, 300 MHz)
Cl
CO2Bn
Cl
4f
H2O
7.32 7.31 7.30 7.29 7.26 7.23 7.19 7.17 6.63 6.58 6.50 6.45 6.35
5.15
2.63 2.49
1.02 0.90 0.88
14.54 2.06 2.01 1.99 2.21 1.00 1.07 9.01 6.02
f1 (ppm)

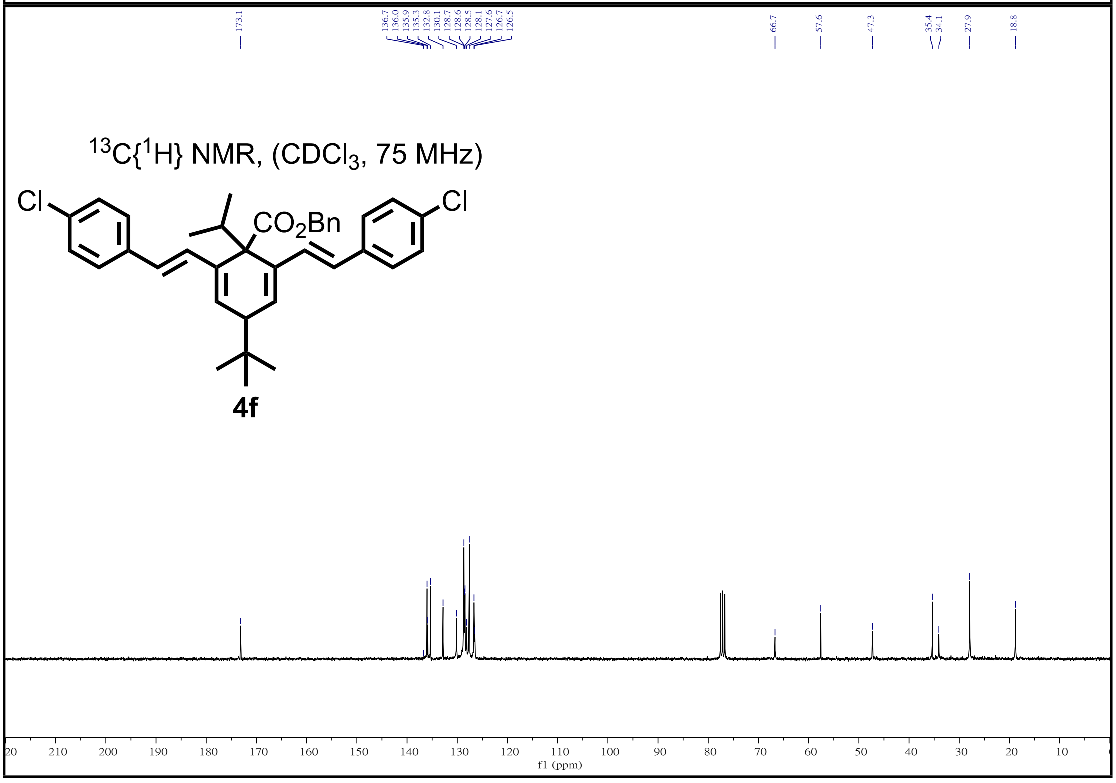
13C{1H} NMR, (CDCl3, 75 MHz)
Cl
CO2Bn
Cl
4f
173.1
136.7 136.0 135.9 135.3 132.8 130.1 128.7 128.6 128.5 128.1 127.6 126.7 126.5
66.7 57.6 47.3 35.4 34.1 27.9 18.8
f1 (ppm)

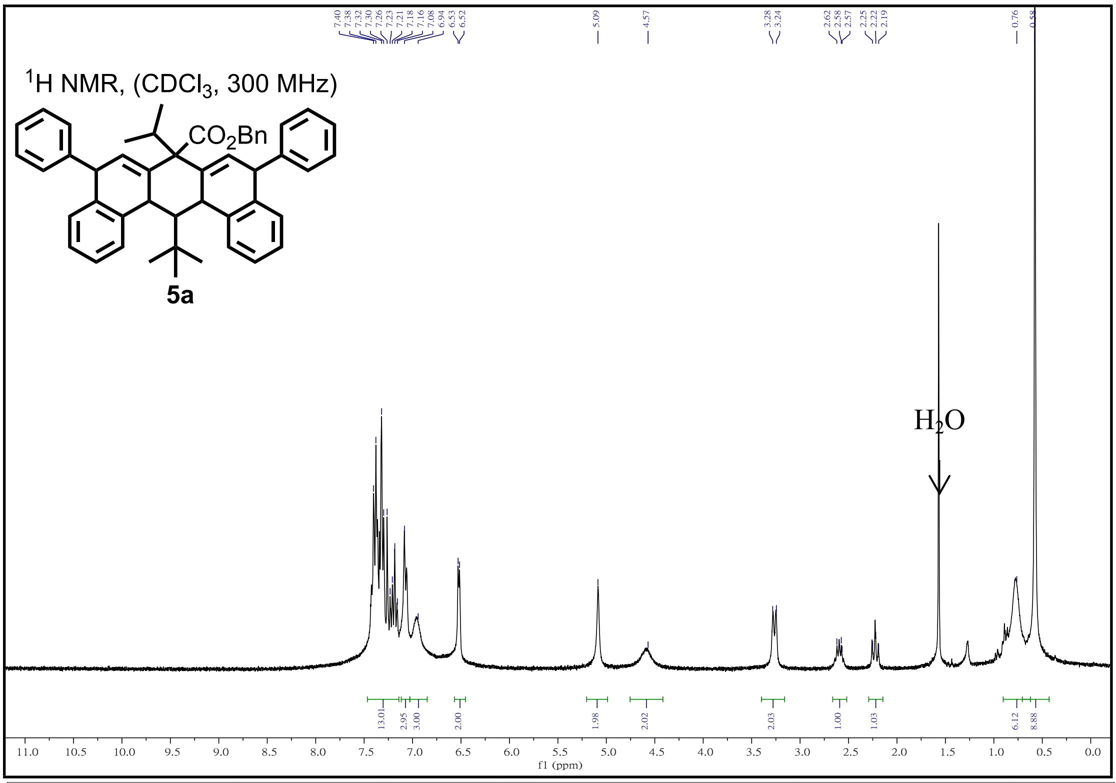
^{1}H NMR, (CDCl$_3$, 300 MHz)
CO$_2$Bn
5a
H$_2$O
f1 (ppm)

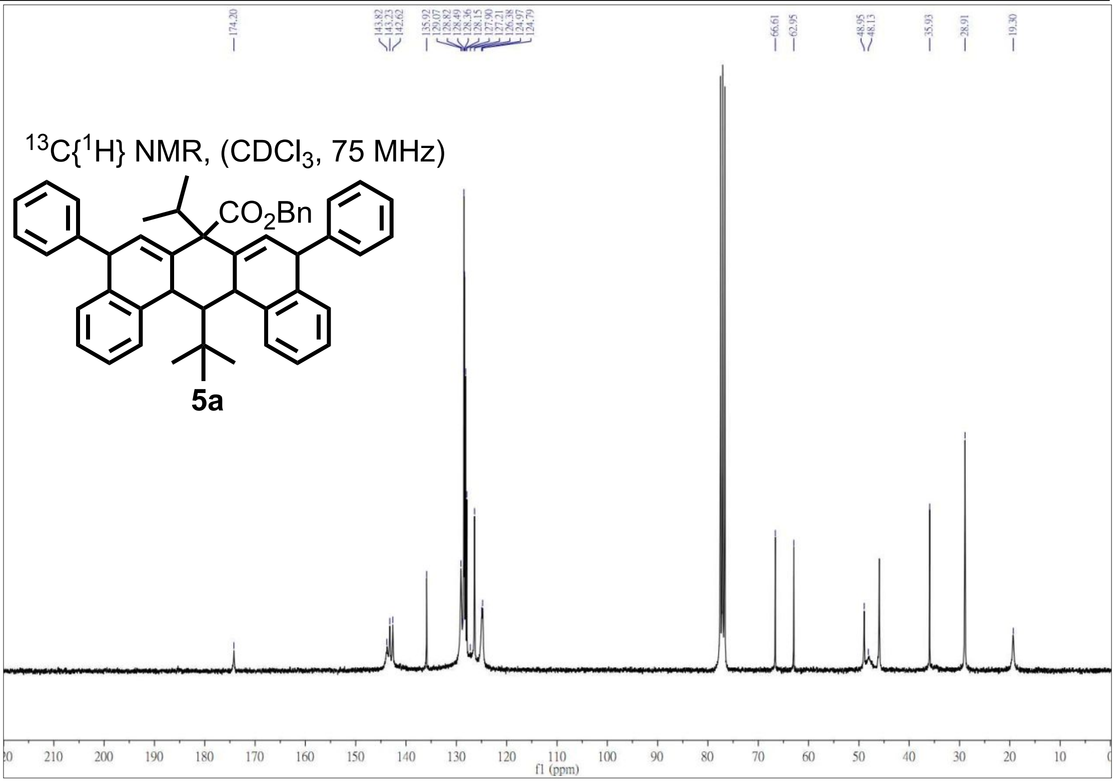
^{13}C{^{1}H} NMR, (CDCl$_3$, 75 MHz)
CO$_2$Bn
5a
f1 (ppm)

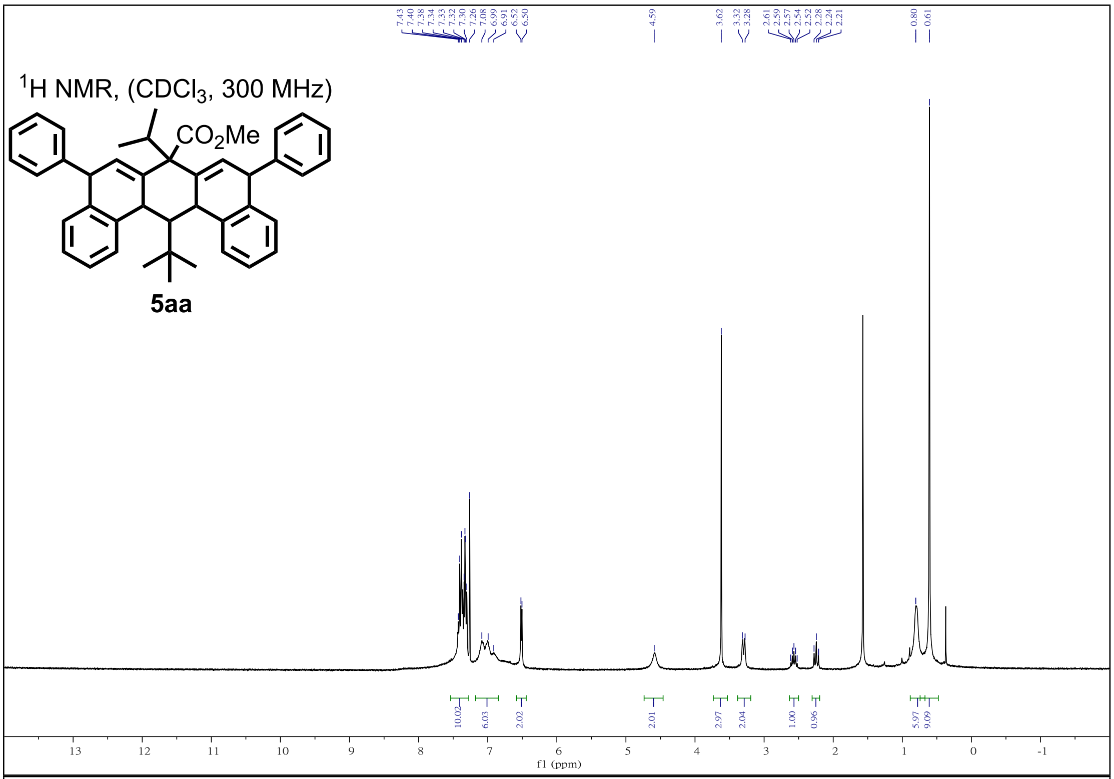

1H NMR, (CDCl3, 300 MHz)
CO2Me
5aa
f1 (ppm)

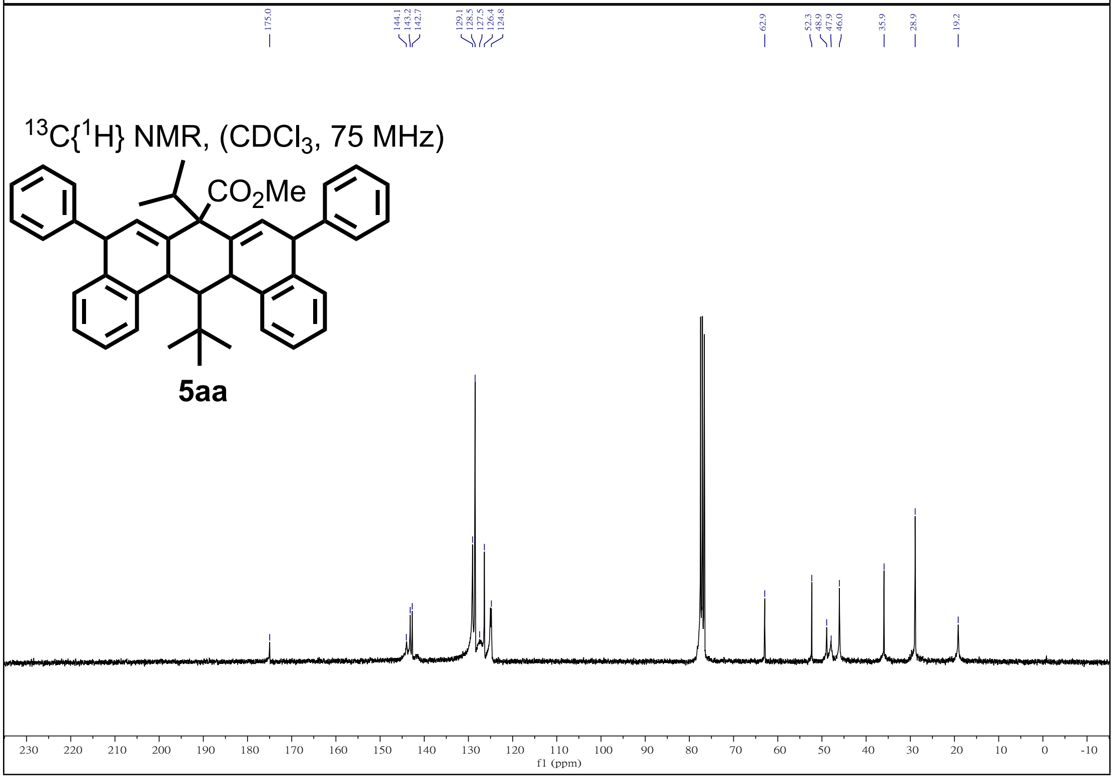

13C{1H} NMR, (CDCl3, 75 MHz)
CO2Me
5aa
f1 (ppm)

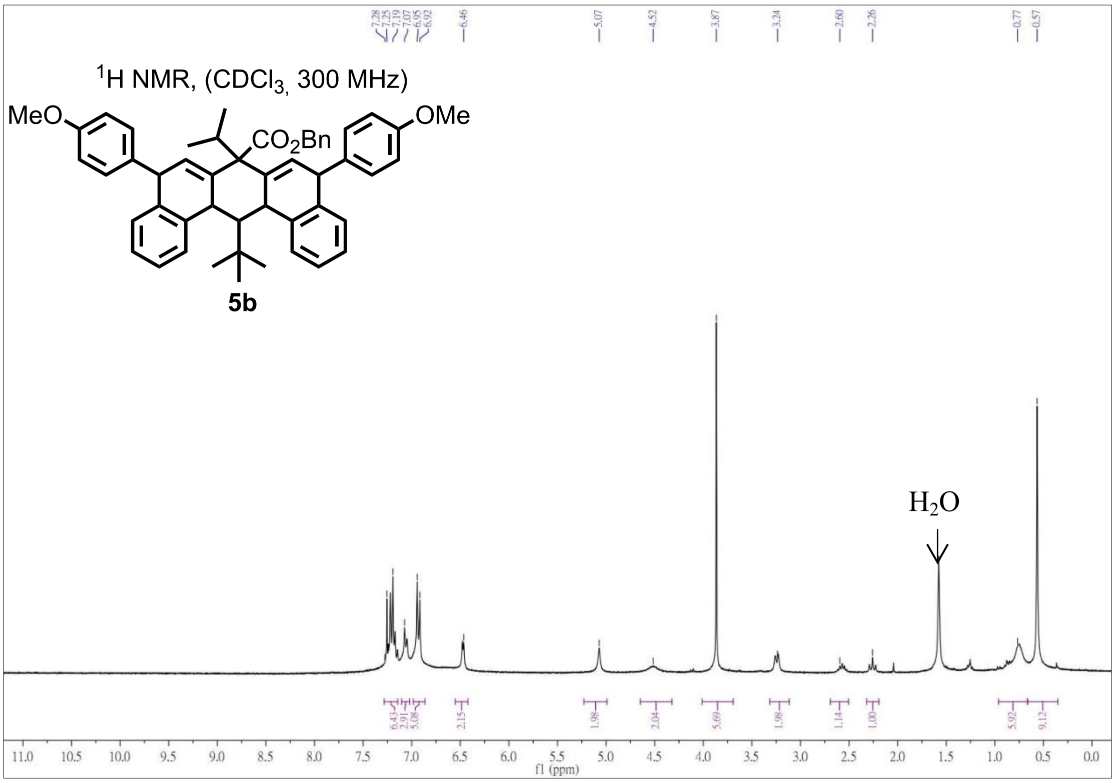

^{1}H NMR, (CDCl$_3$, 300 MHz)
MeO
OMe
CO$_2$Bn
5b
H$_2$O
f1 (ppm)

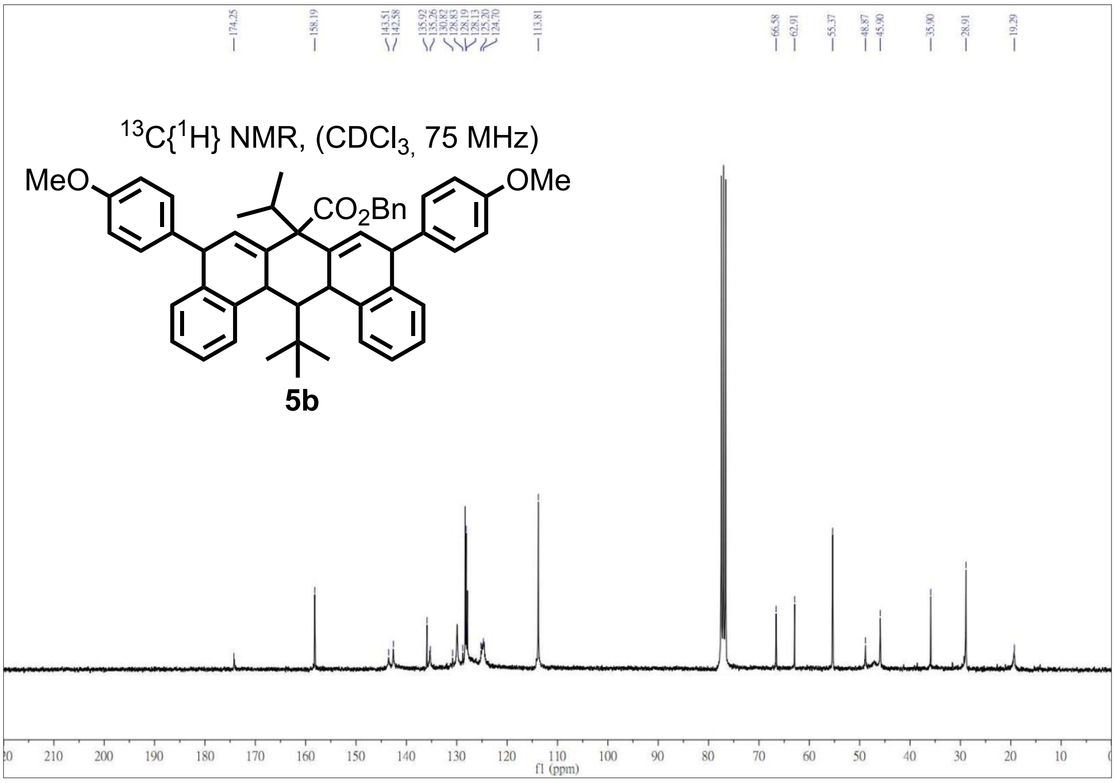

^{13}C{^{1}H} NMR, (CDCl$_3$, 75 MHz)
MeO
OMe
CO$_2$Bn
5b
f1 (ppm)

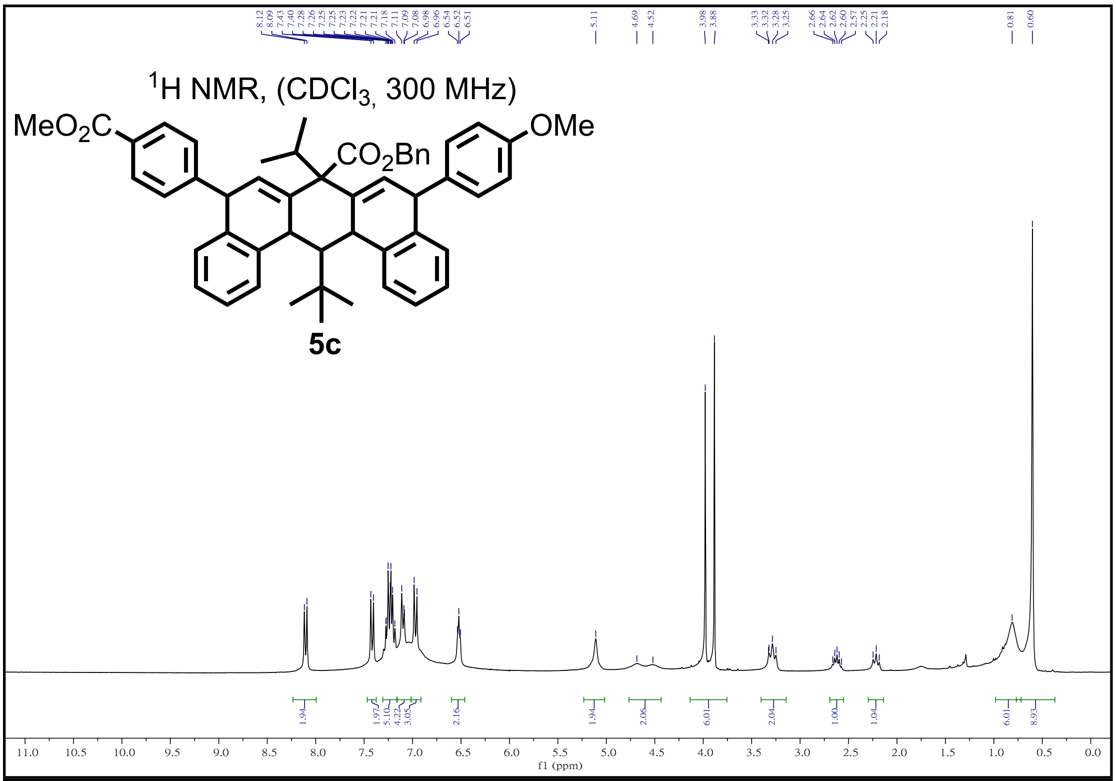
1H NMR, (CDCl3, 300 MHz)
MeO2C
OMe
CO2Bn
5c
f1 (ppm)

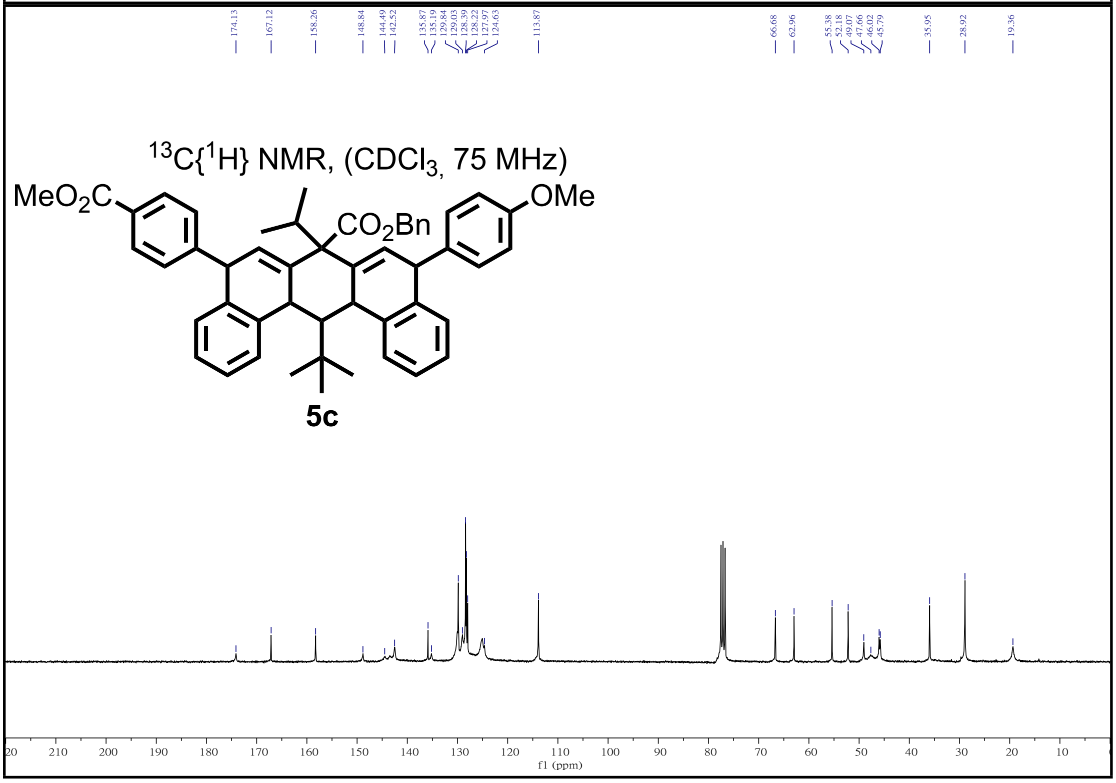
13C{1H} NMR, (CDCl3, 75 MHz)
MeO2C
OMe
CO2Bn
5c
f1 (ppm)

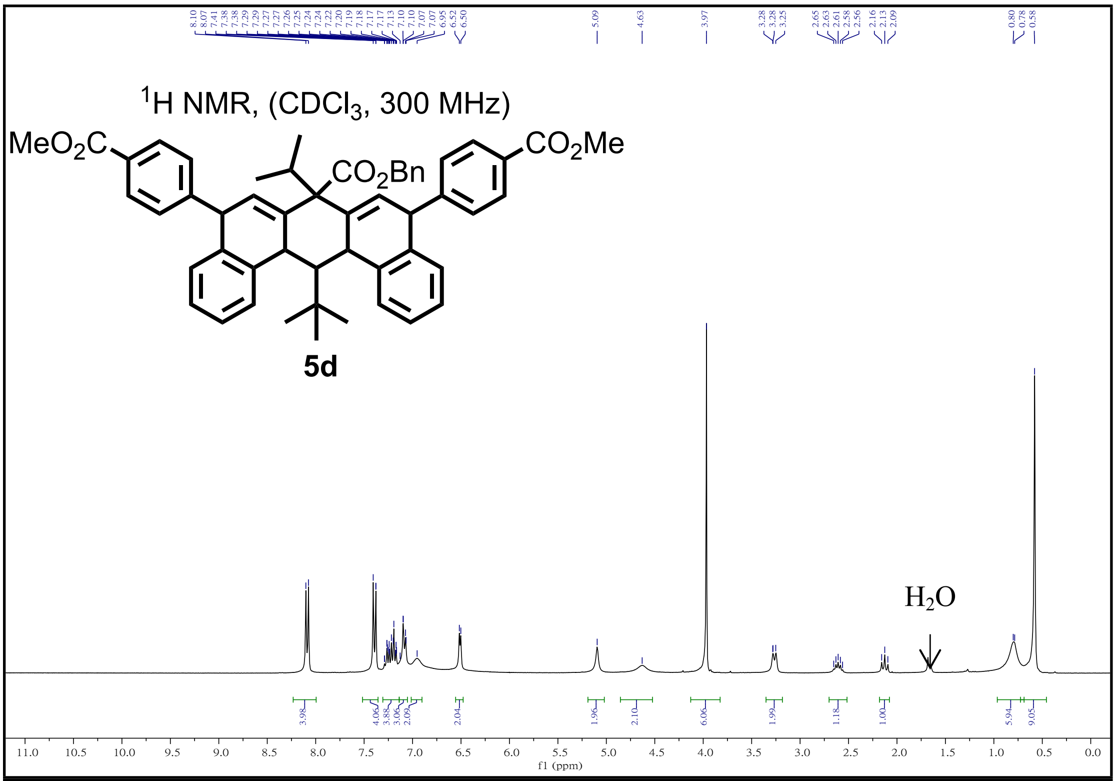
^{1}H NMR, ($CDCl_3$, 300 MHz)
MeO_2C
CO_2Bn
CO_2Me
5d
H_2O
f1 (ppm)

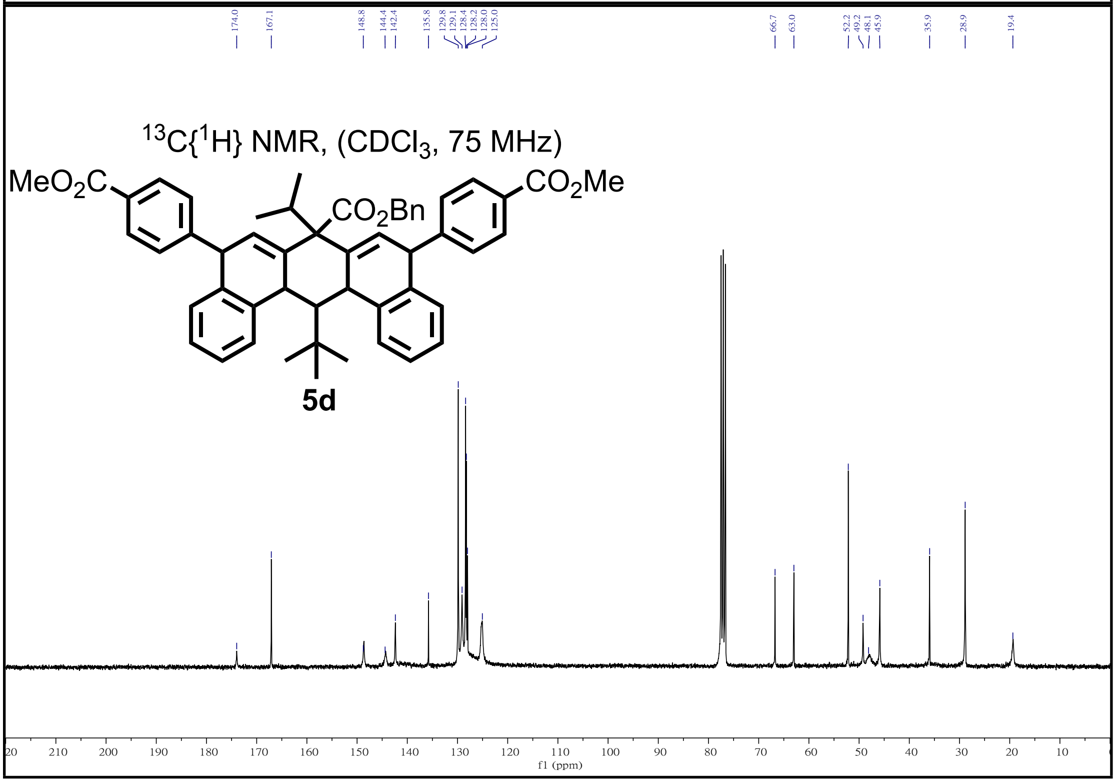
$^{13}C\{^{1}H\}$ NMR, ($CDCl_3$, 75 MHz)
MeO_2C
CO_2Bn
CO_2Me
5d
f1 (ppm)

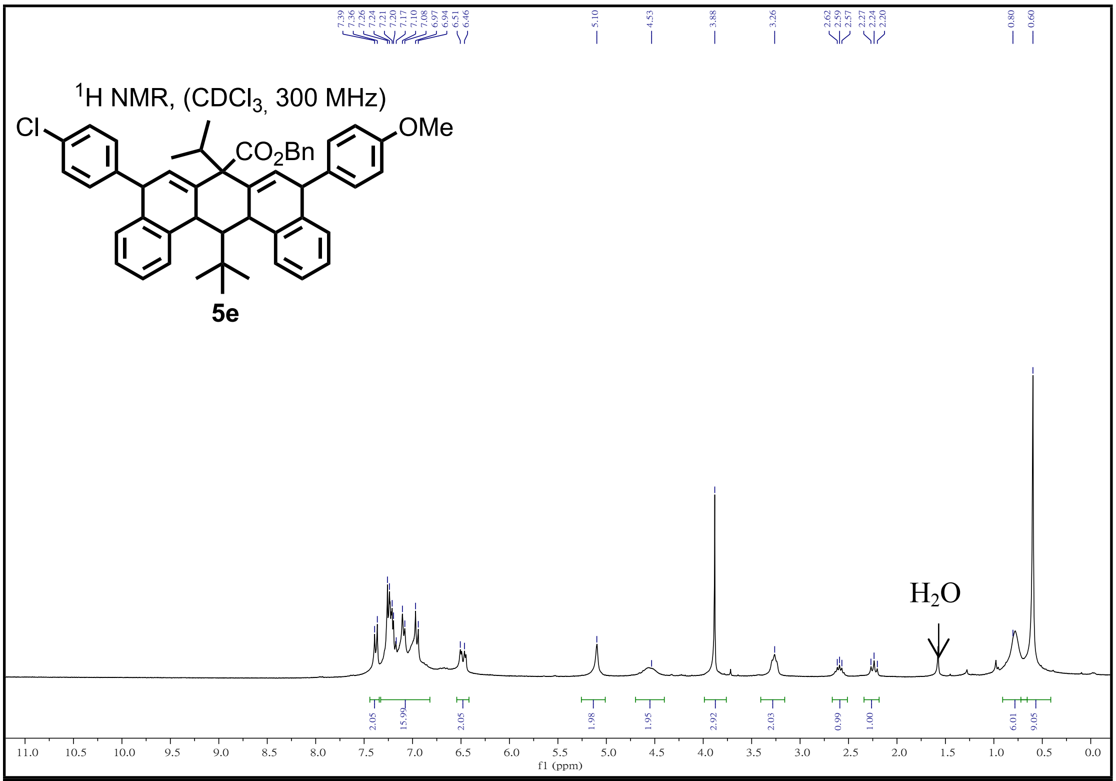
1H NMR, (CDCl3, 300 MHz)
Cl
CO2Bn
OMe
5e
H2O
7.39 7.36 7.26 7.24 7.21 7.20 7.17 7.10 7.08 6.97 6.94 6.51 6.46
5.10
4.53
3.88
3.26
2.62 2.59 2.57 2.27 2.24 2.20
0.80
0.60
2.05 15.99 2.05 1.98 1.95 2.92 2.03 0.99 1.00 6.01 9.05
f1 (ppm)

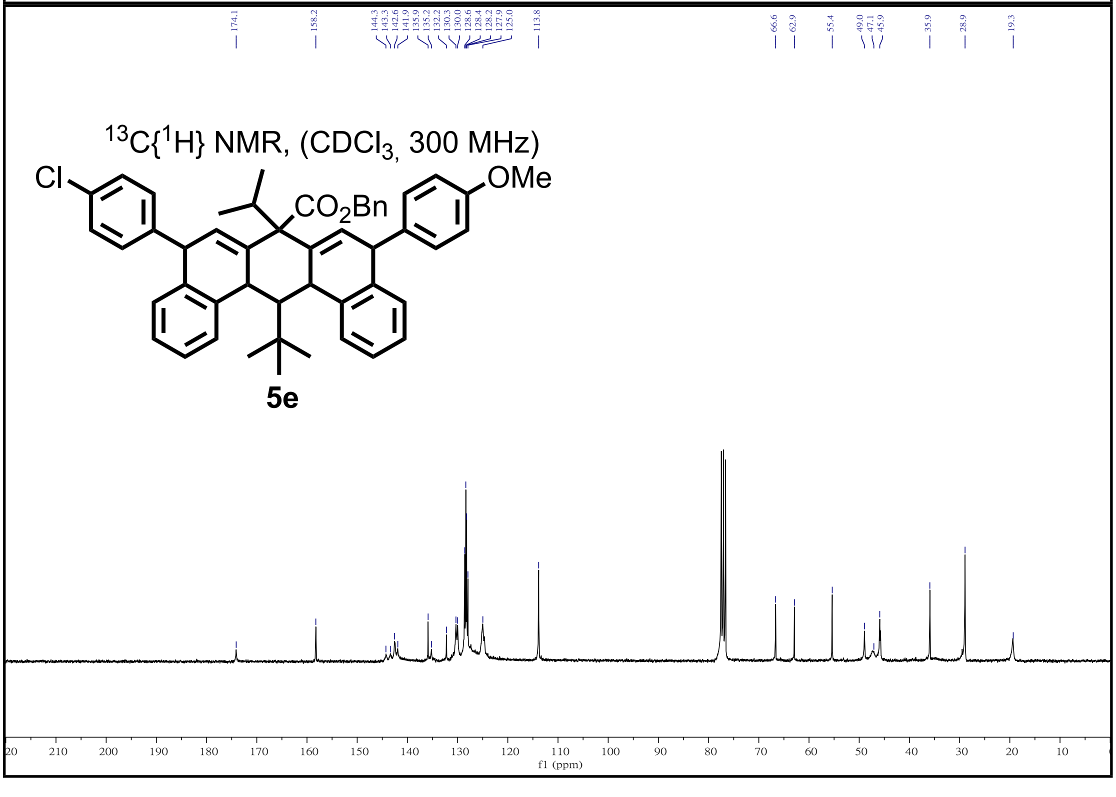
13C{1H} NMR, (CDCl3, 300 MHz)
Cl
CO2Bn
OMe
5e
174.1
158.2
144.3 143.3 142.6 141.9 135.9 135.2 132.2 130.3 130.0 128.6 128.4 128.2 127.9 125.0
113.8
66.6 62.9 55.4 49.0 47.1 45.9 35.9 28.9 19.3
f1 (ppm)

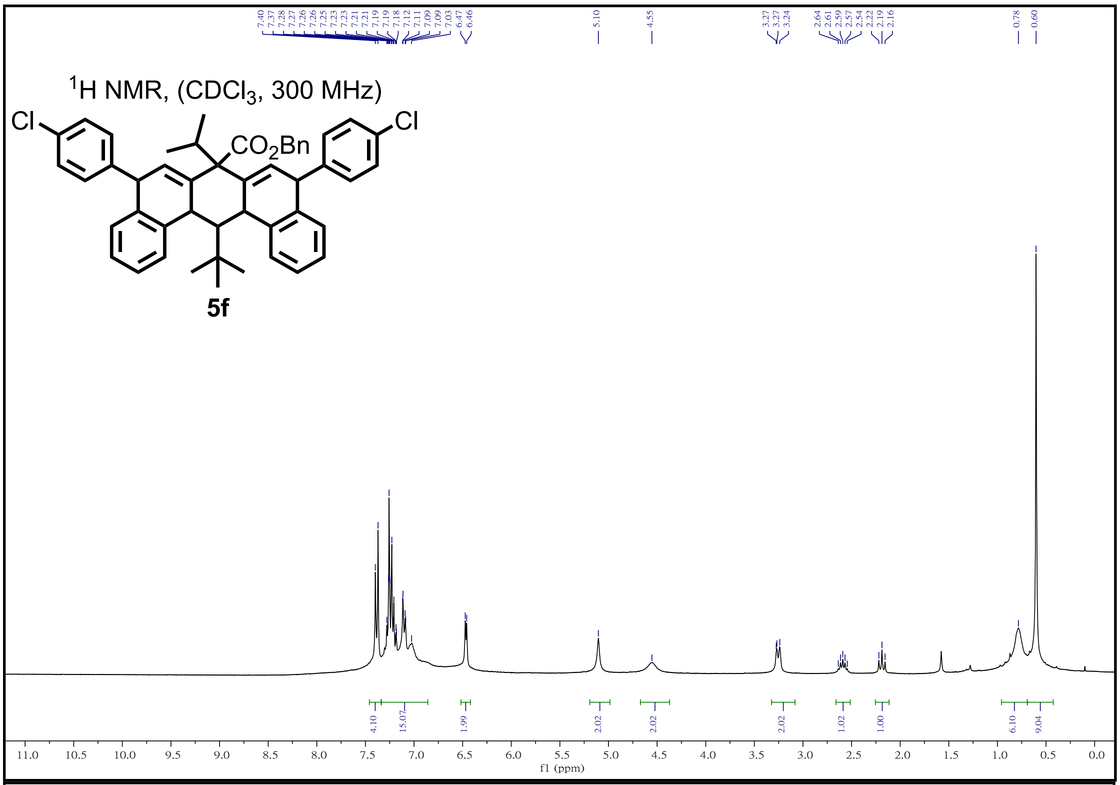

^{1}H NMR, ($CDCl_3$, 300 MHz)
5f
f1 (ppm)

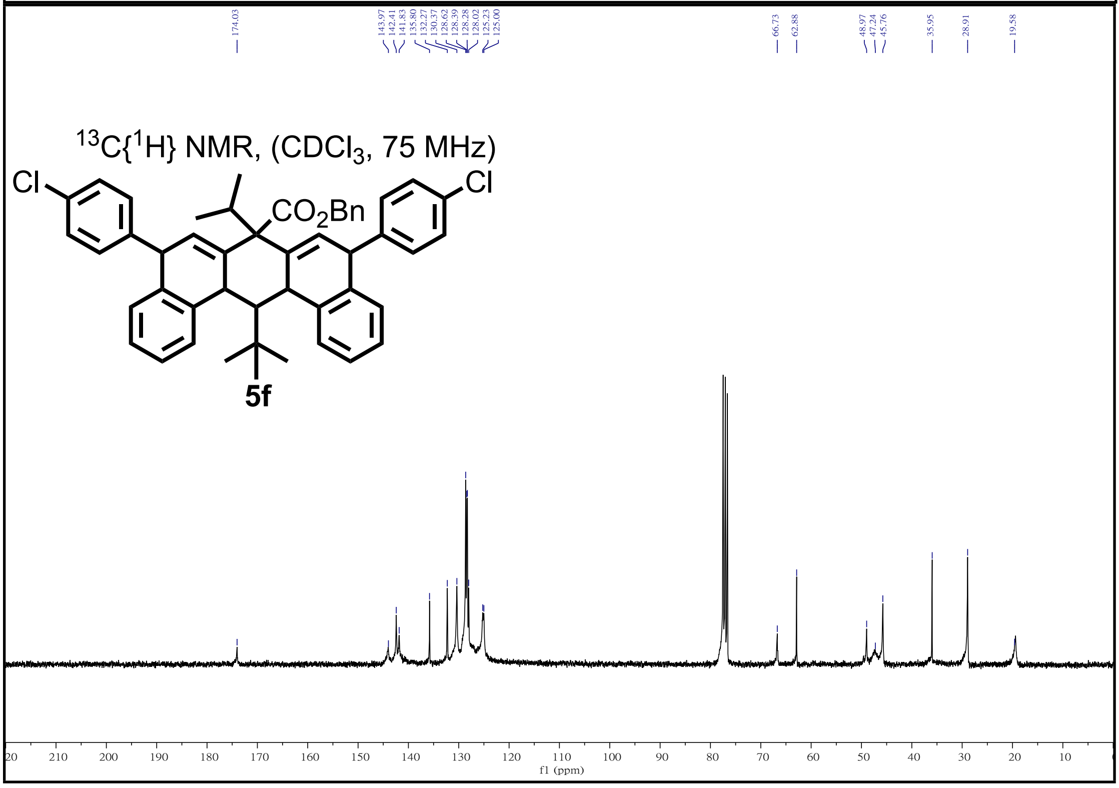

$^{13}C\{^1H\}$ NMR, ($CDCl_3$, 75 MHz)
5f
f1 (ppm)

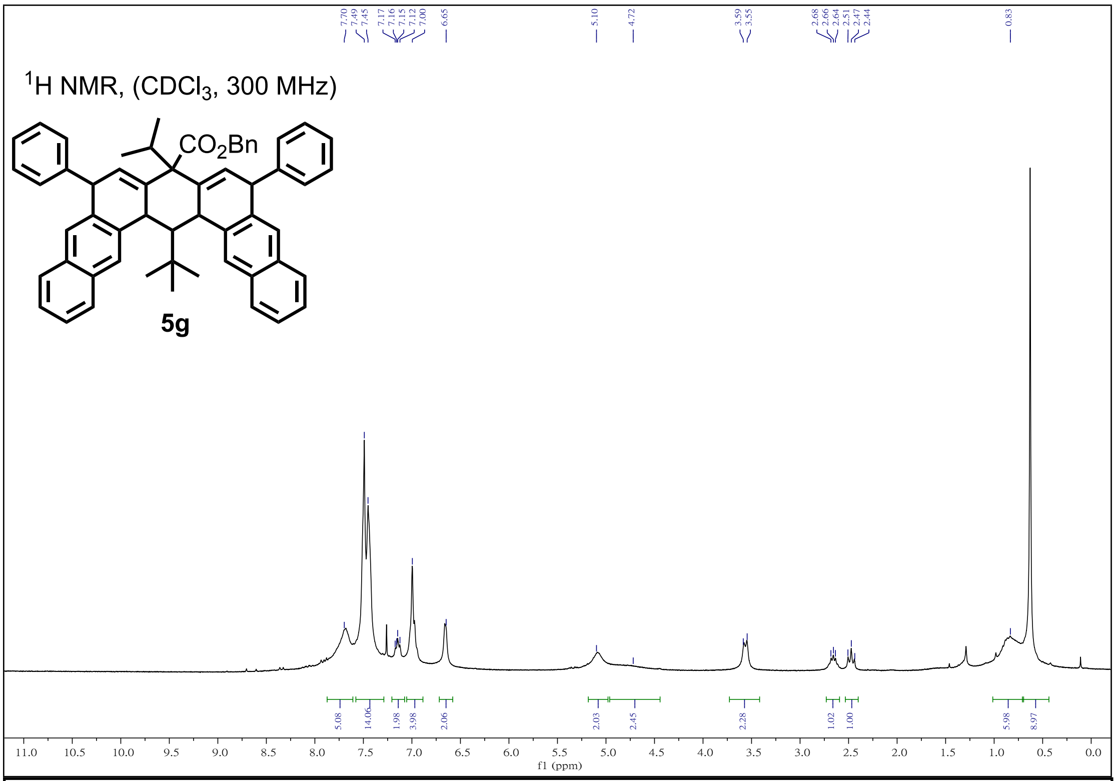
7.70 7.49 7.45 7.17 7.16 7.15 7.12 7.00 6.65 5.10 4.72 3.59 3.55 2.68 2.66 2.64 2.51 2.47 2.44 0.83
^{1}H NMR, ($CDCl_3$, 300 MHz)
CO_2Bn
5g
5.08 14.06 1.98 3.98 2.06 2.03 2.45 2.28 1.02 1.00 5.98 8.97
f1 (ppm)

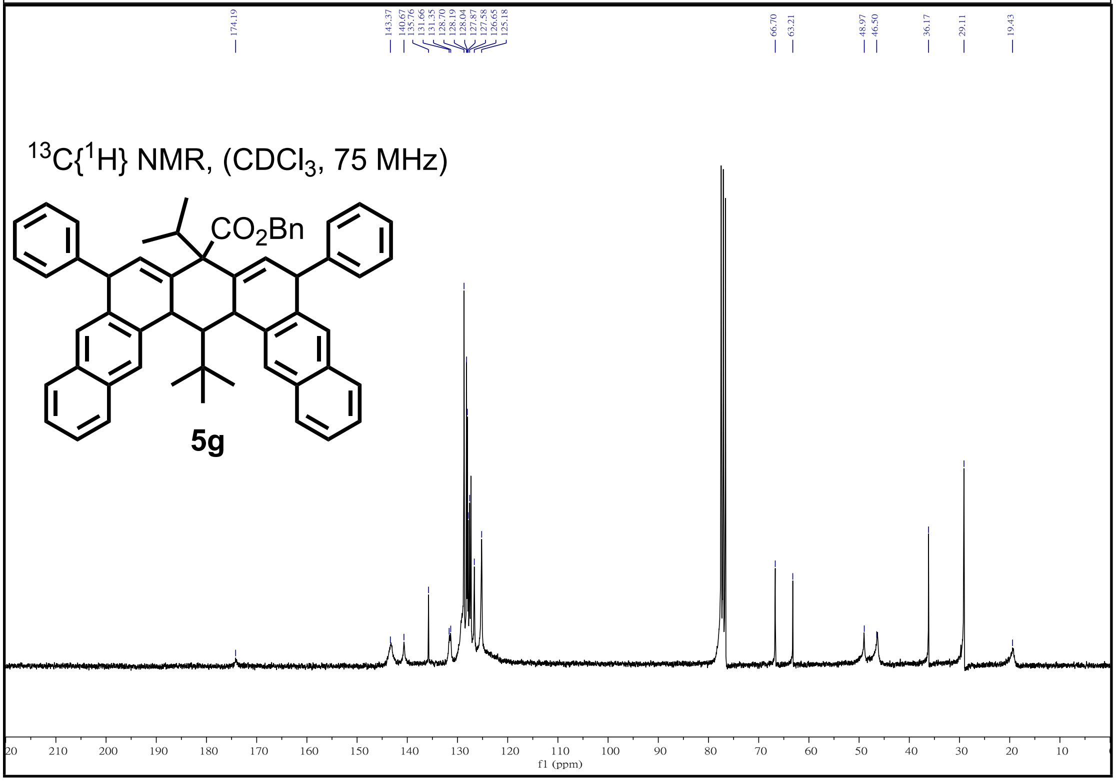
174.19 143.37 140.67 135.76 131.66 131.35 128.70 128.19 128.04 127.87 127.58 126.65 125.18 66.70 63.21 48.97 46.50 36.17 29.11 19.43
$^{13}C\{^1H\}$ NMR, ($CDCl_3$, 75 MHz)
CO_2Bn
5g
f1 (ppm)

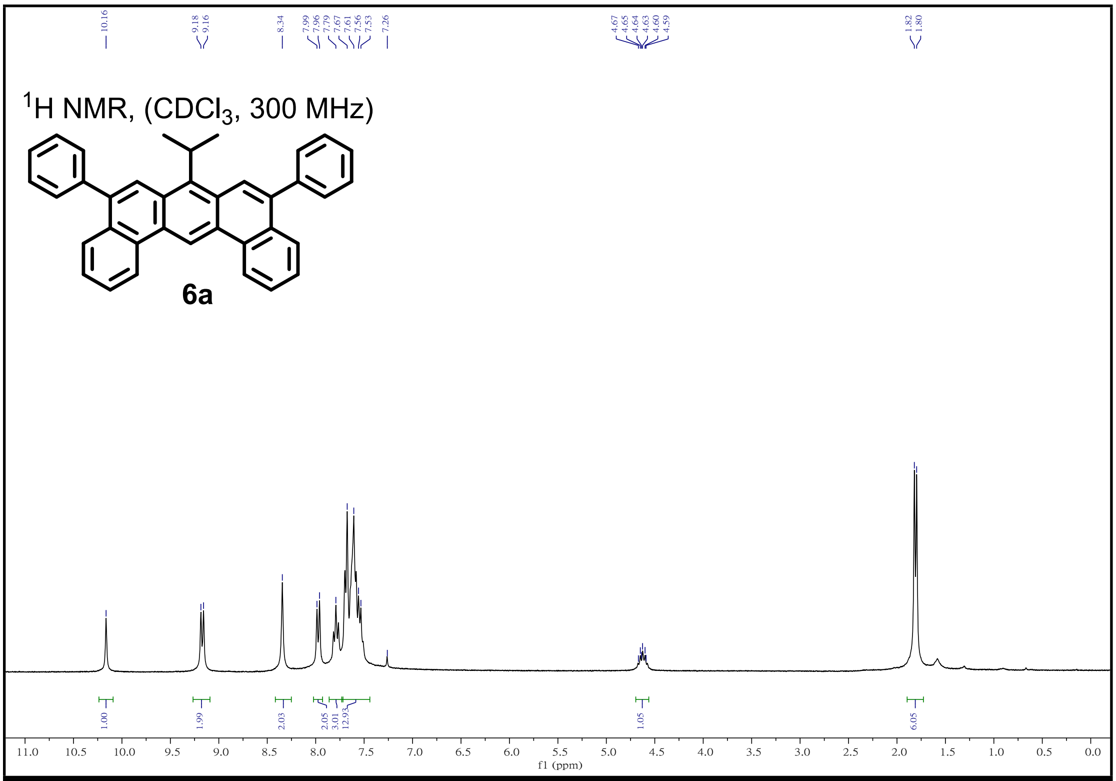

1H NMR, (CDCl3, 300 MHz)
6a
10.16
9.18
9.16
8.34
7.99
7.96
7.79
7.67
7.61
7.56
7.53
7.26
4.67
4.65
4.64
4.63
4.60
4.59
1.82
1.80
1.00
1.99
2.03
2.05
3.01
12.93
1.05
6.05
f1 (ppm)

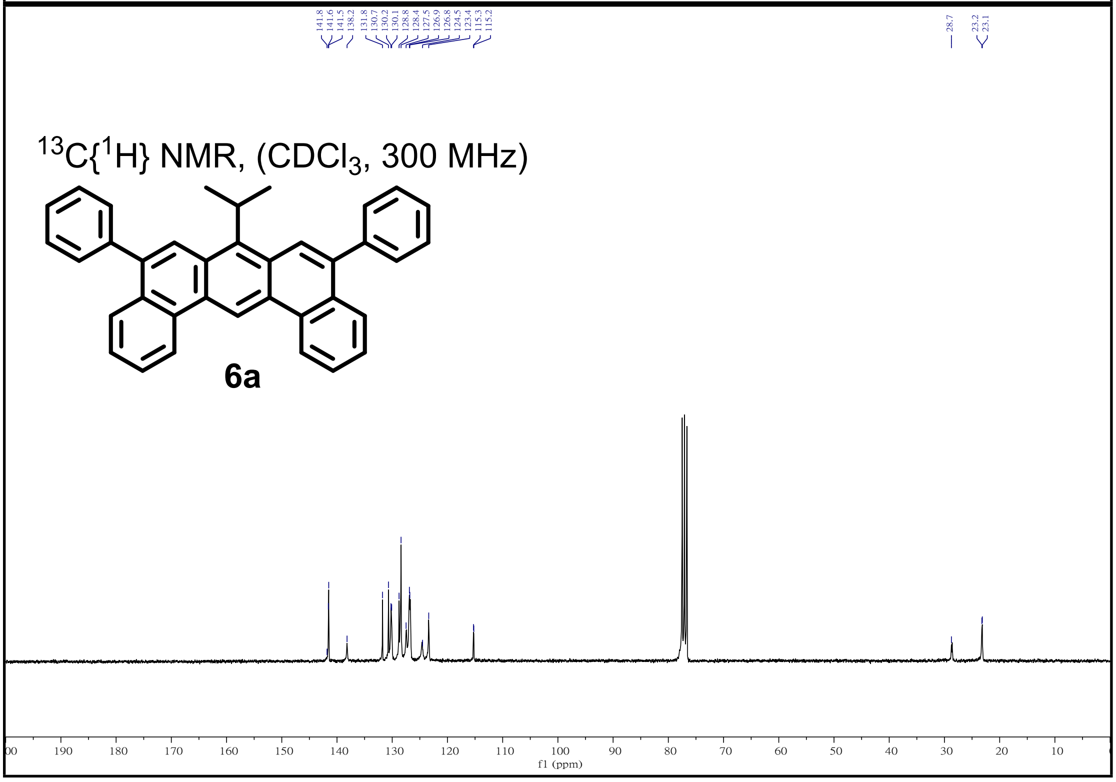

13C{1H} NMR, (CDCl3, 300 MHz)
6a
141.8
141.6
141.5
138.2
131.8
130.7
130.2
130.1
128.8
128.4
127.5
126.9
126.8
124.5
123.4
115.3
115.2
28.7
23.2
23.1
f1 (ppm)

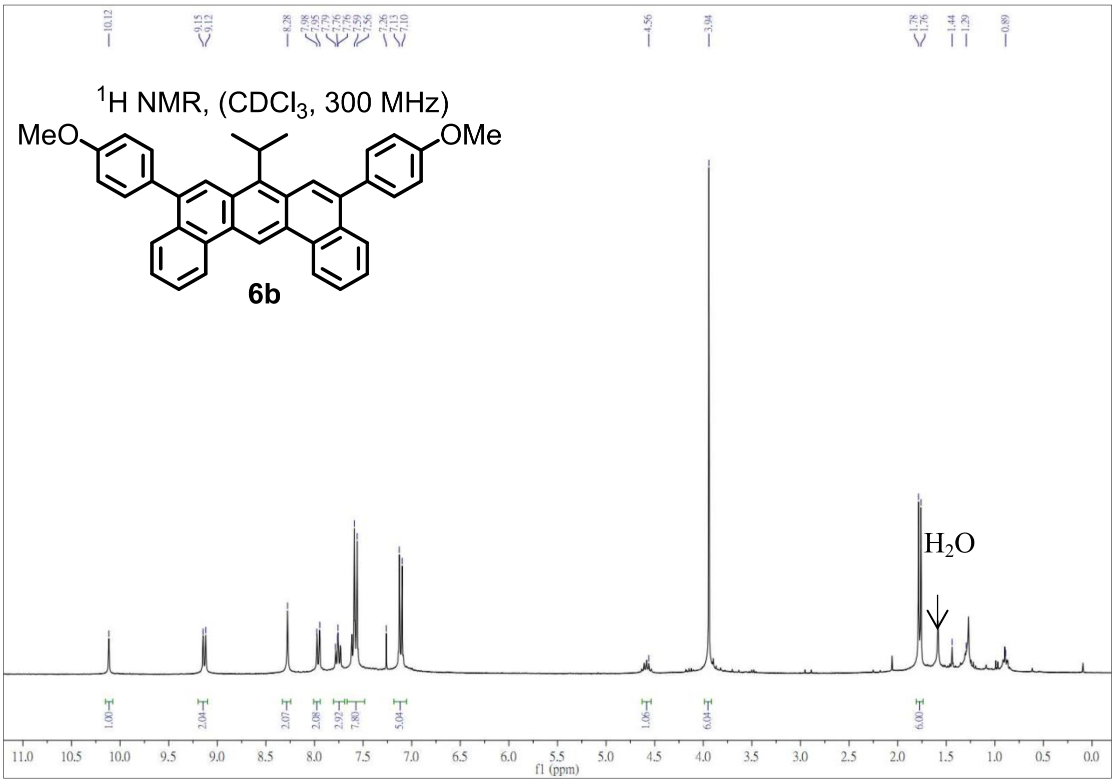

^{1}H NMR, (CDCl$_3$, 300 MHz)
MeO
OMe
6b
H$_2$O
f1 (ppm)

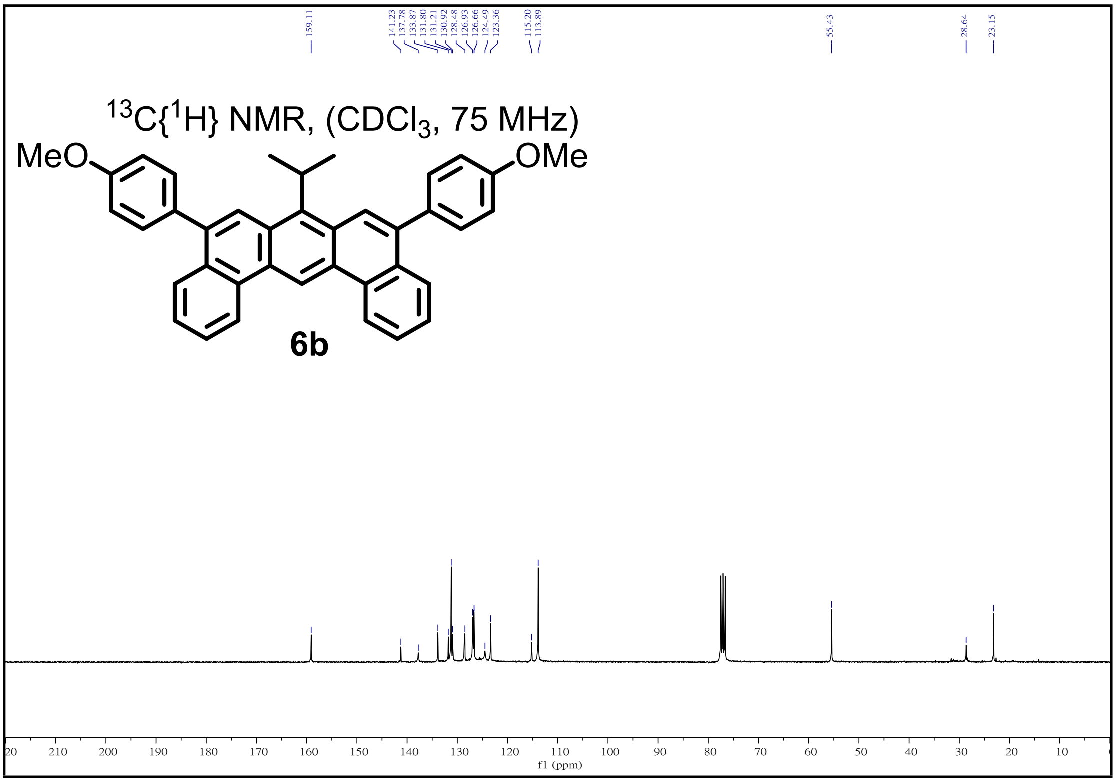

^{13}C{^{1}H} NMR, (CDCl$_3$, 75 MHz)
MeO
OMe
6b
f1 (ppm)

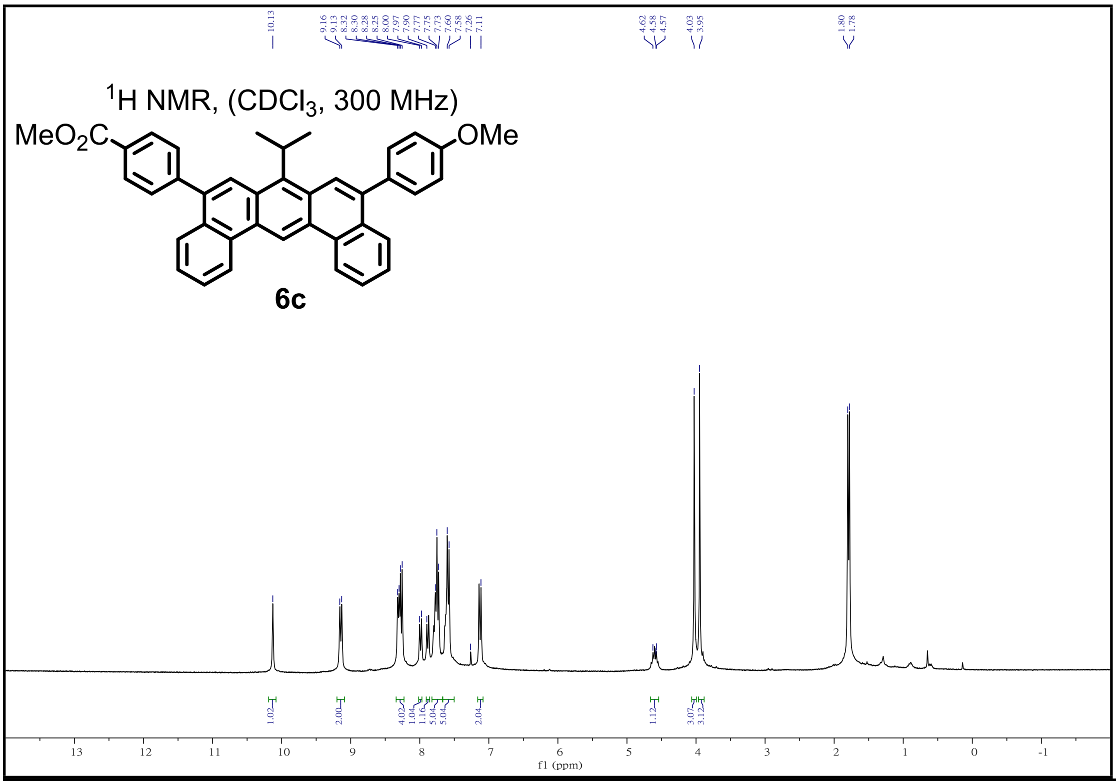

^{1}H NMR, (CDCl$_3$, 300 MHz)
MeO$_2$C
OMe
6c
f1 (ppm)

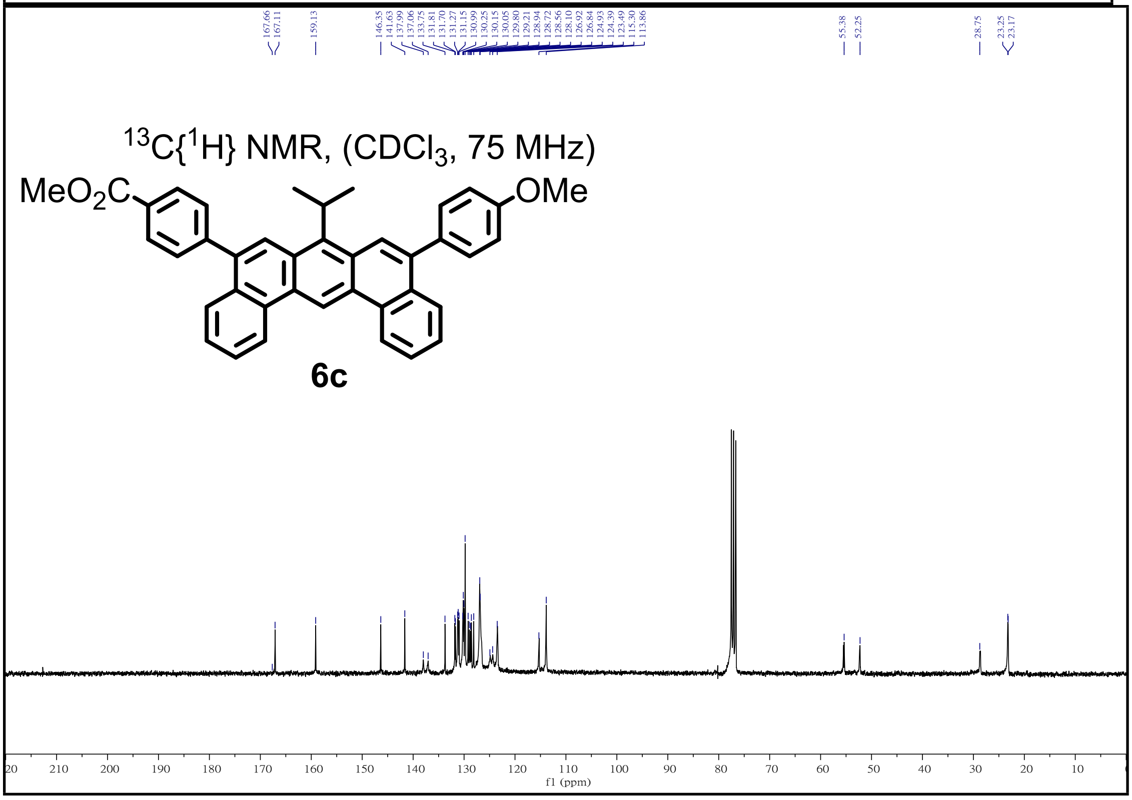

^{13}C{^{1}H} NMR, (CDCl$_3$, 75 MHz)
MeO$_2$C
OMe
6c
f1 (ppm)

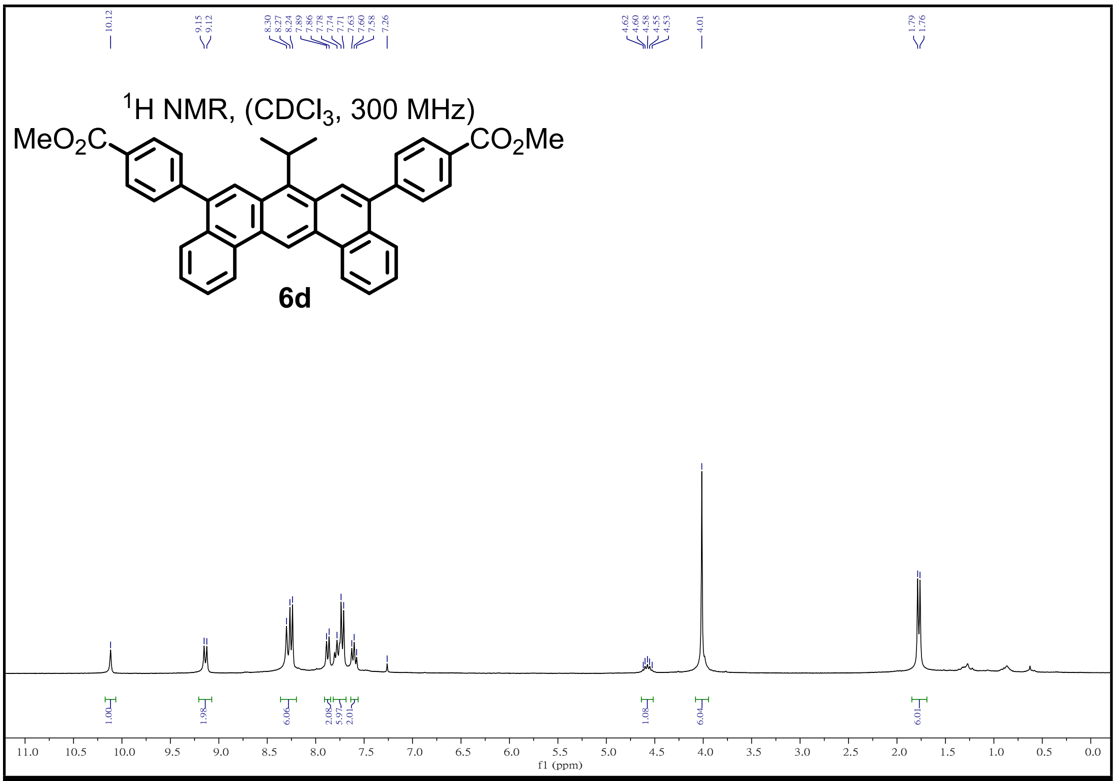

1H NMR, (CDCl3, 300 MHz)
MeO2C
CO2Me
6d
f1 (ppm)

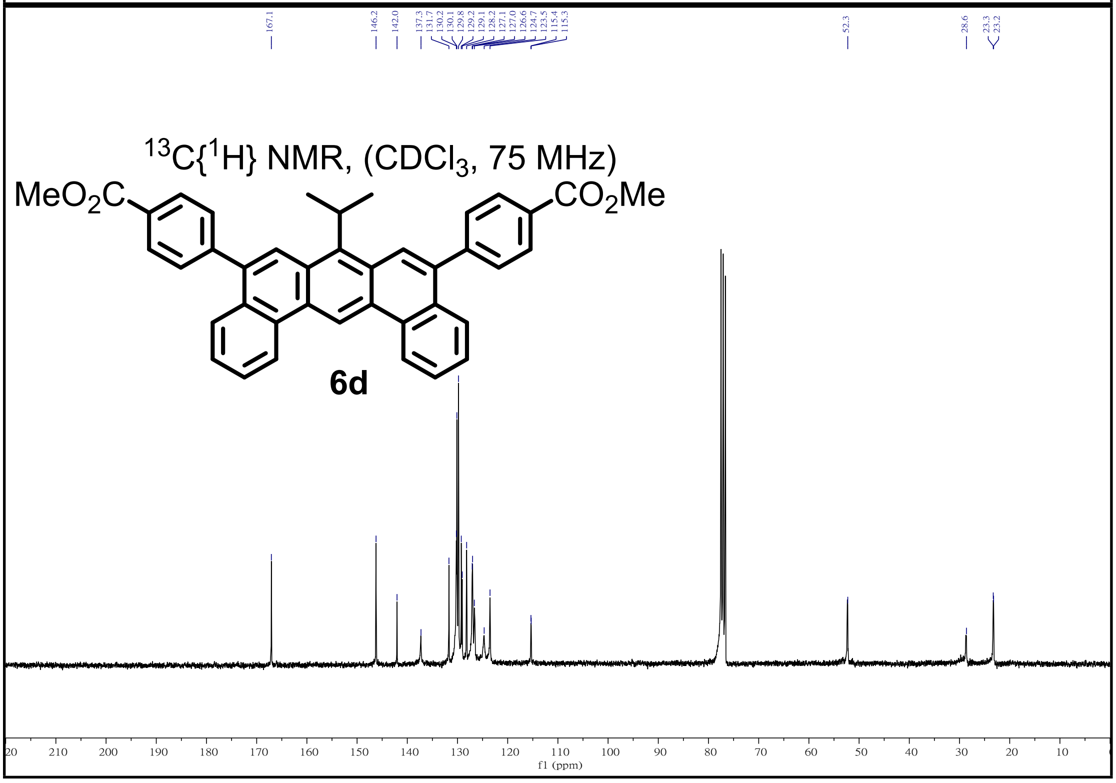

13C{1H} NMR, (CDCl3, 75 MHz)
MeO2C
CO2Me
6d
f1 (ppm)

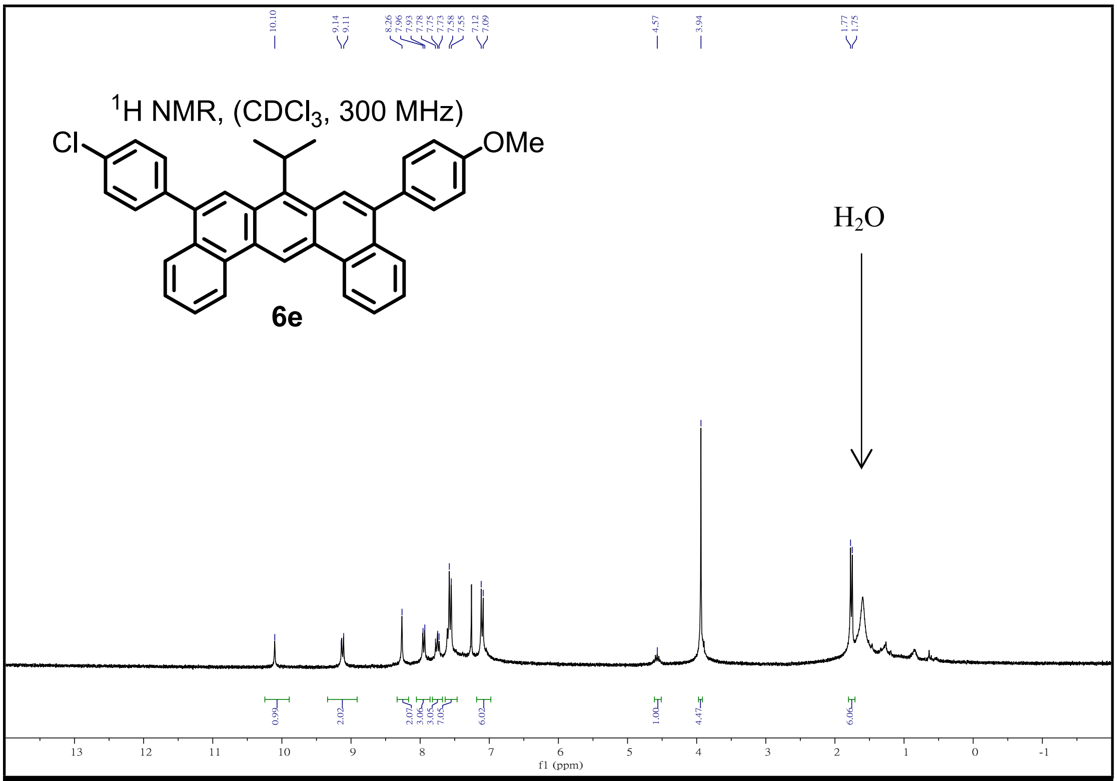
10.10
9.14
9.11
8.26
7.96
7.93
7.78
7.75
7.73
7.58
7.55
7.12
7.09
4.57
3.94
1.77
1.75
1H NMR, (CDCl3, 300 MHz)
Cl
OMe
6e
H2O
0.99
2.02
2.07
3.06
3.05
7.02
6.02
1.00
4.47
6.06
f1 (ppm)

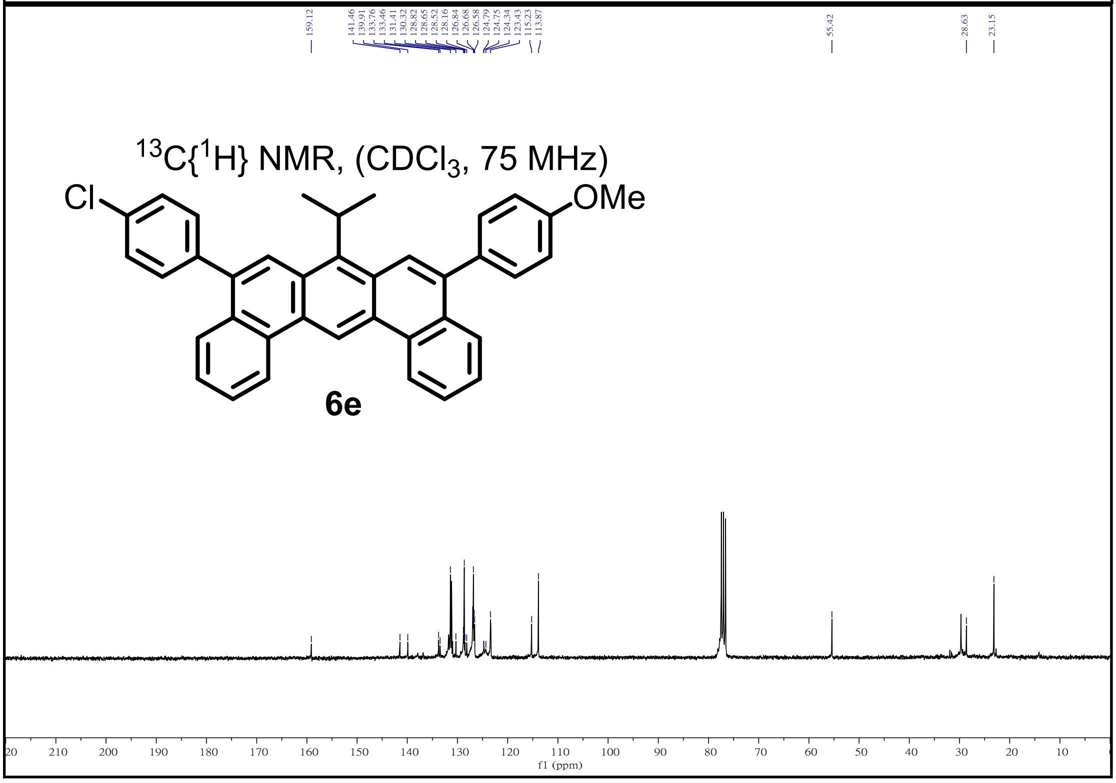
159.12
141.46
139.91
133.76
133.46
131.41
130.32
128.82
128.65
128.52
128.16
126.84
126.68
126.58
124.79
124.75
124.34
123.43
115.23
113.87
55.42
28.63
23.15
13C{1H} NMR, (CDCl3, 75 MHz)
Cl
OMe
6e
f1 (ppm)

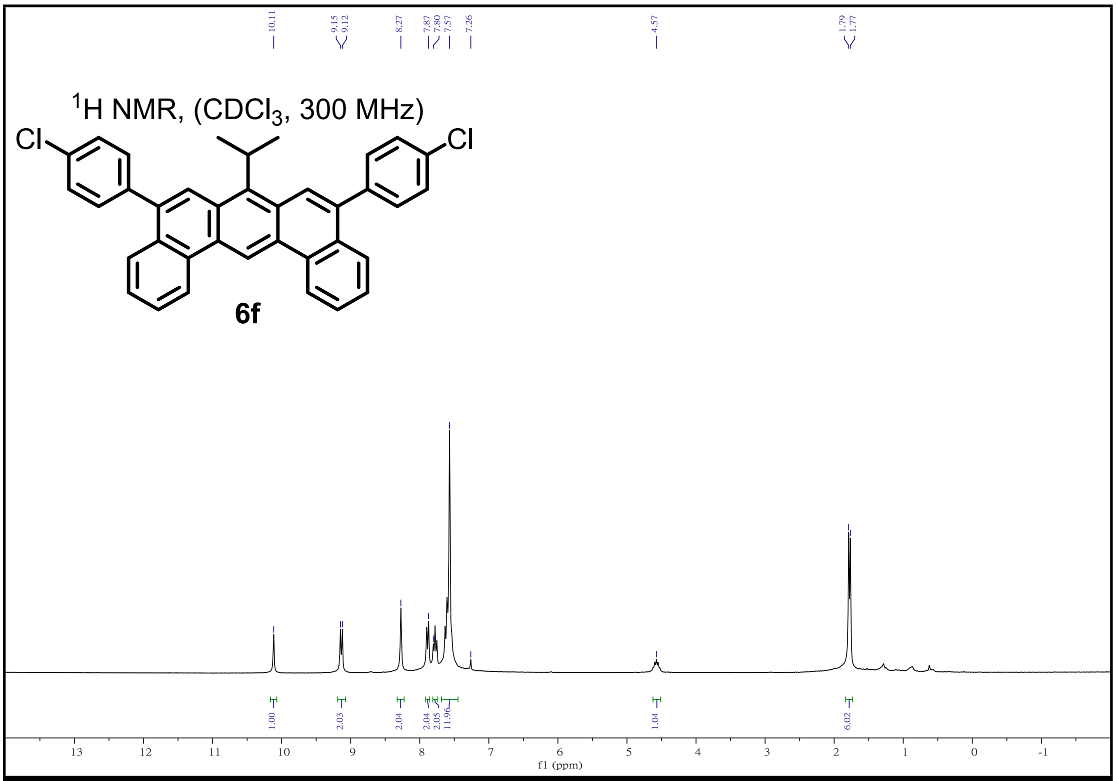
10.11
9.15
9.12
8.27
7.87
7.80
7.57
7.26
4.57
1.79
1.77
^{1}H NMR, ($CDCl_3$, 300 MHz)
Cl
Cl
6f
1.00
2.03
2.04
2.04
2.05
11.96
1.04
6.02
13
12
11
10
9
8
7
6
5
4
3
2
1
0
-1
f1 (ppm)

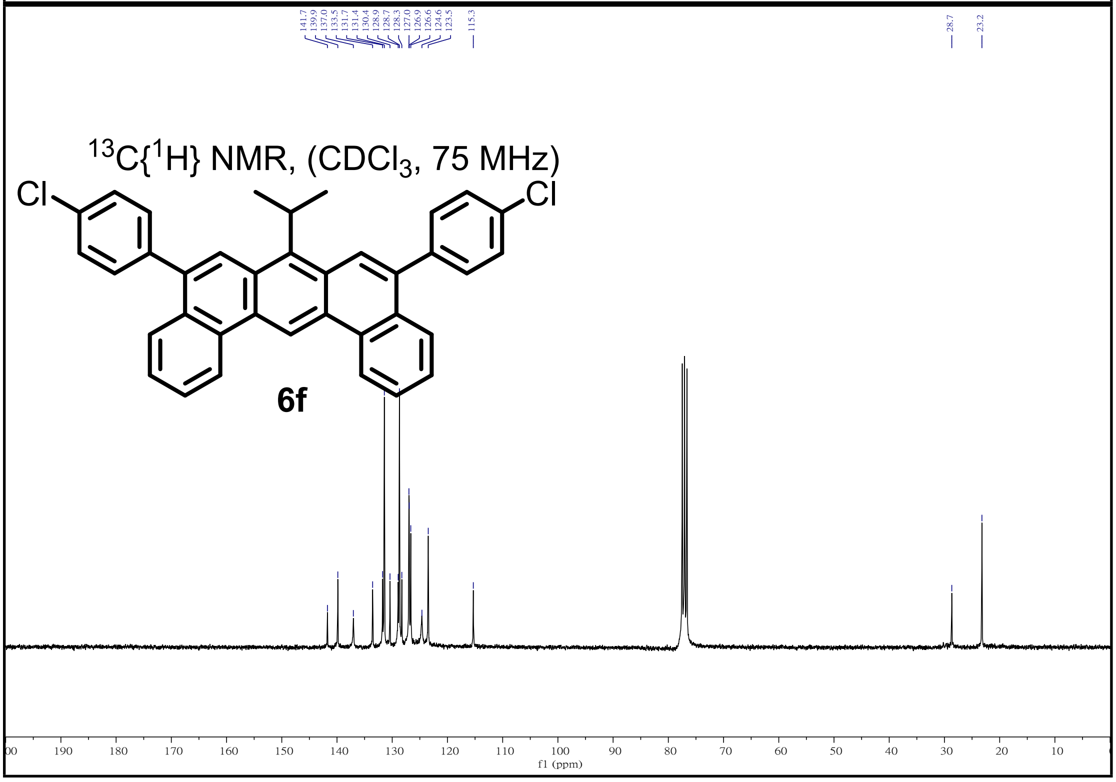
141.7
139.9
137.0
133.5
131.7
131.4
130.4
128.9
128.7
128.3
127.0
126.9
126.6
124.6
123.5
115.3
28.7
23.2
^{13}C{^{1}H} NMR, ($CDCl_3$, 75 MHz)
Cl
Cl
6f
190
180
170
160
150
140
130
120
110
100
90
80
70
60
50
40
30
20
10
f1 (ppm)

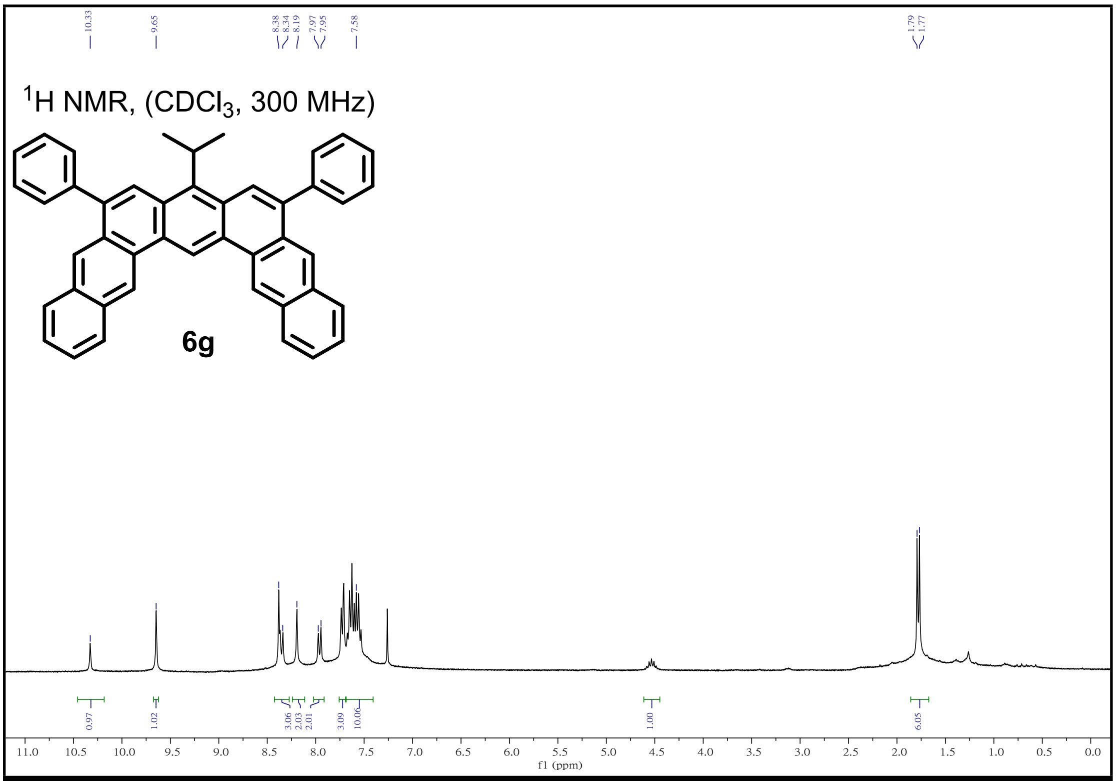
10.33
9.65
8.38
8.34
8.19
7.97
7.95
7.58
1.79
1.77
^{1}H NMR, (CDCl$_3$, 300 MHz)
6g
0.97
1.02
3.06
2.03
2.01
3.09
10.06
1.00
6.05
f1 (ppm)

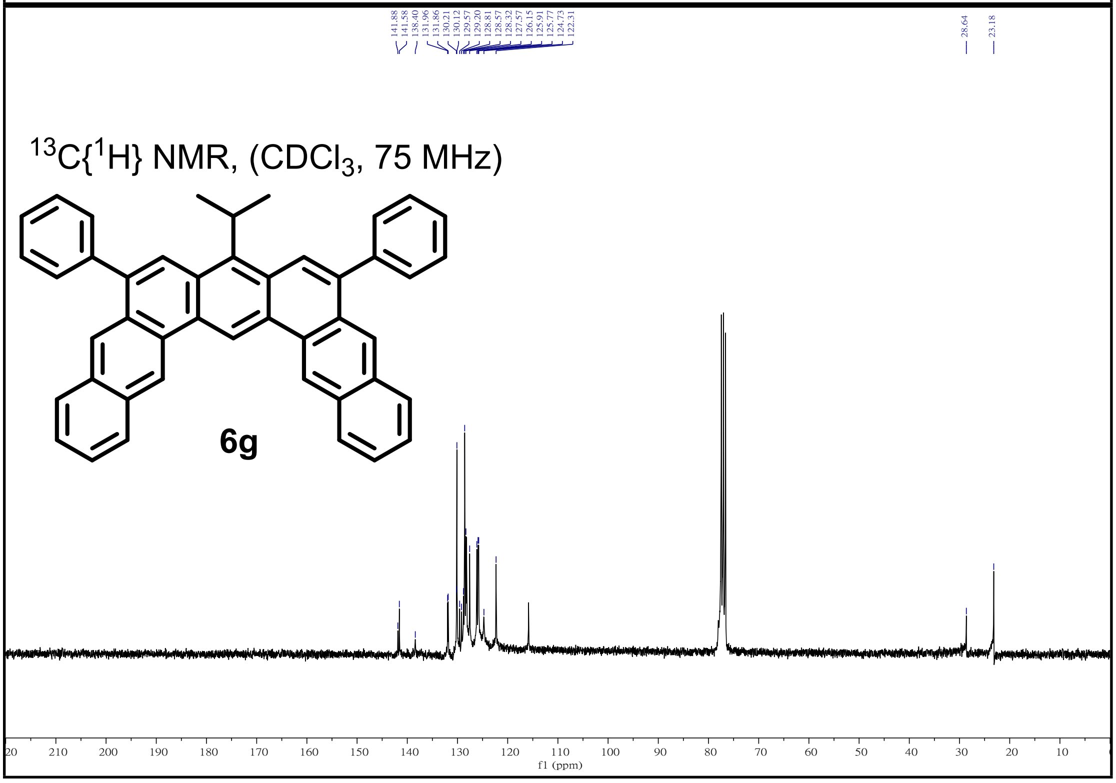
141.88
141.58
138.40
131.96
131.86
130.21
130.12
129.57
129.20
128.81
128.57
128.32
127.57
127.57
126.15
125.91
125.77
124.73
122.31
28.64
23.18
^{13}C{^{1}H} NMR, (CDCl$_3$, 75 MHz)
6g
f1 (ppm)

References

1. Wang, Y. C.; Huang, Y. H.; Tsai, H. C.; Basha, R. S.; Chou, C. M. Palladium-Catalyzed Proaromatic C(Alkenyl)–H Olefination: Synthesis of Densely Functionalized 1,3-Dienes. *Org, Lett*. **2020**, *22*, 6765–6770.

2. Chang, C.-H.; Chou, C.-M. Palladium-Catalyzed Decarboxylative γ-Olefination of 2,5-Cyclohexadiene-1-carboxylic Acid Derivatives with Vinyl Halides. *Org. Lett*. **2018**, *20*, 1949−1952.

3. Gaussian 09, Revision D.01, Frisch, M.J.; Trucks, G.W.; Schlegel, H.B.; Scuseria, G.E.; Robb, M.A.; Cheeseman, J.R.; Scalmani, G.; Barone, V.; Mennucci, B.; Petersson, G.A.; Nakatsuji, H.; Caricato, M.; Li, X.; Hratchian, H.P.; Izmaylov, A.F.; Bloino, J.; Zheng, G.; Sonnenberg, J.L.; Hada, M.; Ehara, M.; Toyota, K.; Fukuda, R.; Hasegawa, J.; Ishida, M.; Nakajima, T.; Honda, Y.; Kitao, O.; Nakai, H.; Vreven, T.; Montgomery, J.A., Jr.; Peralta, J.E.; Ogliaro, F.; Bearpark, M.; Heyd, J.J.; Brothers, E.; Kudin, K.N.; Staroverov, V.N.; Kobayashi, R.; Normand, J.; Raghavachari, K.; Rendell, A.; Burant, J.C.; Iyengar, S.S.; Tomasi, J.; Cossi, M.; Rega, N.; Millam, N.J.; Klene, M.; Knox, J.E.; Cross, J.B.; Bakken, V.; Adamo, C.; Jaramillo, J.; Gomperts, R.; Stratmann, R.E.; Yazyev, O.; Austin, A. J.; Cammi, R.; Pomelli, C.; Ochterski, J. W.; Martin, R.L.; Morokuma, K.; Zakrzewski, V.G.; Voth, G.A.; Salvador, P.; Dannenberg, J.J.; Dapprich, S.; Daniels, A.D.; Farkas, Ö.; Foresman, J.B.; Ortiz, J.V.; Cioslowski, J.; Fox, D.J. Gaussian, Inc., Wallingford CT, **2009**.
